# Ordered co-encapsulation of chloride with polar neutral guests in a tetraurea calix[4]pyrrole dimeric capsule†
†Electronic supplementary information (ESI) available: ^1^H and 2D NMR experiments. CCDC 1405136. For ESI and crystallographic data in CIF or other electronic format see DOI: 10.1039/c5sc02024g


**DOI:** 10.1039/c5sc02024g

**Published:** 2015-07-20

**Authors:** Albano Galán, Virginia Valderrey, Pablo Ballester

**Affiliations:** a Institute of Chemical Research of Catalonia (ICIQ) , Avgda. Països Catalans 16 , 43007 Tarragona , Spain . Email: pballester@iciq.es; b Catalan Institution for Research and Advanced Studies (ICREA) , Passeig Lluís Companys 23 , 08010 Barcelona , Spain

## Abstract

In this work, we describe the stoichiometrically controlled self-assembly process of tetraurea calix[4]pyrrole **1** with a polar neutral guest, trimethylamine *N*-oxide or beta-alanine betaine, and methyltrioctylammonium chloride (MTOACl) salt into two supramolecular architectures which differ in morphology and stoichiometry. Whereas an equimolar solution of tetraurea calixpyrrole **1**, polar guest and MTOACl produces a four-particle inclusion assembly, a mixture of the same components in a 2 : 1 : 1 molar ratio induces the formation of a dimeric capsular assembly displaying multiple guests which are orderly co-encapsulated. The influence of other polar guests and ammonium salts on the self-assembly process is also described.

## Abstract 2

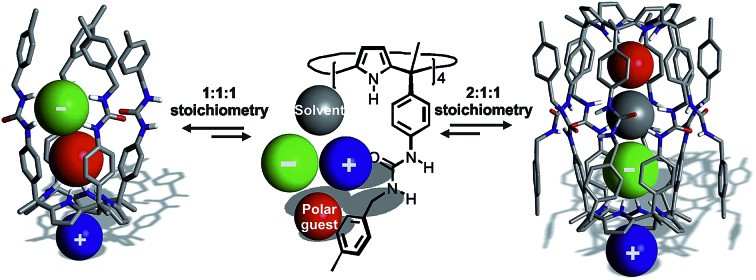
A calix[4]pyrrole tetraurea, a polar guest and methyltrioctylammonium chloride quantitatively self-assemble in two different complexes in response to the components' stoichiometry.

## Introduction

Self-assembled molecular capsules based on hydrogen bonding interactions are a well-known class of synthetic molecular containers. They enclose small spaces in which molecules are confined, contiguously enclosed and completely isolated from the bulk solvent. However, the extensive use of aromatic groups in the scaffolds of the capsule’s components challenges the inclusion of polar groups that can be presented to the encapsulated guests.^1^–^5^ Consequently, the interiors of most hydrogen bonded capsules do not have polar binding sites and are simply size^6^ and shape^7^ complementary to one or multiple encapsulated neutral guests that usually lack polar functional groups. By the same token, the attractive interactions that exist between the encapsulated guests and the container walls are weak.^8^ This allows the former to rotate,^9^ tumble^10^,^11^ or even exchange positions^12^ within the limited space provided by the latter at rates that are usually fast on the NMR timescale. When steric effects restrict some of the guests' motions, the non-ordered nature of the encapsulated complexes is revealed through NMR spectroscopy. In fact, the emergence of social^10^ and constellational^13^ isomers in encapsulation complexes of multiple guests results from the absence of directional interactions in the capsule's interior.

In contrast, biological receptors contain a mixture of polar and hydrophobic residues converging in their binding sites. The formation of ordered encapsulation complexes and the encapsulation of polar guests require the inclusion of polar functions in the cavity of the container.^14^ This approach has the added benefit of increasing the selectivity and thermodynamic stability of the formed capsular aggregates. The decoration of the internal cavities of molecular containers with polar groups has been achieved to a reasonable extent for receptors having a purely covalent structure.^8^ On the contrary, the decoration of the interior of hydrogen-bonded supramolecular containers with polar groups is still in its infancy.^15^–^20^

A few years ago, we reported that in CDCl_3_ solution tetraurea aryl-extended calix[4]pyrrole **1** dimerizes *via* encapsulating one molecule of 4,4′-bipyridine-*N*,*N*′-dioxide **2** to yield the capsular assembly **2**⊂**1**_2_ stabilized by a cyclic array of 16 hydrogen bonds.^21^ The dimeric capsule **1**_2_ featured a polar interior and established multiple directional interactions (hydrogen bonds) with the encapsulated guest. Later on, we demonstrated the pairwise encapsulation of trimethylamine *N*-oxide **3** and trimethylphosphine oxide **4** in the same dimeric molecular container **1**_2_.^20^ The inclusion of polar functions in the cavity of **1**_2_ was also responsible for the selective binding of **3** and the formation of ordered encapsulation complexes, (**3**·**3**)⊂**1**_2_ and (**3**·**4**)⊂**1**_2_, respectively.

In non-polar solvents, calix[4]pyrroles are known to function as heteroditopic receptors for ion-pairs.^22^,^23^ The calix[4]pyrrole unit binds anions by establishing four convergent hydrogen bonds with the pyrrole NHs. Upon anion binding, the calix[4]pyrrole core adopts a cone conformation creating a shallow aromatic cavity opposite to the bound anion. The aromatic rings that shape this cavity are electron-rich making it an ideal site for the recognition of cations that are size and shape complementary.^24^ On the other hand, the hydrogen bond donor properties of the NHs in the *syn*–*syn* form of the urea group have been extensively exploited for the recognition of anions.^25^ Accordingly, tetraurea calix[4]pyrrole **1** bearing two different binding sites for anions and one binding site for cations undoubtedly qualifies as a multitopic ion-pair receptor.^26^,^27^

In this work, we describe the stoichiometrically controlled self-assembly process of tetraurea calix[4]pyrrole **1** with a polar neutral guest, trimethylamine *N*-oxide **3** or beta-alanine betaine **12**, and methyltrioctylammonium chloride salt (MTOACl, **6**), in two diverse supramolecular architectures which differ in morphology and stoichiometry. On the one hand, we observed that **1** dimerized through co-encapsulating the chloride anion and the polar guest to afford a five particle capsular assembly (Cl^–^·polar guest)⊂**1**_2_·MTOA^+^ when a 2 : 1 : 1 molar ratio of the components (tetraurea **1**, polar guest and MTOACl) was used. On the other hand, a mixture of the components in a 1 : 1 : 1 molar ratio produced exclusively a four particle inclusion complex, polar guest⊂**1**·MTOACl.

## Results and discussion

### Formation of a four-particle assembly between tetraurea 1, pyridine *N*-oxide and ion-pairs

The ^1^H NMR spectrum of tetraurea **1** in non-polar solvents like CDCl_3_ or CD_2_Cl_2_ shows broad and ill-resolved signals (Fig. S1†). This is the result of aggregation phenomena induced by intermolecular hydrogen bonding interactions between the urea groups. Molecular modelling studies (MM3) assigned a packing coefficient value of 50% to the pairwise encapsulation complex of pyridine *N*-oxide **5** in the hydrogen-bonded capsule, (**5**·**5**)⊂**1**_2_. We calculated a similar packing coefficient for the encapsulation of the capsular assembly **2**⊂**1**_2_.^21^ Surprisingly to us, the addition of an equimolar amount of pyridine *N*-oxide **5** to CDCl_3_ or CD_2_Cl_2_ suspensions of tetraurea **1** produced the formation of a white precipitate.‡
‡The ^1^H NMR analysis of the liquid phase testified the total absence of detectable proton signals. The solid precipitate was filtered and dissolved in DMSO-*d*_6_. The ^1^H NMR spectrum of the solution indicated the presence of diagnostic signals for **1** and **5**, free in solution, in an exact 1 : 1 ratio. We hypothesized that pyridine *N*-oxide **5** was indeed included in the deep aromatic cavity of tetraurea **1** but the resulting **5**⊂**1** complex did not dimerize to form the expected (**5**·**5**)⊂**1**_2_ capsular assembly. Instead, the initially formed **5**⊂**1** complex experienced a strong aggregation process, probably mediated by hydrogen bonding interactions between their urea groups, yielding polymeric aggregates that precipitated out of solution. Inspired by the concept of tuning the sol–gel properties of urea derivatives through anion binding,^28^ we considered the use of a tetraalkylammonium chloride salt to disrupt the urea–urea hydrogen bonding interactions and redissolve the white precipitate.

The addition of one equivalent of a tetraalkylammonium chloride salt (MTOACl, **6** or TBACl, **7**) to the liquid samples (CD_2_Cl_2_ or CDCl_3_ solvent) containing the white precipitate, produced a transparent solution after shaking the mixture for several minutes. The ^1^H NMR spectrum of the solution showed sharp proton signals indicative of the formation of a well-defined assembly with *C*_4v_ symmetry (Fig. 2a). All of the proton signals were easily assigned using 2D NMR experiments. The pyrrolic NH protons (H^c^) of **1** were downfield shifted. In contrast, the protons *ortho* and *meta* (H^1^ and H^2^) with respect to the nitrogen atom of the bound pyridine *N*-oxide **5** moved upfield with respect to the corresponding signals in free **5**. Taken together, these observations indicated the inclusion of **5** in the deep aromatic cavity of the cone conformation of **1**. The inclusion process was driven by the formation of four hydrogen bonds between the oxygen atom of the *N*-oxide **5** and the pyrrole NHs of **1**. However, the location and binding geometry of the ammonium chloride salt (ion-pair) in the formed aggregate remained to be clarified. In the specific case of MTOACl **6**, we noticed that the *N*-methyl group of the organic cation appeared at *δ* = 0.74 ppm (Δ*δ* = –2.6 ppm). This intense upfield shift supported its inclusion in the shallow π-cavity offered by the cone conformation of **1**, opposite to the included *N*-oxide **5** (Fig. S5†).^29^ The intermolecular nOe cross peaks observed, in a 2D ROESY experiment of the aggregate, between the methyl and methylene protons alpha to the nitrogen atom in the MTOA cation and the beta-pyrrolic protons of **1** H^d^ were in complete agreement with the placement of the MTOA cation (Fig. S6 and S7†). In non-polar solvents, like CDCl_3_ and CD_2_Cl_2_, and at the millimolar concentrations used to perform the ^1^H NMR experiments, the chloride alkylammonium salts are not significantly dissociated.^30^ For this reason the formed aggregate involving the MTOA cation must also be ion-paired. We observed that the signals corresponding to the NHs of the urea groups (H^g^, H^h^) in the formed aggregate appeared downfield shifted compared to the signals in the free mono-urea reference compound **9** (Fig. S13†). This observation indicated their involvement in hydrogen bonding interactions. We located the bound chloride anion hydrogen-bonded to the urea groups at the upper rim of the aryl extended tetraurea calix[4]pyrrole **1**. The *C*_4v_ symmetry of the aggregate indicated a fast chemical exchange between the free and bound urea arms on the NMR timescale. Based on the integration values of selected proton signals, we assigned a 1 : 1 : 1 stoichiometry to the aggregate. The morphology of the aggregate **5**⊂**1**·MTOA^+^·Cl^–^ is that of an inclusion complex displaying a receptor separated binding geometry for the ion-pair. The use of other alkyl ammonium salts *i.e.* TBACl (**7**) and MTOABr (**8**) produced identical results (Fig. S3†). Conversely, TBAPF_6_ comprising a non-hydrogen bonding competitive anion was not effective in dissolving the precipitate. In short, the cation effect is not perceptible in the formation of the 1 : 1 : 1 complex **5**⊂**1**·MTOA^+^·Cl^–^ from the precipitate but the use of hydrogen bonding competitive anions is required in order to disrupt the aggregation between the urea groups. A DOSY NMR experiment performed on a CDCl_3_ equimolar solution of tetraurea **1**, pyridine *N*-oxide **5** and MTOACl **6** provided an identical diffusion coefficient value (4.3 ± 0.1 × 10^–10^ m^2^ s^–1^) for the three counterparts that is significantly smaller than for the free counterparts (Fig. S56†). This observation supported the involvement of **1**, **5** and **6** in a larger but unique aggregate.

**Fig. 1:**
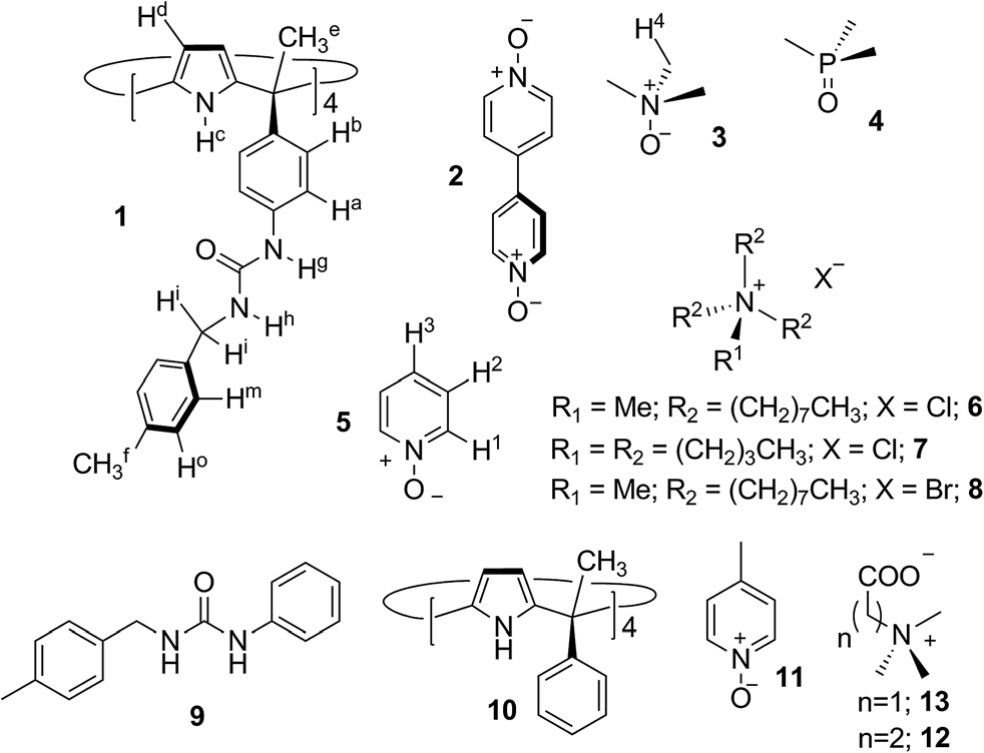
Line-drawing structures of the tetraurea calix[4]pyrrole receptor **1**, *N*-oxides **2**, **3**, **5** and **11**, *P*-oxide **4**, the series of tetraalkylammonium salts **6–8** employed as guests, the model systems of the binding sites **9** and **10**, and the betaines **12** and **13** also used as guests.

**Fig. 2:**
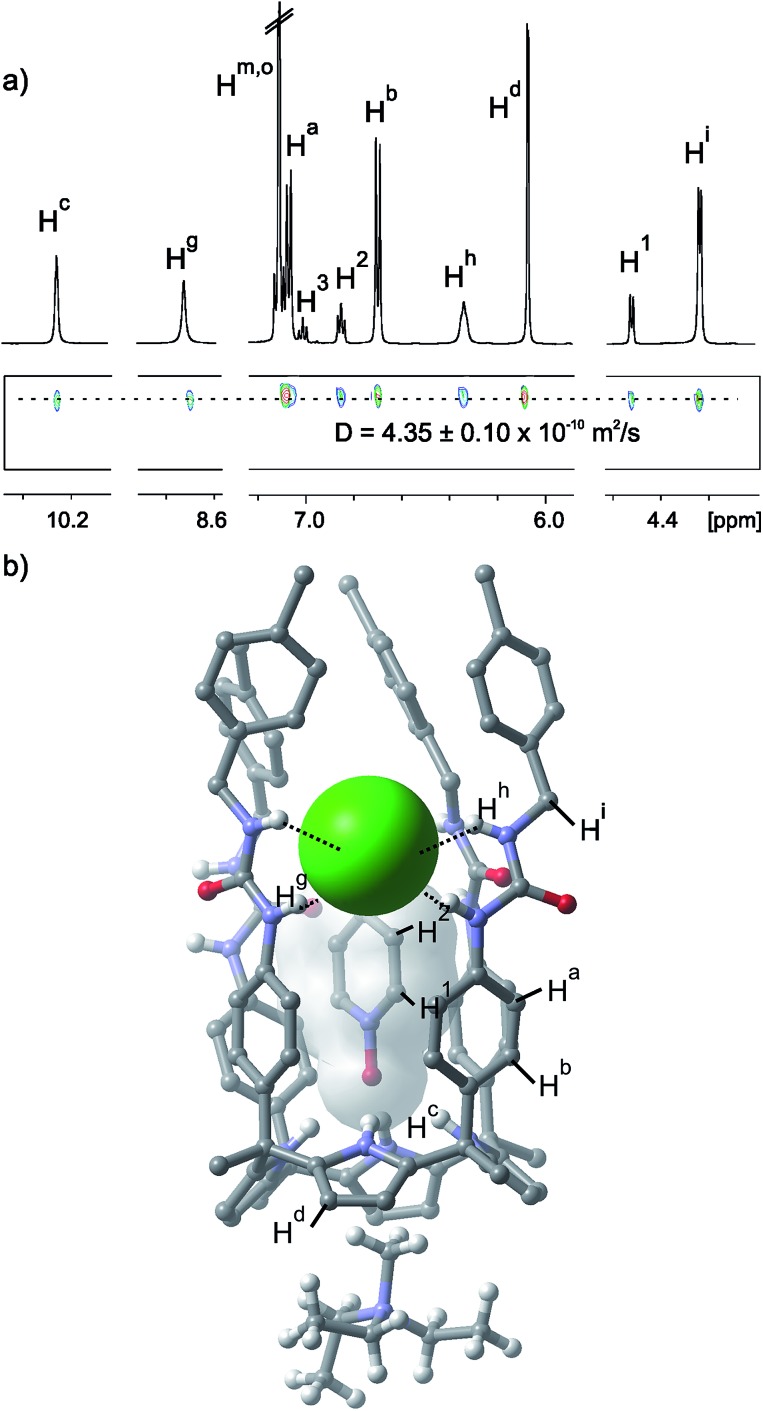
(a) Selected regions of the ^1^H and pseudo-2D DOSY plot NMR spectra of a CD_2_Cl_2_ solution of an equimolar mixture of tetraurea **1**, pyridine *N*-oxide **5** and MTOACl **6**. See Fig. 1 for the proton assignment. (b) X-ray structure of the four particle assembly **5**⊂**1**·MTOA^+^·Cl^–^. Non polar hydrogens of **1** and **5** are removed for clarity. Octyl substituents of the MTOA cation are truncated to ethyl groups also for simplicity.

The binding geometry of the four particle aggregate **5**⊂**1**·MTOA^+^·Cl^–^ proposed in solution was fully supported by the X-ray diffraction analysis of a single crystal grown from chloroform. The crystal structure shows the pyridine *N*-oxide **5** hydrogen-bonded and included deep in the calix[4]pyrrole cavity (Fig. 2b). The *N*-methyl group of the MTOA cation is located in the shallow electron-rich cup provided by the calixpyrrole cone conformation. Three of the four urea groups are oriented in the same sense of rotation and the chloride is bound to two adjacent urea groups oriented in the opposite sense of rotation by means of the formation of four hydrogen bonds.

### Assessment of the binding affinities of urea and calix[4]pyrrole units towards the pyridine *N*-oxides and tetraalkylammonium chloride salts

We selected *N*-benzyl-*N*′-phenyl urea **9** and the α,α,α,α-tetraphenyl calix[4]pyrrole **10** as model systems for assessing the binding affinities of pyridine-*N*-oxide **5**,§
§Solubility problems experienced in the titration experiments of calix[4]pyrrole **10** dictated the replacement of **11** with pyridine *N*-oxide **5**. MTOACl **6** and TBACl **7**, in CDCl_3_ solution towards the two different hydrogen bonding sites present in the multitopic receptor **1**.

Using ^1^H NMR titration experiments we calculated the values of the binding constants for the 1 : 1 complexes formed between the urea **9** and the three guests. The affinity of calix[4]pyrrole **10** for TBACl was also determined using NMR spectroscopy (Fig. S13–S24†). In contrast, the accurate determination of the large binding constant values for the 1 : 1 complexes of calix[4]pyrrole **10** with pyridine *N*-oxide **11** and MTOACl **6** required isothermal titration calorimetry (ITC) experiments. The calculated binding constant values are summarized in Table 1.

**Table 1:** Binding constant values (M^–1^) determined for the 1 : 1 complexes of urea **9** and calix[4]pyrrole **10** with *N*-oxide **11**, and the tetraalkylammonium chloride salts **6** (MTOACl) and **7** (TBACl)

Host	Guests		
	**11**	**6**	**7**
**9**	30^*a*^	800^*a*^	800^*a*^
**10**	1 × 10^6^ ^*b*^	2800^*b*^	20^*a*^

^*a*^ ^1^H NMR titration.

^*b*^ITC experiment.

The relative order of interaction strengths measured for the 1 : 1 complexes of the model receptors **9** and **10** with *N*-oxide **11**, and MTOACl **6** (Table 1) is in complete agreement with the binding geometry present in the **5**⊂**1**·MTOA^+^·Cl^–^ complex: (a) preferential inclusion of the pyridine *N*-oxide **5** in the deep aromatic cavity of the calix[4]pyrrole **1** and (b) higher affinity of the chloride ion-pairs (MTOACl) for the urea groups. The high selectivity in binding MTOACl, in comparison to TBACl, displayed by calix[4]pyrrole **10** is derived from the known heteroditopic nature of this type of receptors^22^,^31^,^32^ for ion-pair binding. Conversely, urea **9** being a monotopic anion receptor exclusively recognized the chloride and did not show any sign of selectivity in the binding of MTOACl **6***vs.* TBACl **7**.

### Assembly of a dimeric capsule of tetraurea **1** induced by the co-encapsulation of trimethylamine *N*-oxide and chloride

The addition of 1 equiv. of MTOACl to a CDCl_3_ suspension of tetraurea **1** produced a transparent solution, but contrary to our expectations it did not induce the formation of a capsular aggregate *i.e.* (Cl^–^·Cl^–^)⊂**1**_2_·(MTOA^+^)_2_. The observation of broad signals in the ^1^H NMR spectrum of the mixture hinted at the formation of stoichiometrically and/or structurally ill-defined aggregates. This negative result prompted us to study the dimerization of **1** induced through co-encapsulation of a suitable *N*-oxide and the chloride anion. Recently, we reported the quantitative pairwise encapsulation of trimethylamine *N*-oxide **3** yielding a capsular assembly (**3**·**3**)⊂**1**_2_.^20^,^21^ Thus, we decided to investigate the use of trimethylamine *N*-oxide **3** as the co-encapsulation guest with chloride in the polar cavity of the **1**_2_ capsule. We expected that a capsular assembly of the type (*N*-oxide·Cl^–^)⊂**1**_2_·MTOA^+^ would allow the fine tuning of the cavity contents and also eliminate the plausible electrostatic repulsion between two negatively charged encapsulated guests. We considered that the preferential assembly of the (*N*-oxide·Cl^–^)⊂**1**_2_·MTOA^+^ encapsulation complex would require working under strict stoichiometric control of the components.

**Fig. 3:**
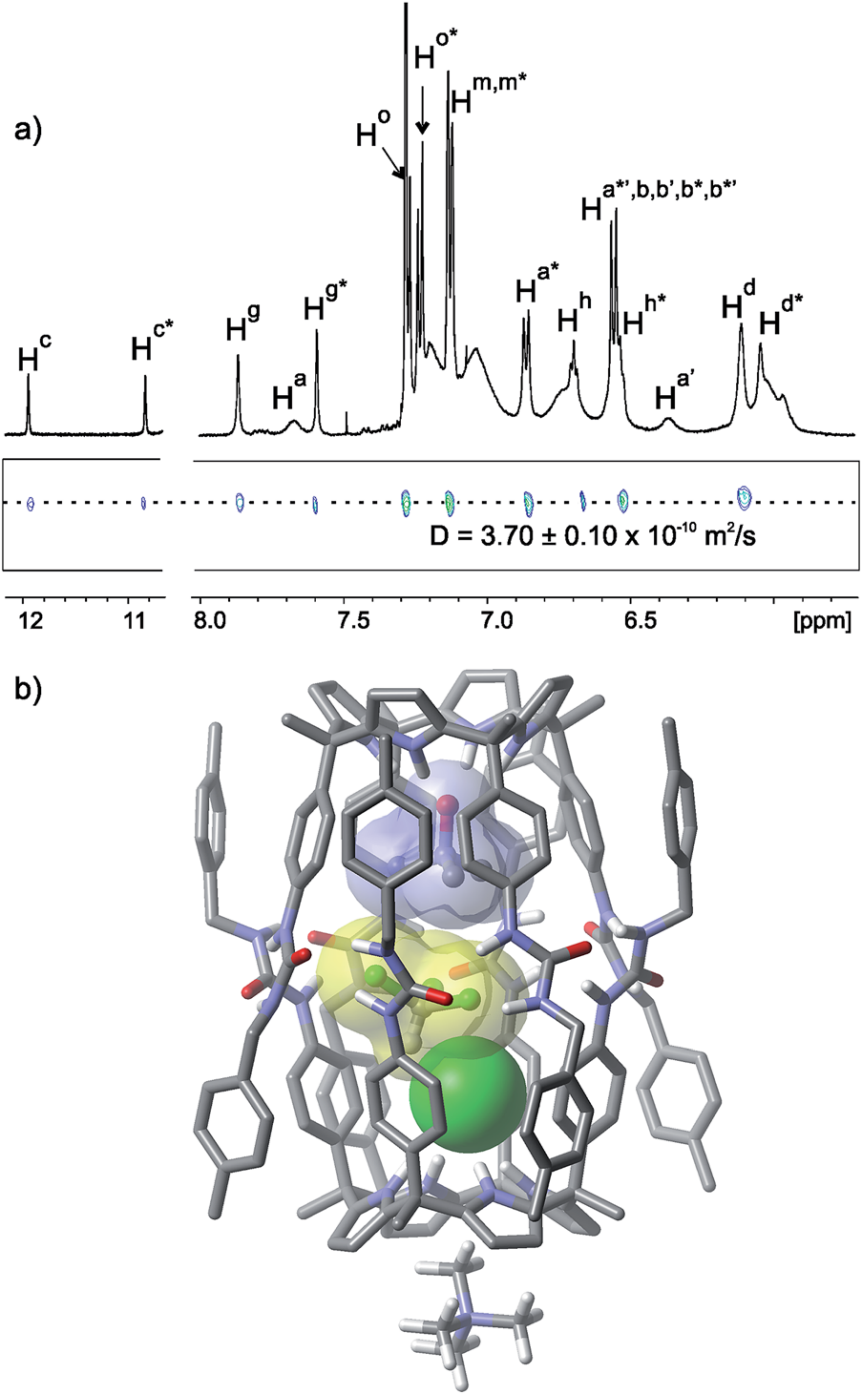
(a) Selected regions of the ^1^H and pseudo-2D DOSY plot NMR spectra of a CDCl_3_ solution of tetraurea **1**, trimethylamine *N*-oxide **3** and MTOACl **6** in a 2 : 1 : 1 molar ratio forming the capsular assembly (**3**·Cl^–^)⊂**1**_2_·MTOA^+^. See Fig. 1 for the proton assignment. Primed letters indicate diastereotopic protons. Letters marked with an asterisk indicate the protons of different hemispheres. (b) Energy minimized (MM3) structure of the capsular assembly (**3**·CHCl_3_·Cl^–^)⊂**1**_2_·MTOA^+^. The MTOA cation is depicted as tetramethylammonium (TMA) for clarity.

The ^1^H NMR spectrum (Fig. 3a) of an equimolar CDCl_3_ solution of **3** and MTOACl containing 2 equiv. of tetraurea **1** revealed the presence of sharp and well resolved signals that did not coincide with those of the encapsulation complex (**3**·**3**)⊂**1**_2_ (Fig. S28†). We observed two different signals for the hydrogen-bonded pyrrole NH protons of bound **1**. This was indicative of the complexation of **1** with two different guests. Both pyrrole NH signals were downfield shifted compared to the singlet detected for the same protons in the inclusion complex **5**⊂**1**·MTOA^+^·Cl^–^, obtained using pyridine *N*-oxide **5** instead of trimethylamine *N*-oxide **3**. In addition, the benzylic protons H^i^ of bound **1** split into diastereotopic signals. Two different sharp signals (*δ* = 7.87 and 7.60 ppm) were also visible for the NHs of the urea alpha to the *meso*-phenyl groups of **1** (H^g^). The chemical shift values of these NHs suggested their involvement in hydrogen bonding interactions. The methyl groups of **3** were upfield shifted at *δ* = 0.90 ppm and suggested that the position of the *N*-oxide was deep in the aromatic cavity of **1**. Taken together, these observations hinted at the formation of a capsular dimeric assembly (**3**·Cl^–^)⊂**1**_2_·MTOA^+^.

The co-encapsulation of two different guests produced a desymmetrisation of the capsular assembly **1**_2_ and rendered the two hemispheres chemically non-equivalent. Another independent element of asymmetry present in the (**3**·Cl^–^)⊂**1**_2_·MTOA^+^ capsular assembly was derived from the unidirectional orientation of the urea groups that was kinetically stable on the ^1^H NMR timescale. This resulted in the existence of the five particle capsular aggregate (**3**·Cl^–^)⊂**1**_2_·MTOA^+^ as a pair of enantiomers. The latter asymmetry was expressed by the observation of resolved diastereotopic signals for some of the aromatic protons belonging to the same hemisphere in **1**_2_. Other diastereotopic protons appeared as broad signals. Because the two hemispheres in (**3**·Cl^–^)⊂**1**_2_·MTOA^+^ are chemically non-equivalent, a total of eight diastereotopic signals can be expected for the *meso*-phenyl protons (H^a^ and H^b^) and four for the benzylic protons (H^i^). Nevertheless, proton signal overlapping and broadening hampered the observation of separate signals for the eight aromatic protons. The number of proton signals observed for the capsule was in agreement with a *C*_4_ symmetry, in contrast to the *C*_4v_ symmetry determined for the inclusion complex **5**⊂**1**·MTOA^+^·Cl^–^.

The *N*-methyl group of the co-bound MTOA cation resonated as a broad singlet at *δ* = 0.38 ppm testifying its inclusion in the *exo*-cavity defined by the four pyrrole rings opposite to the bound chloride. A ROESY experiment provided additional evidence for the capsule formation (Fig. S30 and S31†). We observed cross-peaks due to the close spatial proximity between the methyl protons of the encapsulated **3** and the pyrrole NH protons resonating at *δ* = 10.85 ppm. This allowed the assignment of the pyrrole NH protons appearing at *δ* = 11.96 ppm as the ones forming hydrogen bonds with the encapsulated chloride. It is worth noting that we did not observe any chemical exchange cross-peak between the pyrrole NH protons of the two hemispheres. This observation suggested that the exchange of positions of the encapsulated guests was slow on the EXSY timescale.

The calculation of the packing coefficient for the (**3**·Cl^–^)⊂**1**_2_·MTOA^+^ capsule assembly provided a value of 39%. Most likely, a molecule of the solvent was co-encapsulated with the two guests to achieve a packing coefficient closer to the optimum value of 55%. Indeed, the recalculation of the packing coefficient value considering the co-encapsulation of the three guests, **3**, Cl^–^ and CDCl_3_, returned a value of 58% (Fig. 3b). This triple encapsulation was also implied by differences in the proton signals of the ^1^H NMR spectra of the capsular aggregates (**3**·Cl^–^)⊂**1**_2_·MTOA^+^ recorded in CDCl_3_ or CD_2_Cl_2_ solutions that cannot be explained by a simple change in solvent (Fig. S28 and S29†). A 1D-GOESY NMR experiment performed at 253 K using a non-deuterated CHCl_3_ solution of tetraurea **1**, trimethylamine *N*-oxide **3** and MTOACl in a 2 : 1 : 1 molar ratio revealed a proton signal at *δ* = 6.60 ppm which was involved in a slow chemical exchange process with the bulk solvent (Fig. S32†). We assigned this signal to the proton of the molecule of chloroform that was co-encapsulated. The proton of the encapsulated CHCl_3_ molecule showed a reduced upfield shift (Δ*δ* = –0.66 ppm) compared to that experienced (Δ*δ* = –3.90 ppm) in its encapsulation by a tetraurea calix[4]pyrrole/tetraurea calix[4]arene bis[2]catenaned capsule.^33^ Nucleus independent chemical shift (NICS) calculations in related molecular capsules based on resorcin[4]arene scaffolds show that the magnetic shielding caused by the aromatic rings had a minimum effect in the middle region surrounded by the seam of hydrogen bonds.^34^,^35^ All together, these results indicate the co-encapsulation of three different guests in the capsular assembly of **1**_2_ producing a single constellational isomer^13^ owing to the directional interactions present in the cavity. The two polar guests, *N*-oxide **3** and chloride, occupied the polar ends of the container with the CHCl_3_ molecule sandwiched between them. Previous examples of constellational encapsulation isomers of dimeric capsules have always been produced as mixtures of isomers.^36^

Disappointingly, the ^1^H NMR spectra of CDCl_3_ or CD_2_Cl_2_ solutions of tetraurea **1**, pyridine *N*-oxide **5** and MTOACl also in a 2 : 1 : 1 molar ratio show sharp proton signals corresponding to the 1 : 1 : 1 **5**⊂**1**·MTOA^+^·Cl^–^ complex. Additional broad signals were also visible in the spectra of the mixture that were assigned to tetraurea **1** forming oligomeric aggregates (Fig. S10†).¶
¶The use of an internal standard revealed that the addition of two equivalents of tetraurea **1** to an equimolar mixture of **5** and MTOACl in CDCl_3_ solution reduced their concentration by approximately 25%. Most likely, the two guests are also involved in the formation of oligomeric aggregates. Most likely, the (**5**·Cl^–^)⊂**1**_2_·MTOA^+^ capsular assembly is not formed in solution due to a low packing coefficient. *In silico*, the co-encapsulation of a solvent molecule with the two guests **5** and the chloride disrupted the capsular structure.

The gratifying results, obtained with *N*-oxide **3**, stimulated us to assess the self-assembly properties of the system using a 1 : 1 : 1 molar ratio of trimethylamine *N*-oxide **3**, tetraurea **1**, and MTOACl. The analysis of the equimolar mixture using ^1^H NMR spectroscopy revealed the presence of a main set of proton signals for the bound tetraurea **1** that were almost coincident with those observed for the **5**⊂**1**·MTOA^+^·Cl^–^ complex (Fig. S33†). A minor set of proton signals corresponding to the capsular assembly (**3**·Cl^–^)⊂**1**_2_·MTOA^+^ were still visible. In short, the system self-sorted itself in the almost exclusive formation of the inclusion complex **3**⊂**1**·MTOA^+^·Cl^–^ in response to the equimolar mixture of components. Notably, the methyl protons for bound **3** in the **3**⊂**1**·MTOA^+^·Cl^–^ complex were upfield shifted at *δ* = 0.75 ppm. This chemical shift value was markedly different from that observed for the same methyl protons of bound **3** in the capsular assemblies (**3**·Cl^–^)⊂**1**_2_·MTOA^+^ (*δ* = 0.90 ppm) and (**3**·**3**)⊂**1**_2_ (*δ* = 0.54 ppm) and provided support for the presence of different magnetic environments. The diffusion coefficient values calculated for the two species, the capsular assembly (**3**·Cl^–^)⊂**1**_2_·MTOA^+^ and the inclusion complex **3**⊂**1**·MTOA^+^·Cl^–^, through performing DOSY experiments on CDCl_3_ solutions of tetraurea **1**, trimethylamine *N*-oxide and MTOACl with molar ratios of 2 : 1 : 1 and 1 : 1 : 1, respectively, were in complete agreement with their difference in size (Table 2, Fig. S57 and S58†).

**Table 2:** Chemical shift values of the protons of the pyrrolic NHs (H^c^) and the neutral guests in the characterized complexes. The complexation induced shifts (CISs) experienced by the guest protons in the complexes and the diffusion coefficient values of the latter are also tabulated^*a*^

Complex	*δ*s (ppm) H^c^	*δ*s (ppm) of the neutral bound guests	CIS (Δ*δ* (ppm)) of the neutral bound guests	Diffusion coeff. (×10^10^ m^2^ s^–1^)
**5**⊂**1**·MTOA^+^·Cl^–^	10.29	H^1^: 4.55	H^1^: –3.70	4.3 ± 0.1
		H^2^: 6.80	H^2^: –0.45	
(**3**·Cl^–^)⊂**1**_2_·MTOA^+^	11.96	H^4^: 0.90	H^4^: –2.40	3.7 ± 0.1
	10.85			
**3**⊂**1**·MTOA^+^·Cl^–^	10.69	H^4^: 0.75	H^4^: –2.55	4.0 ± 0.1
(**12**·Cl^–^)⊂**1**_2_·MTOA^+^	11.37	H^5^: –0.06	H^5^: –2.63	3.4 ± 0.1
	11.27	^a^H: 1.43	^a^H: –2.21	
		^b^H: 1.47	^b^H: –1.64	
**12**⊂**1**·MTOA^+^·Cl^–^	11.23	H^5^: 0.01	H^5^: –2.56	4.1 ± 0.1
		^a^H: 2.35	^a^H: –1.29	
		^b^H: 2.19	^b^H: –0.93	
(**13**·Cl^–^)⊂**1**_2_·MTOA^+^	11.42	^a^H: 0.70	^a^H: –3.02	3.6 ± 0.1
	10.37	^b^H: 1.92	^b^H: –1.31	
**13**⊂**1**·MTOA^+^·Cl^–^	10.71	^a^H: 0.84	^a^H: –2.88	4.1 ± 0.1
		^b^H: 2.02	^b^H: –1.21	

^*a*^ ^a^H: methylene protons alpha to the trimethylammonium group. ^b^H: methyl protons of the trimethylammonium group.

The change of MTOACl by TBACl eliminated the observed stoichiometric control of the self-assembly process. On the one hand, the equimolar mixture of trimethylamine *N*-oxide **3**, tetraurea **1**, and TBACl produced the exclusive formation of the **3**⊂**1**·TBA^+^·Cl^–^ complex. On the other hand, working under strict stoichiometric control (2 : 1 : 1 molar ratio) for obtaining the (**3**·Cl^–^)⊂**1**_2_·TBA^+^ capsular aggregate, we observed the presence of the encapsulation complex (**3**·**3**)⊂**1**_2_ in combination with other unassigned aggregates (Fig. S37†). In summary, while the cation of the chloride salt played an insignificant role in the formation of the inclusion complexes **3**⊂**1**·A^+^·Cl^–^, it was a key element for the assembly of the encapsulation aggregates (**3**·Cl^–^)⊂**1**_2_·A^+^.

Probably, the MTOACl is preferentially bound by tetraurea **1** in a host separated ion-pair geometry, Cl^–^⊂**1**·MTOA^+^, with the chloride included in the aromatic cavity of the calix[4]pyrrole and the *N*-methyl group of the co-bound cation located in the shallow aromatic cavity opposite to the bound anion. In this binding geometry, the urea arms of **1** in Cl^–^⊂**1**·MTOA^+^ are available to engage in hydrogen bonding interactions with their counterparts in the *N*-oxide inclusion complex, **3**⊂**1**. The net result being the assembly of the encapsulation complex (**3**·Cl^–^)⊂**1**_2_·MTOA^+^ as the almost exclusive species in solution.

Conversely, TBACl is bound better by the tetraurea **1** in a close-contact binding geometry, **1**·TBACl, through establishing hydrogen bonding interactions between the chloride and the urea groups (Table 1). That is to say, a direct competition for hydrogen bonding with the urea arms in **1** exists between the chloride in TBACl and the urea groups of the **3**⊂**1** complex. For this reason and to a certain extent the dimerization of the **3**⊂**1** complex yielding the encapsulation complex (**3**·**3**)⊂**1**_2_ competes with the oligomerization of **1**·TBACl. The result of the equilibria produced no detectable signals for the encapsulation complex (**3**·Cl^–^)⊂**1**_2_·TBA^+^.

### Assembly of dimeric capsules of tetraurea **1** induced by the co-encapsulation of betaines and chloride

We reasoned that certain betaines could also be appropriate co-encapsulation guests with chloride in the dimeric capsule **1**_2_. Specifically, the beta-alanine betaine **12** was a nice fit with respect to size, shape and chemical surface to the inner space of the capsule that was left after chloride encapsulation. Calix[4]pyrroles are known to be good receptors for carboxylates.^37^,^38^ The carboxylate group of **12** can establish hydrogen bonds with the *endo*-directed pyrrole NHs and the trimethylammonium group forms favorable coulombic interactions with the co-encapsulated chloride anion. We calculated a packing coefficient value for the encapsulation complex (**12**·Cl^–^)⊂**1**_2_·MTOA^+^ of 55%.

**Fig. 4:**
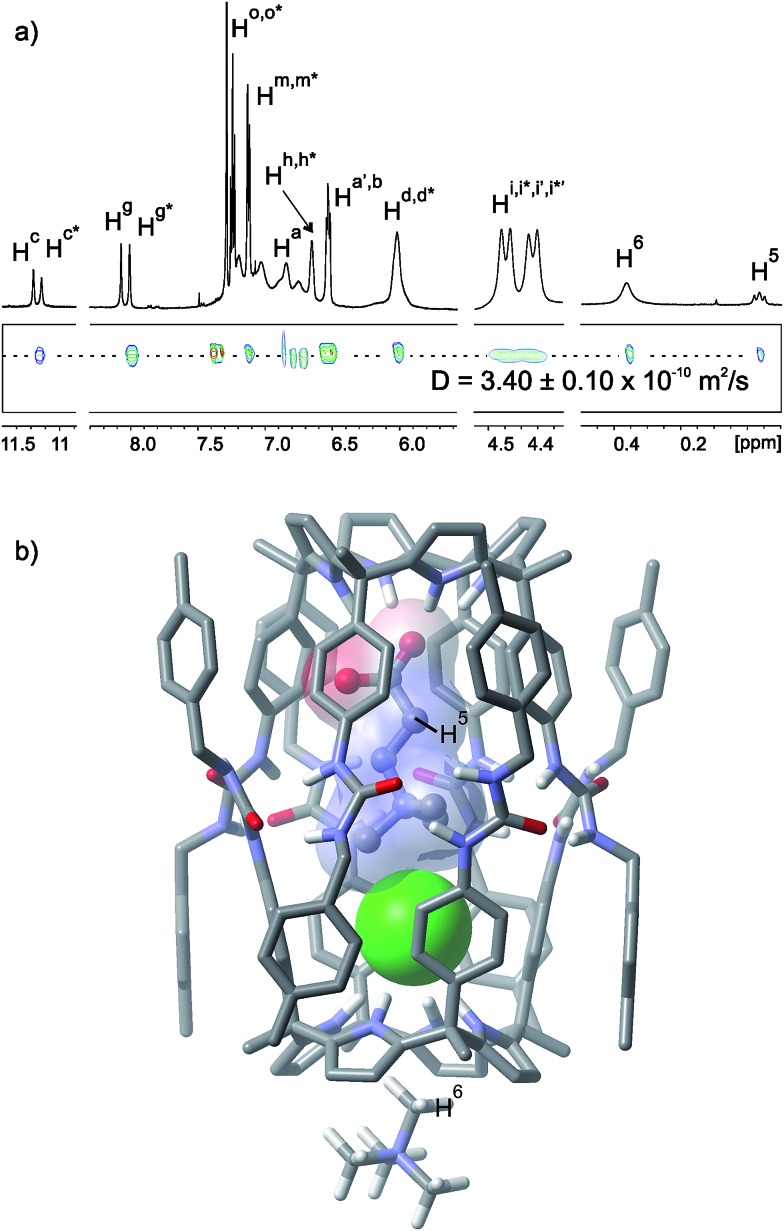
(a) Selected regions of the ^1^H and pseudo-2D DOSY plot NMR spectra of a CDCl_3_ solution of tetraurea **1**, betaine **12** and MTOACl in a 2 : 1 : 1 molar ratio forming the capsular assembly (**12**·Cl^–^)⊂**1**_2_·MTOA^+^. See Fig. 1 for the proton assignment. Primed letters indicate diastereotopic protons. Letters marked with an asterisk indicate the protons for different hemispheres. (b) Energy minimized (MM3) structure of the capsular assembly (**12**·Cl^–^)⊂**1**_2_·MTOA^+^. The MTOA cation is depicted as TMA for clarity.

A CDCl_3_ solution of tetraurea **1**, betaine **12** and MTOACl in a 2 : 1 : 1 molar ratio produced a ^1^H NMR spectrum with diagnostic signals of the formation of the encapsulation complex (**12**·Cl^–^)⊂**1**_2_·MTOA^+^ (Fig. 4a). In comparison to the co-encapsulation of chloride with trimethylamine *N*-oxide **3** in (**3**·Cl^–^)⊂**1**_2_·MTOA^+^, one of the NHs moved upfield and the other downfield (Table 2). This observation indicated that the carboxylate group of **12** formed stronger hydrogen bonds with the *endo*-calix[4]pyrrole binding sites than the oxygen atom of the *N*-oxide **3**. On the contrary, the chloride being also involved in electrostatic interactions with the nearby trimethylammonium group of **12** established weakened hydrogen bonds with the calix[4]pyrrole in the opposed hemisphere of the (**12**·Cl^–^)⊂**1**_2_·MTOA^+^ capsule. A 2D ROESY experiment allowed the assignment of the signals for the two methylene protons of the encapsulated **12**. The bound trimethylalkylammonium group moved upfield and showed intermolecular nOes with protons in the *meso*-phenyl protons (H^a^, H^b^) and the urea groups of the calix[4]pyrrole units. Altogether, these observations supported the co-encapsulation of **12** and chloride in **1**_2_. The *N*-methyl group of the MTOA co-bound cation resonated at *δ* = 0.34 ppm owing to its location in the base of the calix[4]pyrrole unit opposite to the encapsulated chloride. A DOSY NMR experiment performed on a CDCl_3_ solution of tetraurea **1**, betaine **12** and MTOACl in a 2 : 1 : 1 molar ratio provides a diffusion coefficient value in total agreement with that measured for the capsular assembly (**3**·Cl^–^)⊂**1**_2_·MTOA^+^ (Fig. S59†).‖
‖Gratifyingly, the combination of tetraurea **1**, betaine **12** and MTOABr in a 2 : 1 : 1 molar ratio also produces the formation of the dimeric capsular assembly (**12**·Br^–^)⊂**1**_2_·MTOA, in which the betaine and bromide are co-encapsulated. However, the higher packing coefficient (58%) for the complex reduces its stability and also the proton signals of the four-particle 1 : 1 : 1 complex **12**⊂**1**·MTOA^+^·Br^–^ can be observed as a minor species (ESI).


Substitution of MTOACl with TBACl produced the corresponding encapsulation assembly (**12**·Cl^–^)⊂**1**_2_·TBA^+^ to a minimum extent (Fig. S50†). The ^1^H NMR spectrum of the mixture displayed a set of intense signals assigned to ill-defined aggregates. This result reinforced the importance of the MTOA co-bound cation producing the initially formed Cl^–^⊂**1**·MTOA^+^ complex displaying a separated binding geometry and reducing the hydrogen-bonding competition of the chloride for the urea arms, which constituted a detrimental process for the assembly of capsular aggregates. As could be expected, an equimolar solution of betaine **12**, tetraurea **1** and MTOACl produced a ^1^H NMR spectrum showing sharp and well-resolved signals that were in agreement with the formation of the 1 : 1 : 1 inclusion complex **12**⊂**1**·MTOA^+^·Cl^–^ (Fig. S47†). The inclusion of the betaine **12** in the aromatic cavity of **1** was supported by the downfield shift of the pyrrolic NHs and the upfield signals observed for the protons of the bound guest. The signals for the trimethylalkylammonium group (Table 2) were significantly less upfield shifted than those for the capsular assembly (**12**·Cl^–^)⊂**1**_2_·MTOA^+^ and this is in agreement with the formation of the inclusion complex **12**⊂**1**·MTOA^+^·Cl^–^. The methylene protons alpha to the carboxylate and alpha to the ammonium group resonate less upfield than in the capsular assembly (Table 2). The placement of the MTOA cation at the shallow cavity of the calixpyrrole was evidenced by the typical upfield shift of the *N*-methyl protons. A DOSY NMR experiment performed on a CDCl_3_ solution of tetraurea **1**, betaine **12** and MTOACl in an equimolar ratio provides a diffusion coefficient value that was fully consistent with the formation of the **12**⊂**1**·MTOA^+^·Cl^–^ complex based on the previous values determined for related aggregates in this work (Fig. S60†).

All together, these results demonstrated that the self-assembly process of the chemical system constituted by betaine **12**, tetraurea **1** and MTOACl was also responsive to stoichiometric control.

*N*,*N*,*N*-Trimethyl glycine **13**, a.k.a. glycine betaine, has only one methylene carbon as a linker between its charged carboxylate and trimethylammonium groups. The calculated packing coefficient value for the capsular assembly (**13**·Cl^–^)⊂**1**_2_·MTOA^+^ was 53%, indicating a reduced size complementarity for the co-encapsulation of **13**, instead of **12**, with chloride. In agreement with this calculation, the ^1^H NMR spectrum of a solution containing a mixture of tetraurea **1**, betaine **13** and MTOACl in a 2 : 1 : 1 molar ratio was composed of signals for the capsular assembly (**13**·Cl^–^)⊂**1**_2_·MTOA^+^ and the inclusion complex **13**⊂**1**·MTOA^+^·Cl^–^. By integrating selected proton signals in each one of the two aggregates we determined that they were present in solution in a ratio close to 1 : 1.

**Fig. 5:**
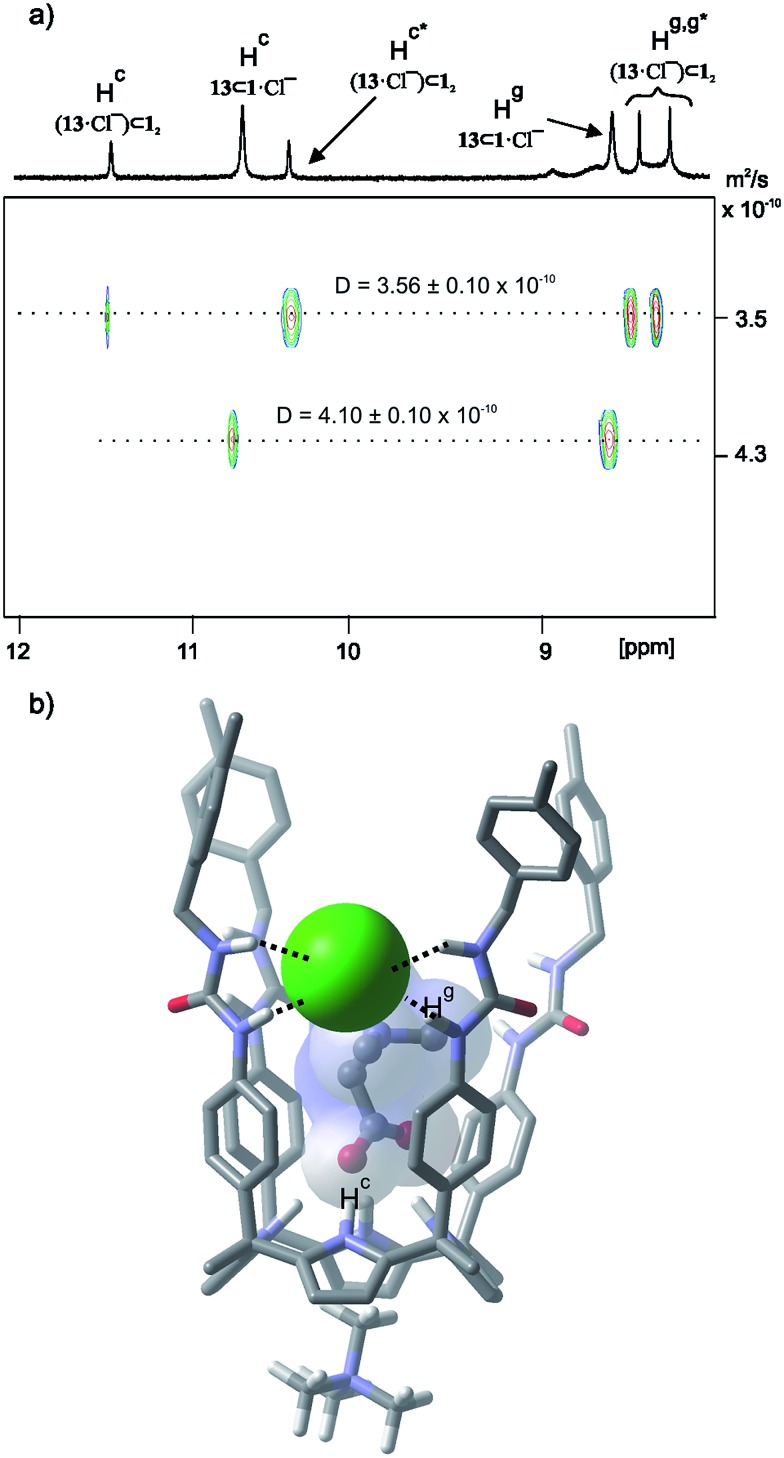
(a) ^1^H pseudo-2D DOSY plot of a CDCl_3_ solution of tetraurea **1**, betaine **13** and MTOACl in a 2 : 1 : 1 molar ratio. See Fig. 1 for the proton assignment. (b) Energy minimized (MM3) structure of the four-particle 1 : 1 : 1 assembly **13**⊂**1**·MTOA^+^·Cl^–^. Non-polar hydrogens are removed and the MTOA cation is depicted as TMA for clarity.

A DOSY NMR experiment performed on the mixture containing the capsular assembly (**13**·Cl^–^)⊂**1**_2_·MTOA^+^ and the 1 : 1 : 1 complex **13**⊂**1**·MTOA^+^·Cl^–^ evidenced the difference in size of the two aggregates (Fig. 5a). The lower diffusion constant value was in agreement with those determined for related capsular assemblies. The larger one coincided with that expected for a 1 : 1 : 1 inclusion complex. DOSY NMR allowed the undoubted assignment of the proton signals corresponding to each species. The methylene protons of bound **13** resonated at 0.70 ppm in the capsular assembly (**13**·Cl^–^)⊂**1**_2_·MTOA^+^ whereas those in the 1 : 1 : 1 inclusion complex **13**⊂**1**·MTOA^+^·Cl^–^ appeared less upfield shifted at *δ* = 0.84 ppm. The *N*-methyl protons of the MTOA cation resonated as a single signal at *δ* = 0.34 ppm. The resulting upfield shift indicated their placement at the shallow cavity of the calixpyrrole. The diffusion coefficient value calculated for the *N*-methyl cation represented the weighted average of the two species indicating that the cation was involved in an exchange process that was fast on the DOSY timescale. On the contrary, the chemical exchange between the calix[4]pyrrole units involved in the two species was slow on the EXSY timescale. The use of an equimolar mixture of the components produced the 1 : 1 : 1 complex as the exclusive species in solution (Fig. S53†).

## Conclusions

In summary, the reported findings emphasize the subtlety of the requisites for encapsulation to occur in supramolecular capsules stabilized by hydrogen bonding interactions. We conclude that the responsiveness of the stoichiometry of the complexes in response to the changes in the stoichiometry required (a) the use of a methyltrialkylammonium cation as the chloride counter-ion and (b) that the sum of volumes of the encapsulated guests was adequate to fill a little more than half of the capsule's interior. We have shown rare examples of the ordered encapsulation assemblies of multiple guests. The introduction of polar groups in the capsule interior and the establishment of directional interactions provided unprecedented ordered encapsulation complexes of multiple polar guests displaying high kinetic and thermodynamic stability.

## Supplementary Material

Supplementary information

Crystal structure data
