# Supplementary material for: Ordered co-encapsulation of chloride with polar neutral guests in a tetraurea calix[4]pyrrole dimeric capsule
Source: Chem Sci. 2015 Jul 20;6(11):6325–33. doi: 10.1039/c5sc02024g (PMC6054108; doi:10.1039/c5sc02024g)
Supplement: Supplementary file 1 [file SC-006-C5SC02024G-s001.pdf]

Electronic Supplementary Material (ESI) for Chemical Science.
This journal is © The Royal Society of Chemistry 2015

SUPPORTING INFORMATION

Ordered co-encapsulation of chloride with polar neutral guests in a tetraurea calix[4]pyrrole dimeric capsule

Albano Galán‡, Virginia Valderrey‡ and Pablo Ballester*‡§

‡ *Institute of Chemical Research of Catalonia (ICIQ), Avgda. Països Catalans, 16, 43007, Tarragona, Spain.*

§ *Catalan Institution for Research and Advanced Studies (ICREA), Passeig Lluís Companys, 23, 08018, Barcelona, Spain.*

pballester@iciq.es

Table of Contents

Experimental details S2

NMR experiments of tetraurea **1**, pyridine *N*-oxide **5** and ion-pairs. S3

^{1}H NMR titrations and ITC experiments with the model systems S15

NMR experiments of tetraurea **1**, trimethylamine *N*-oxide **3** and ion-pairs. S27

NMR experiments of tetraurea **1**, betaines and ion-pairs. S39

DOSY NMR experiments S52

Energy minimized structures and packing coefficients S64

Experimental details

Tetraurea calixpyrrole **1**[1], calixpyrrole model **10**[2] , urea model **9**[3] and betaine **12**[4] were prepared according to known procedures. Other chemicals and solvents were purchased from Aldrich and used without further purification.

^{1}H NMR spectra were recorded on a Bruker Avance 400 (400.1 MHz for ^{1}H-NMR) and Bruker Avance 500 (500.1 MHz for ^{1}H-NMR) ultrashield spectrometer. The deuterated solvents (Aldrich) used are indicated in the experimental part; chemical shifts are given in ppm, relative to residual solvent.

DOSY NMR experiments were performed using precision Micro NMR tubes from New Era Enterprises (Ref NE-H5/2.5) for the solutions containing the supramolecular assemblies. Regular 5 mm NMR tubes were used for the DOSY experiments of the free guests. In all cases, the spectra were acquired at 298K using a big delta (d20) = 150 ms and a little delta (p30) = 1000 µs on a Bruker Avance 500 MHz equipped with a cryoprobe. The fit of the decay data was achieved using the Dynamics Center software, version 2.3, provided by Bruker BioSpin GmbH.

---

[1] P. Ballester and G. Gil-Ramirez, *Proc. Natl. Acad. Sci. U. S. A.*, 2009, **106**, 10455-10459.
[2] G. Gil-Ramirez, E. C. Escudero-Adan, J. Benet-Buchholz and P. Ballester, *Angew. Chem., Int. Ed.*, 2008, **47**, 4114-4118.
[3] W. Zhang, C. H. T. Chen and T. Nagashima, *Tetrahedron Lett.*, 2003, **44**, 2065-2068.
[4] Y. Chevalier and P. Leperchec, *J. Phys. Chem.*, 1990, **94**, 1768-1774.

NMR experiments of tetraurea 1, pyridine *N*-oxide 5 and ion-pairs.

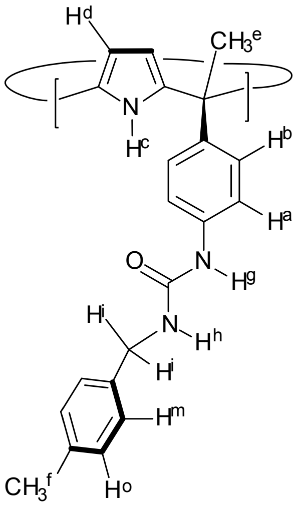

1

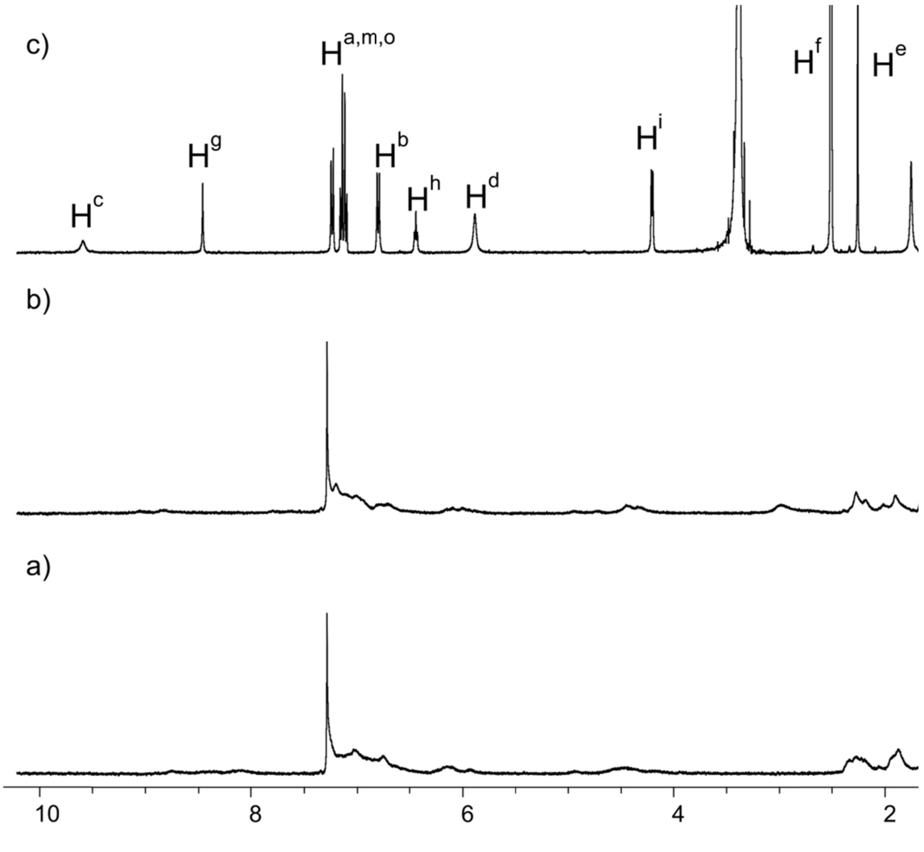

Figure S1. ^{1}H NMR spectra of a) d-chloroform suspension of tetraurea **1** b) d-chloroform solution of tetraurea **1** and 1.2 equivalents of MTOACl c) d_6-DMSO solution of tetraurea **1**.

In non-polar solvents the tetraurea derivative aggregates and produces ill-defined spectra (Figure S1a). However in polar solvents, like dimethylsulphoxide, the ^{1}H NMR of **1** shows sharp and well-defined signals (Figure S1c). The tetraurea derivative **1** does not produce a single and well-defined species upon the addition of 1.2 equivalents of methyltrioctylammonium chloride (Figure S1b).

The addition of 1 equivalent of pyridine *N*-oxide **5** to a d-chloroform solution of **1** produces the precipitation of the urea and the NMR spectrum shows no proton signals for **1** or **5** (See spectrum in Figure S9).

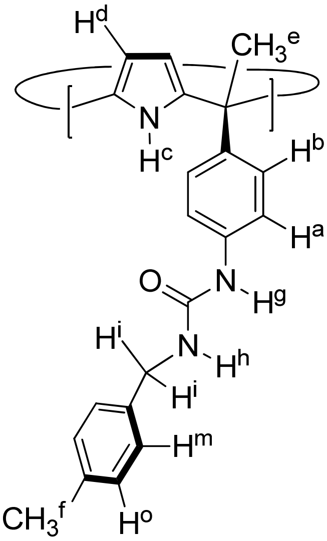

1

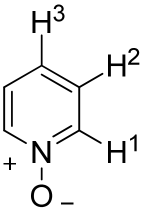

5

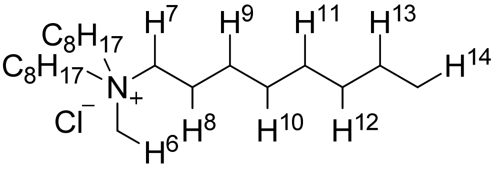

6

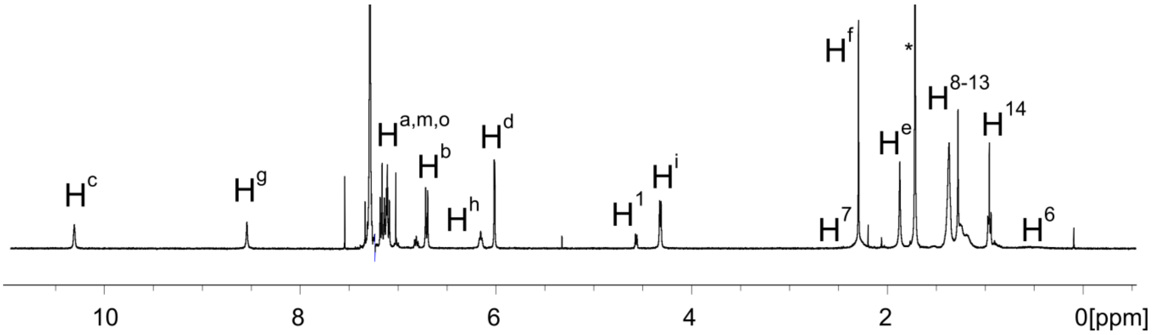

Figure S2. ^{1}H NMR spectrum in d-chloroform of an equimolar mixture of tetraurea **1**, pyridine *N*-oxide **5** and MTOACl **6**. *Residual water.

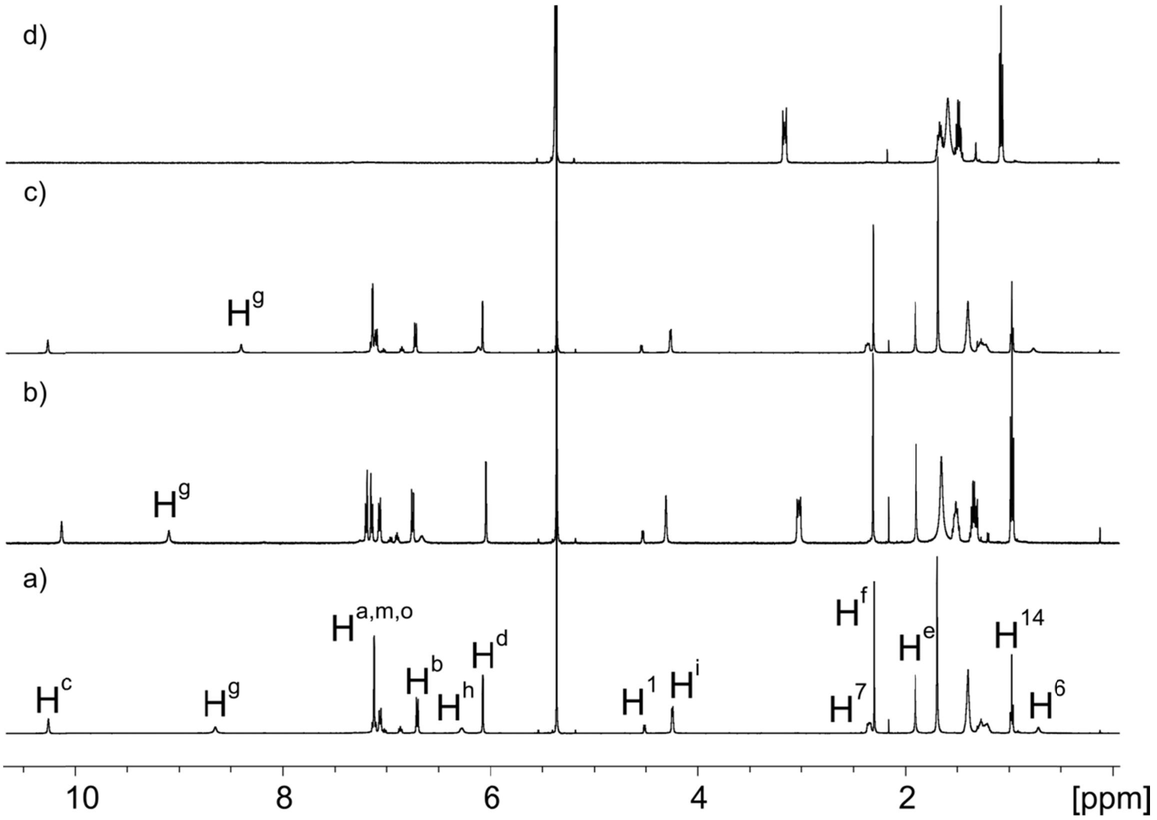

Figure S3. ^{1}H NMR spectra in d_2-dichloromethane of an equimolar mixture of tetraurea **1**, pyridine *N*-oxide **5** and a) MTOACl b) TBACl c) MTOABr d) PF_6TBA. See Figure S2 for proton assignment.

The methylene protons *alpha* to the nitrogen of the TBA cation (δ = 3.0 ppm, Figure S3b) are barely shifted when compared to the chemical shift of free TBACl. On the contrary, the methylene protons of the MTOA cation (H^7) resonate upfield shifted at 2.3 ppm. This evidences the better fit of the MTOA cation in the shallow cavity of the calixpyrrole.

The urea NH protons are downfield shifted following the trend TBACl >> MTOACl > MTOABr. The hydrogen bonding between chloride and the urea NHs is stronger than when bromide is the anion resulting in an increased downfield shifting when chloride is involved.

The addition of a non-coordinating anion (PF_6TBA) does not break the urea aggregation to form the 1:1:1 complex and the complex pyridine *N*-oxide and tetraurea calixpyrrole remains insoluble. Only proton signals of the TBA cation are observable.

The ^{1}H NMR of the 1:1:1 complex is formed both in d-chloroform and d_2-dichloromethane (Figure S2 and Figure S3a respectively). Both NMR spectra display no appreciable differences. We used d_2-dichloromethane when possible to show the aromatic proton region without the interference of the residual solvent peak.

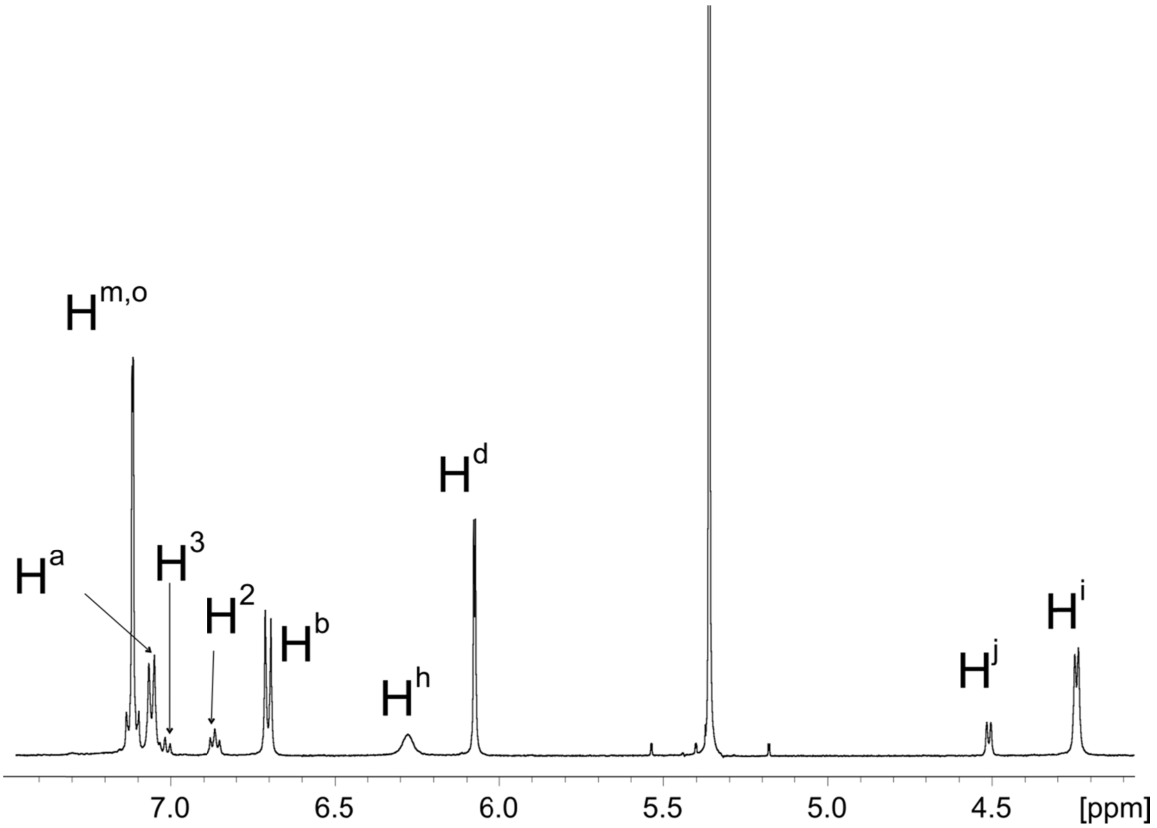

Figure S4. Selected region of the aromatic region of a ^{1}H NMR spectrum in d_2-dichloromethane of an equimolar mixture of tetraurea **1**, pyridine *N*-oxide **5** and MTOACl **6**. See Figure S2 for proton assignment.

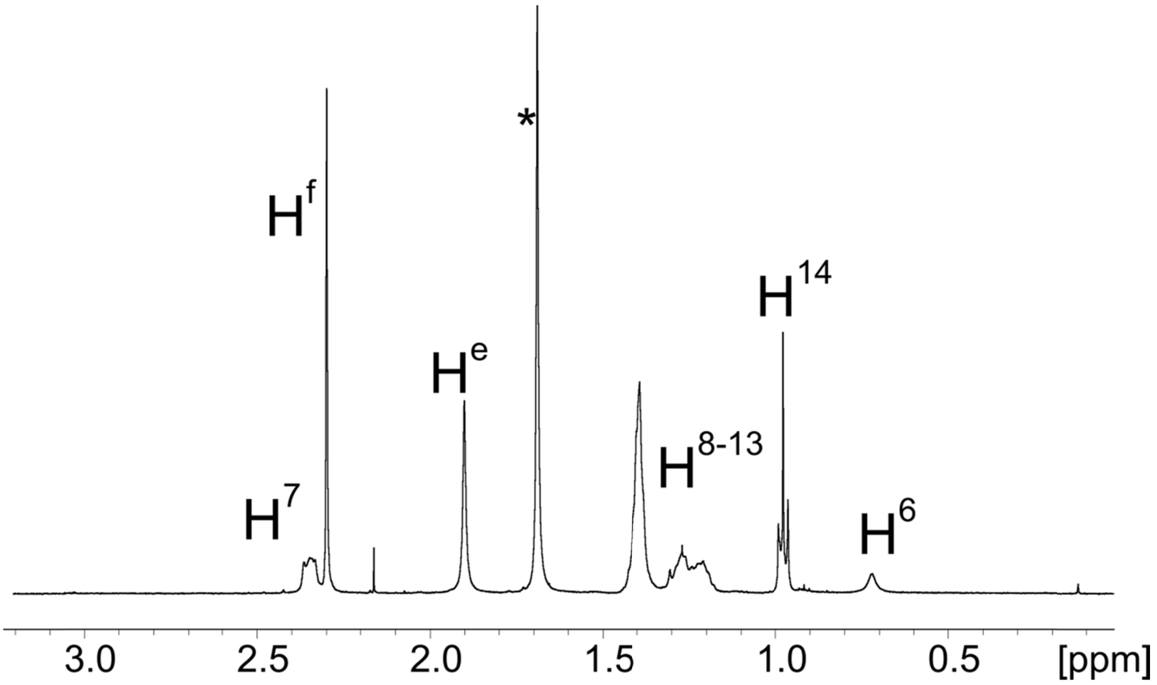

Figure S5. Selected region of the upfield region of a ^{1}H NMR spectrum in d_2-dichloromethane of an equimolar mixture of tetraurea **1**, pyridine *N*-oxide **5** and MTOACl **6**. See Figure S2 for proton assignment. *Residual water.

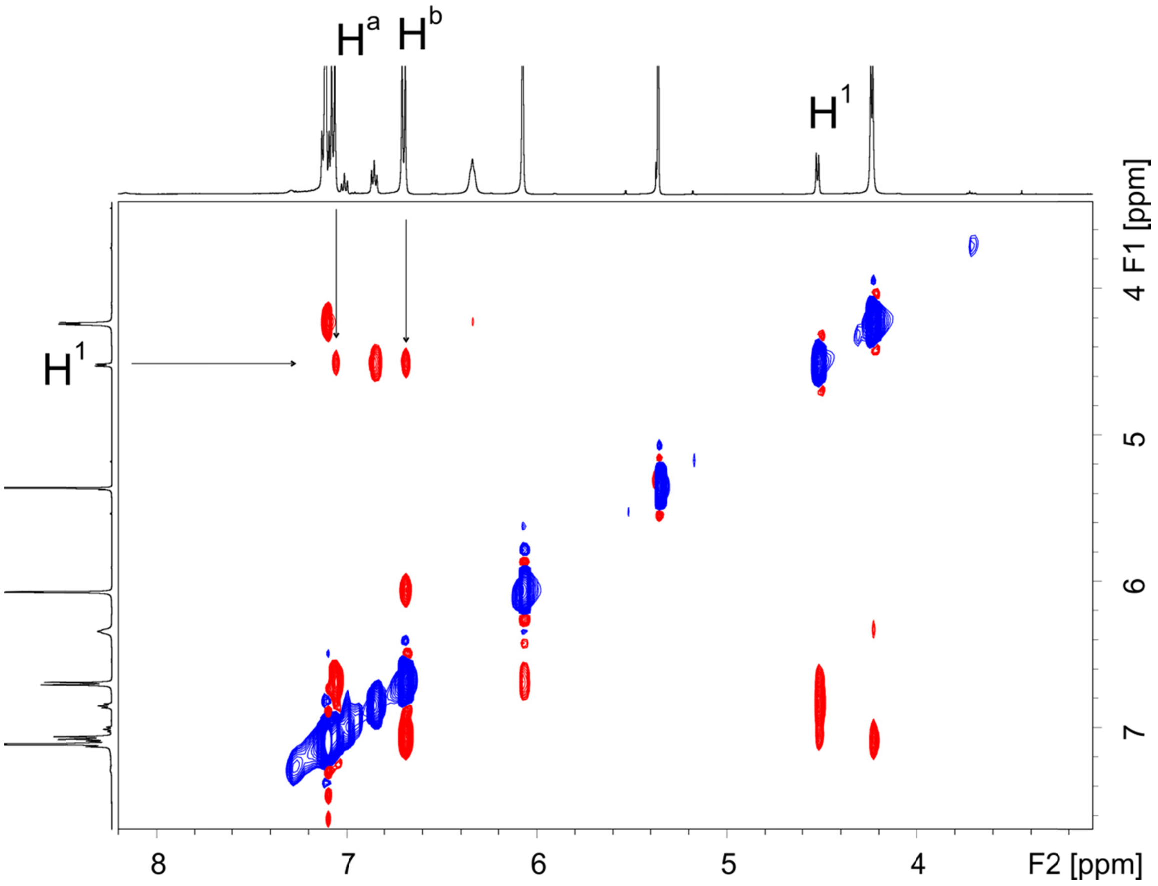

Figure S6. ROESY experiment of a d_2-dichloromethane equimolar solution of tetraurea **1**, pyridine *N*-oxide **5** and MTOACl showing cross-peaks indicating close-contact between the included pyridine protons and the aromatic walls of **1**. Mixing time (d8 = 300 ms).

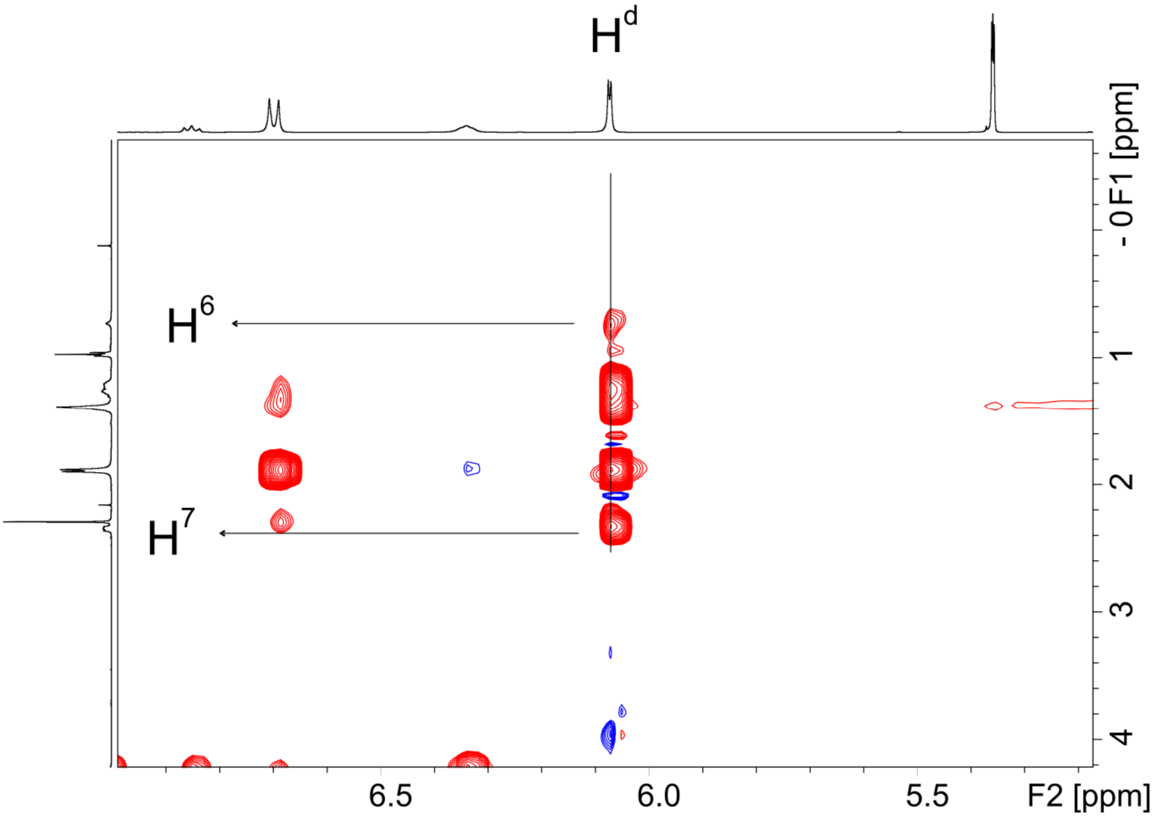

Figure S7. ROESY experiment of a d_2-dichloromethane equimolar solution of tetraurea **1**, pyridine *N*-oxide **5** and MTOACl showing cross-peaks indicating close-contact between the *beta*-pyrrolic protons of **1** and the cation protons evidencing the location of the cation in the shallow cavity of the calixpyrrole. Mixing time (d8 = 300 ms).

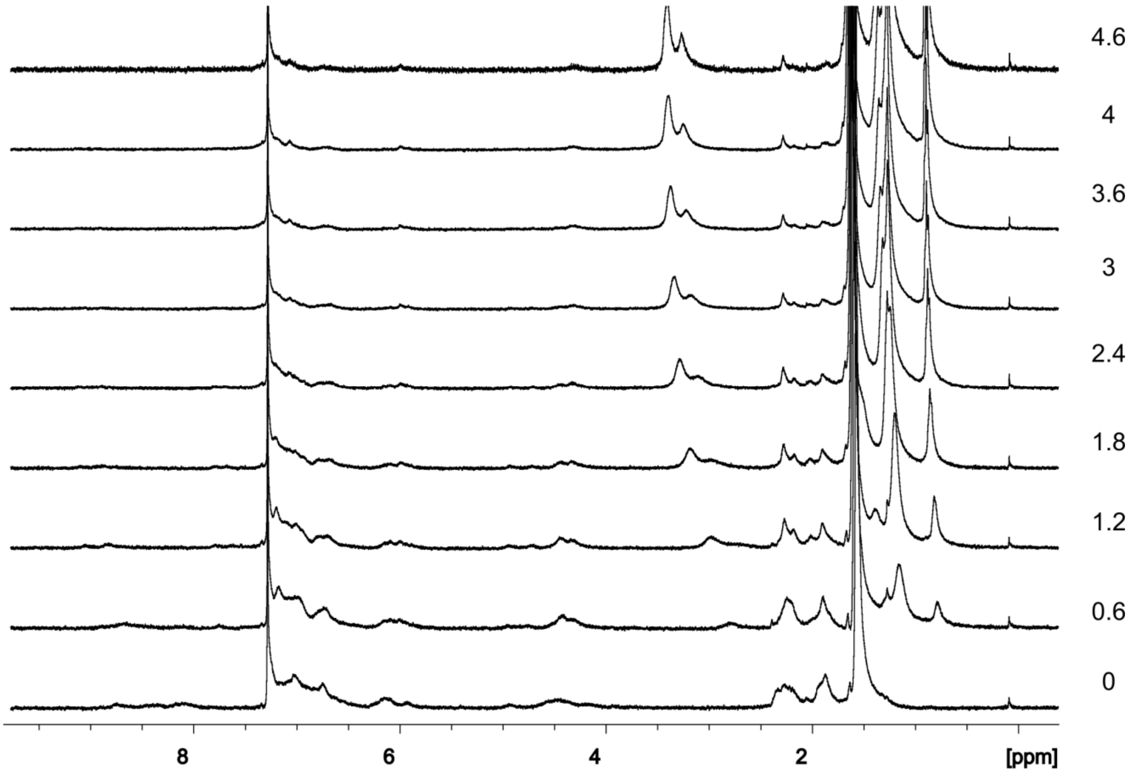

Figure S8. ^{1}H NMR titration of a d-chloroform solution of tetraurea **1** with increasing amounts of MTOACl. The number of equivalents of salt added is at the right margin.

The ion-pair does not break the aggregation between the urea groups to form discrete species even with an excess of salt.

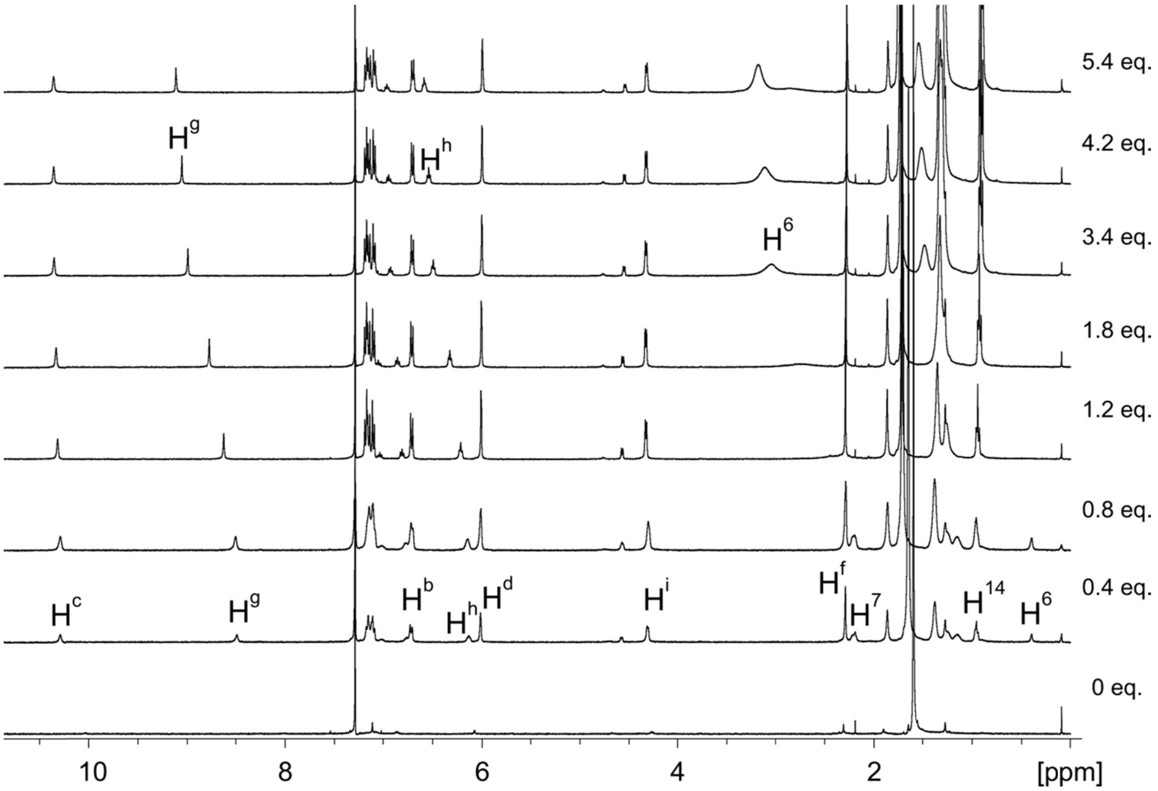

Figure S9. ^{1}H NMR titration of a 1.5 mM d-chloroform equimolar solution of tetraurea calixpyrrole **1** and pyridine *N*-oxide with methyltrioctylammonium chloride (MTOACl). See Figure S2 for proton assignment.

The 1:1 equimolar mixture of tetraurea aryl-extended calix[4]pyrrole and pyridine *N*-oxide is completely insoluble. Then, the addition of methyltrioctylammonium chloride dissolves the mixture allowing the observation of the four particle assembly **5**$\subset$**1**·MTOA·Cl^{-} complex. The exchange between free and bound MTOA cation is fast on the NMR time scale when more than one equivalent of MTOA is present. Upon increasing amounts of chloride, the proton signals for the ureas NHs (H^g and H^h) shift downfield progressively.

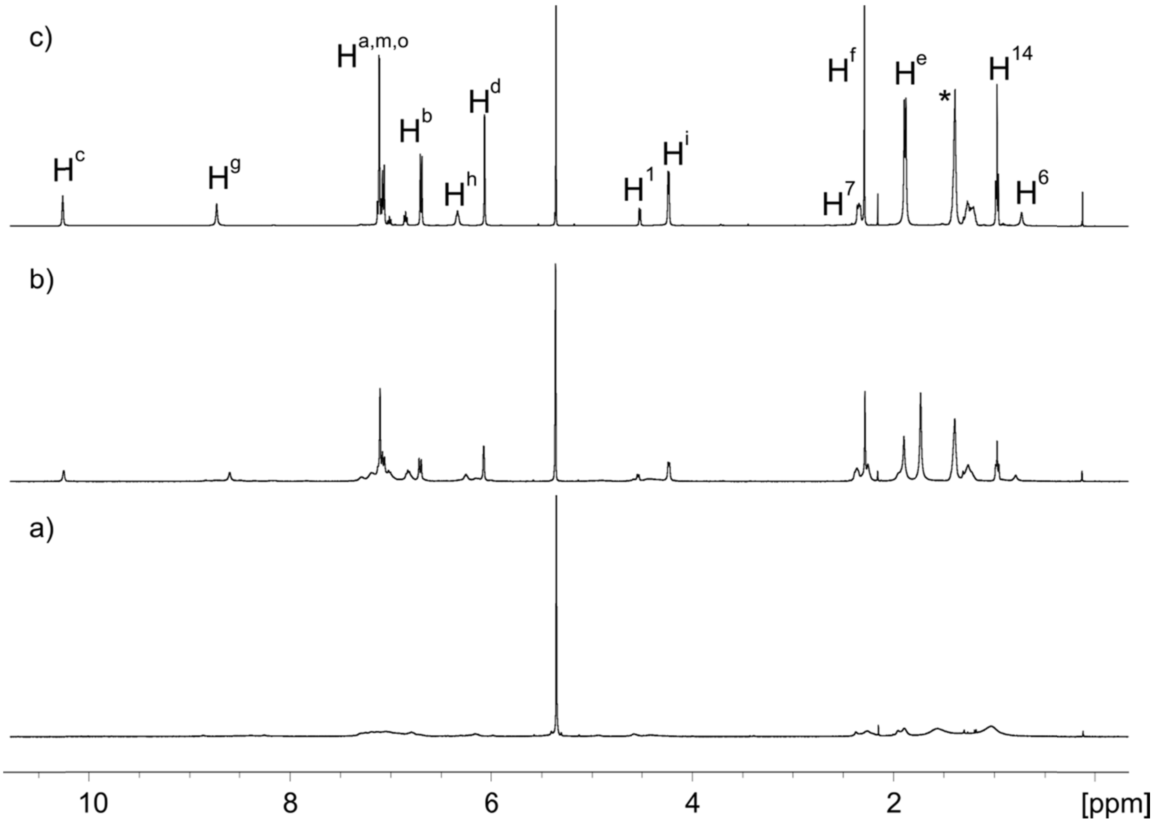

Figure S10. ^{1}H NMR spectra of a d_2-dichloromethane solution of a) free tetraurea and a mixture of tetraurea **1**, pyridine *N*-oxide and MTOACl in a b) 2:1:1 molar ratio c) 1:1:1 molar ratio. See Figure S2 for proton assignment. *Residual water.

The addition of 0.5 equivalents of pyridine *N*-oxide and 0.5 equivalents of MTOACl to a tetraurea solution (Figure S10b) produces a mixture of the four particle complex **5**⊂**1**·MTOA·Cl$^-$ (Figure S10c) and ill-defined aggregates.

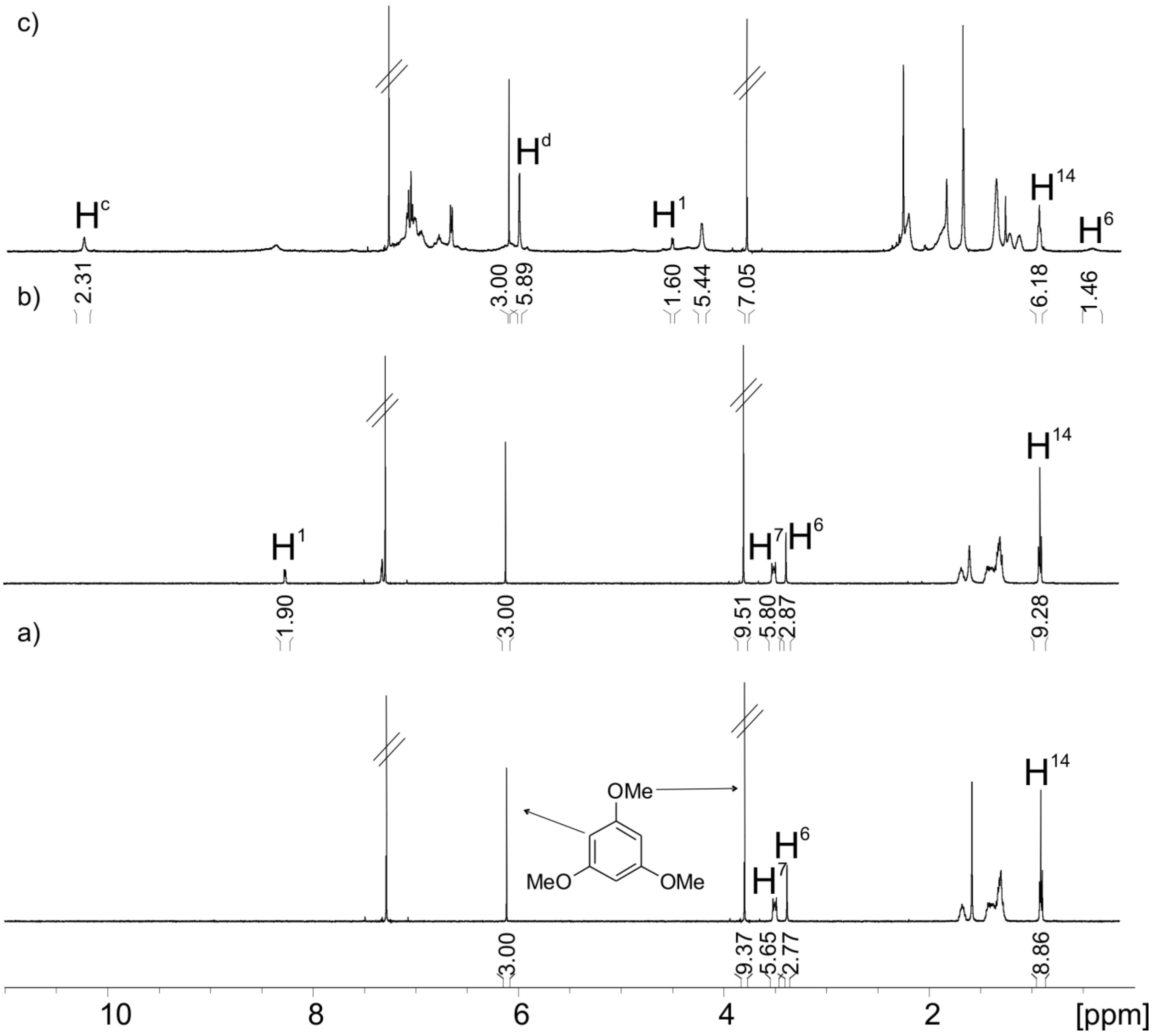

Figure S11. a) ^{1}H NMR spectrum of a d-chloroform solution of an equimolar mixture of 1,3,5-trimethoxybenzene (internal standard) and MTOACl b) addition of 1 equivalent of pyridine *N*-oxide to the previous mixture and c) addition of 0.5 equivalent of tetraurea **1** to the previous mixture. Integrals of selected peaks are displayed below the proton peak. See Figure S2 for proton assignment.

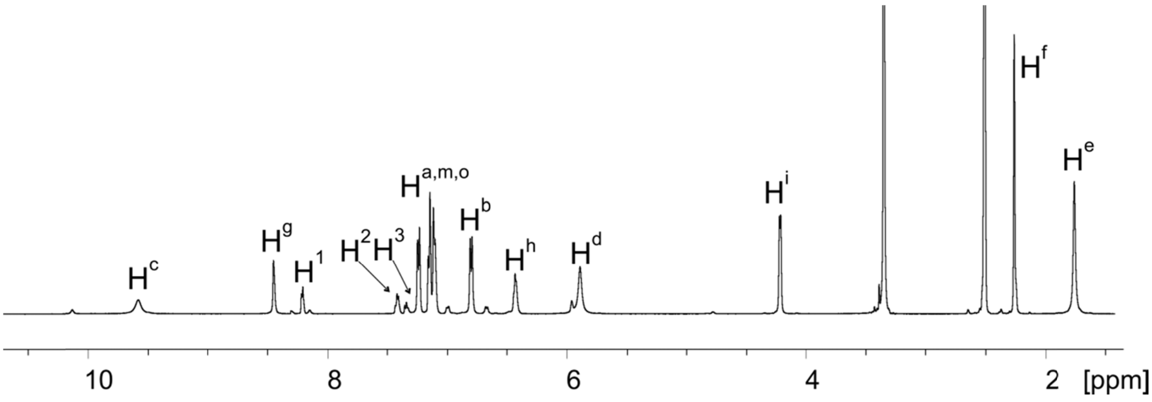

Figure S12. ^{1}H NMR spectrum in d_6-DMSO of the precipitate formed upon addition of pyridine *N*-oxide to a tetraurea **1** chloroform suspension. We observe free pyridine *N*-oxide and tetraurea **1**. See Figure S2 for proton assignment.

^{1}H NMR titrations and ITC experiments with the model systems

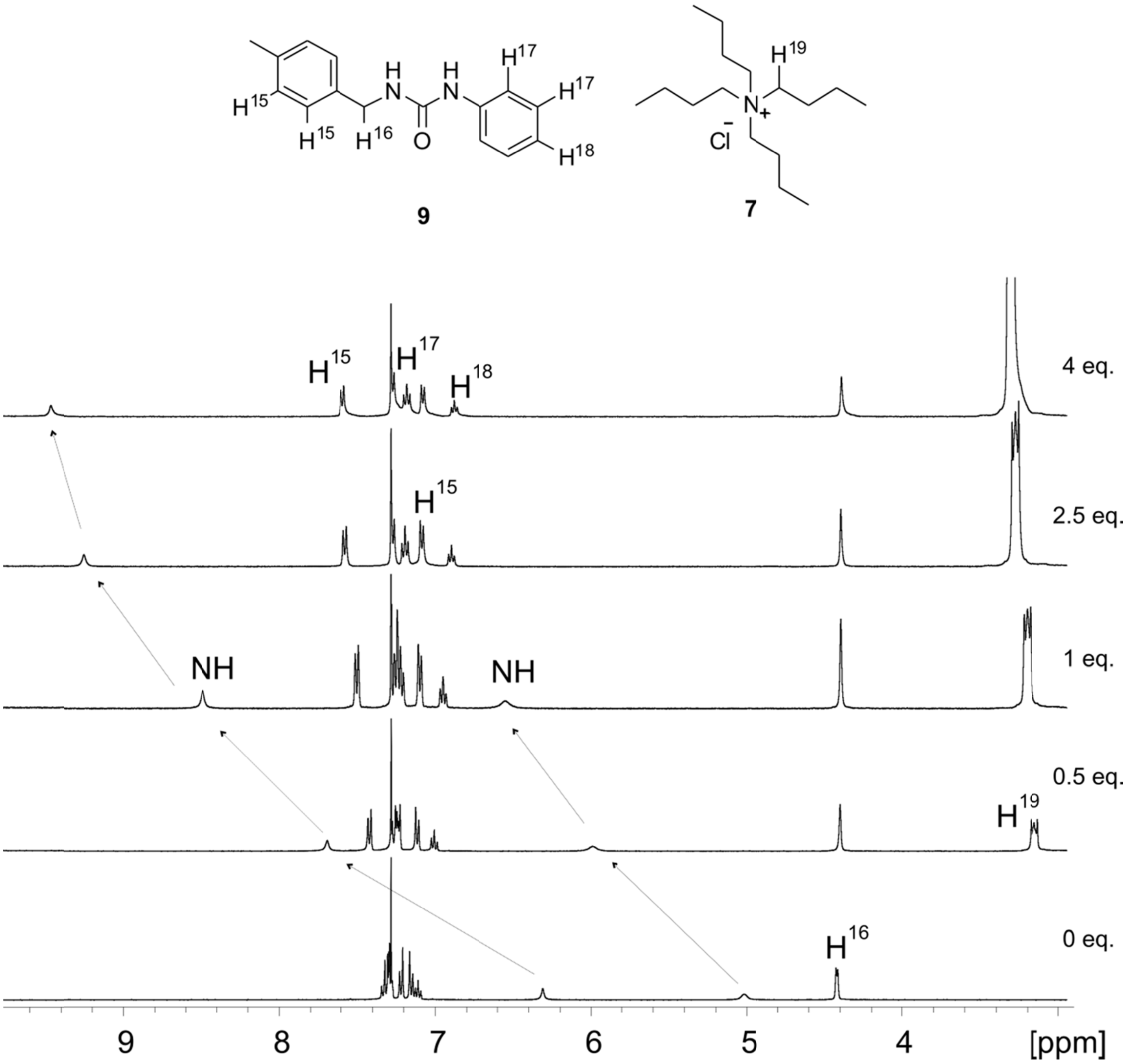

Figure S13. ^{1}H NMR titration of an 8 mM d-chloroform solution of urea model **9** with tetrabutylammonium chloride (TBACl).

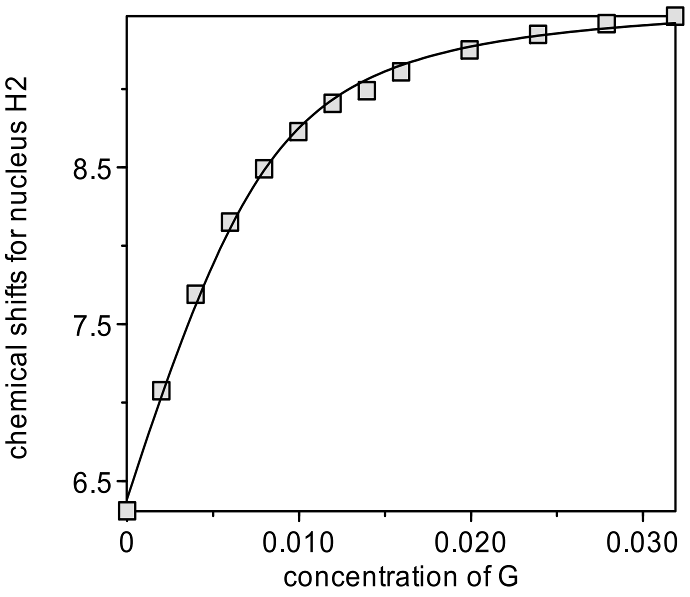

Figure S14. Fit of the chemical shift changes, experienced by the signals of NH protons of **9** during the titration with TBACl, using a 1:1 binding model (line) implemented in the HypNMR2008 software.

The association constant obtained by the fit of the chemical shift changes was:

$$K_{9\bullet7} = 797 \pm 131\ M^{-1}$$

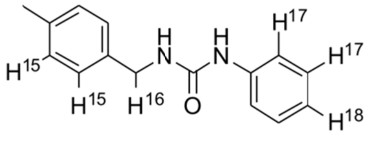

9

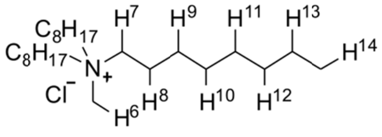

6

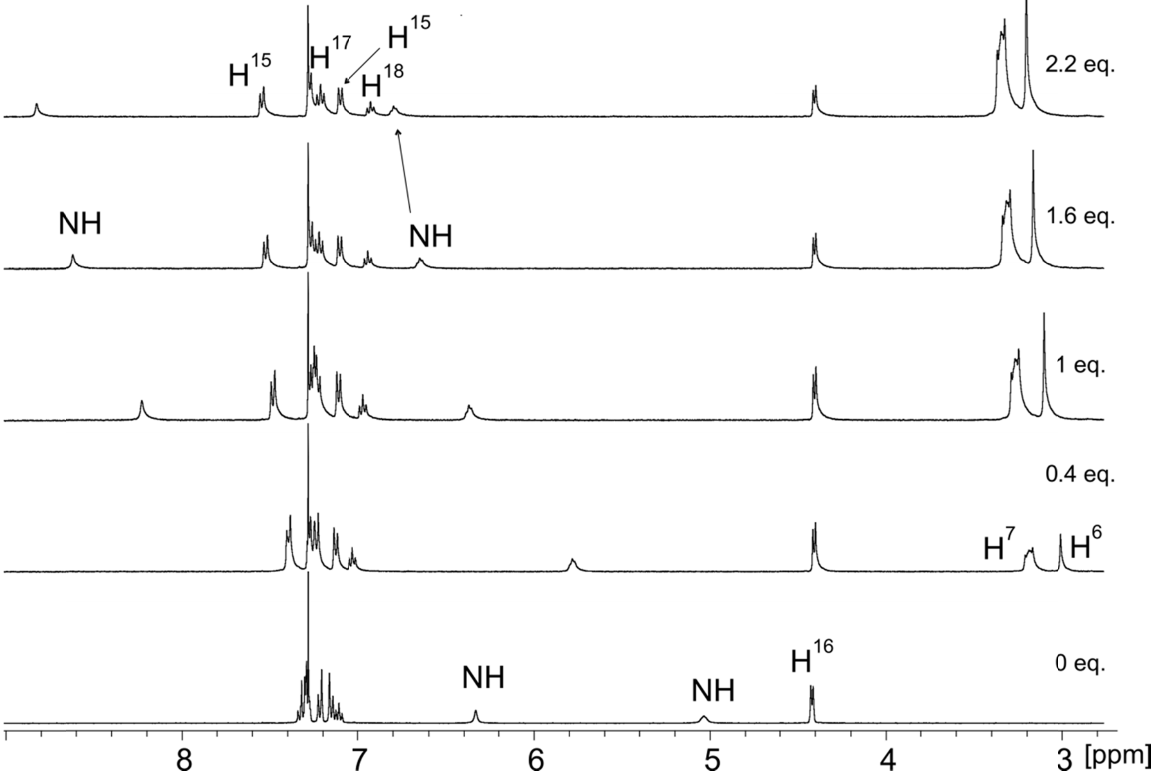

Figure S15. ^{1}H NMR titration of a 9 mM d-chloroform solution of urea model **9** with methyltrioctylammonium chloride (MTOACl).

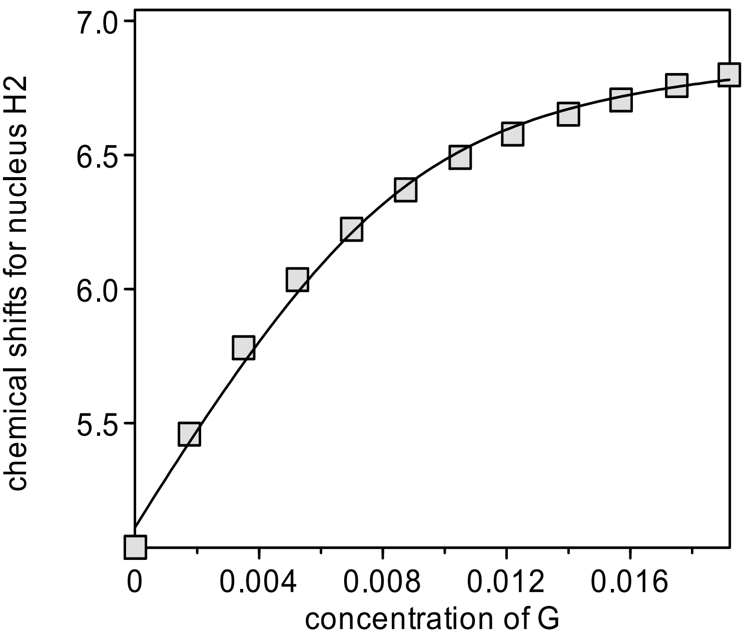

Figure S16. Fit of the chemical shift changes, experienced by the signals of NH protons of **9** during the titration with MTOACl, using a simple 1:1 binding model (line) and the HypNMR2008 software.

The association constant obtained by the fit of the chemical shift changes was:

$$K_{9\bullet 6} = 710 \pm 128\ M^{-1}$$

A $CDCl_3$ solution of urea **9** was titrated with increasing amounts of a stock solution of MTOACl (Figure S15) or TBACl (Figure S13). In both cases the urea NHs are gradually downfield shifted, as a result of the hydrogen-bonding. The formation of the complex presents fast exchange kinetics on the NMR time scale. The mathematical analysis of the NMR data fitted to a 1:1 binding model with association constant of ca. $K = 800\ M^{-1}$ for the formation of both complexes **9·7** and **9·6**. These data reveals that the interaction between a urea group and an ion-pair is practically not cation-dependent.

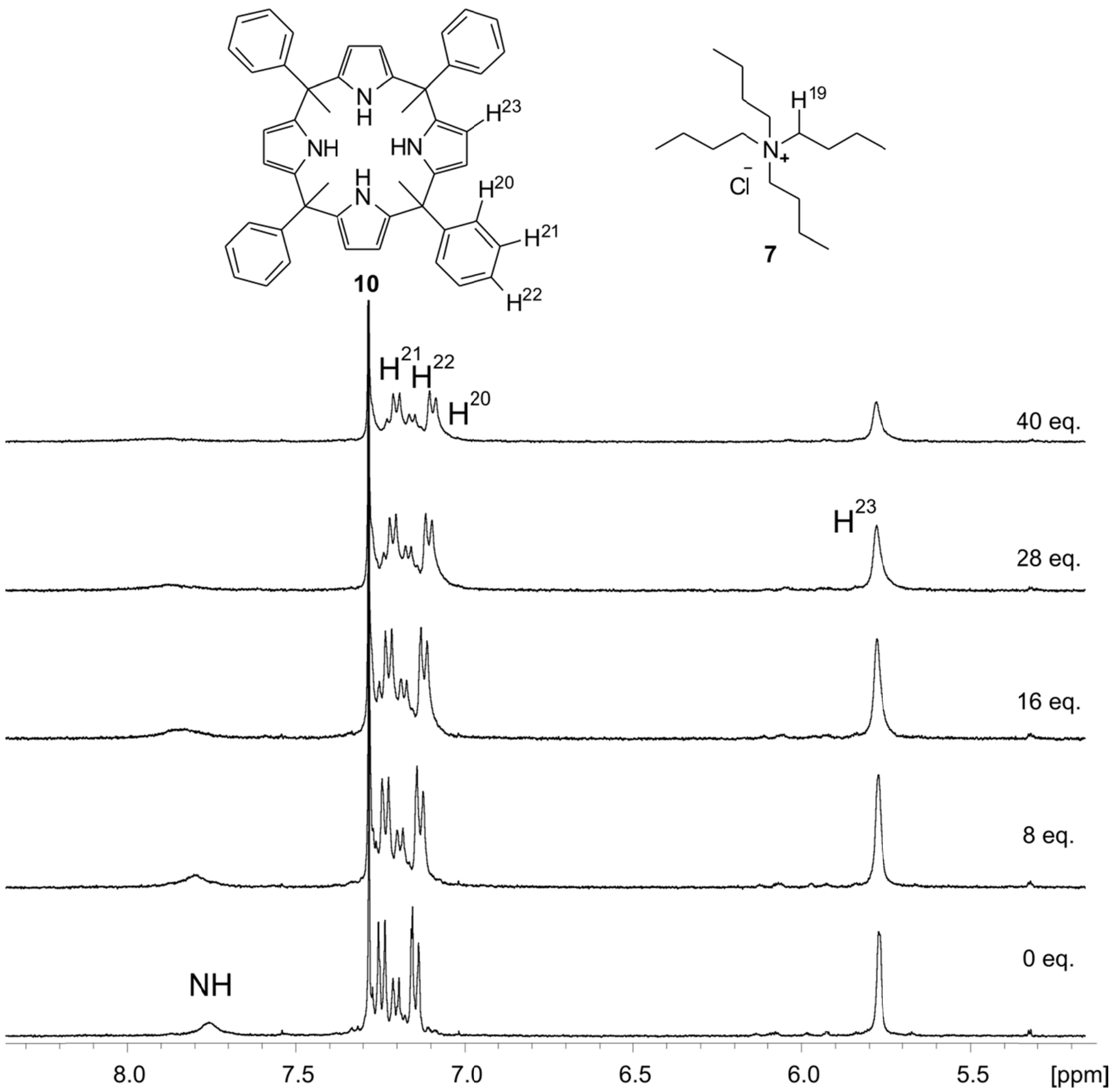

Figure S17. ^{1}H NMR titration of a 2.5 mM d-chloroform solution of tetraphenyl calixpyrrole model **10** with tetrabutylammonium chloride (TBACl).

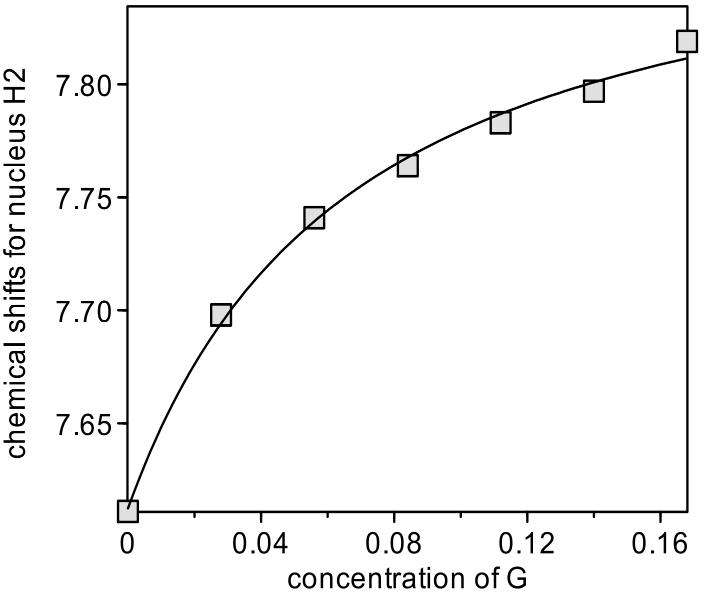

Figure S18. Fit of the chemical shift changes, experienced by the signals of NH protons of **10** during the titration with TBACl, using a 1:1 binding model (line) implemented in the HypNMR2008 software.

The association constant obtained by the fit of the chemical shift changes was:

$$K_{10\bullet7} = 17.0 \pm 1.4\ \mathrm{M}^{-1}$$

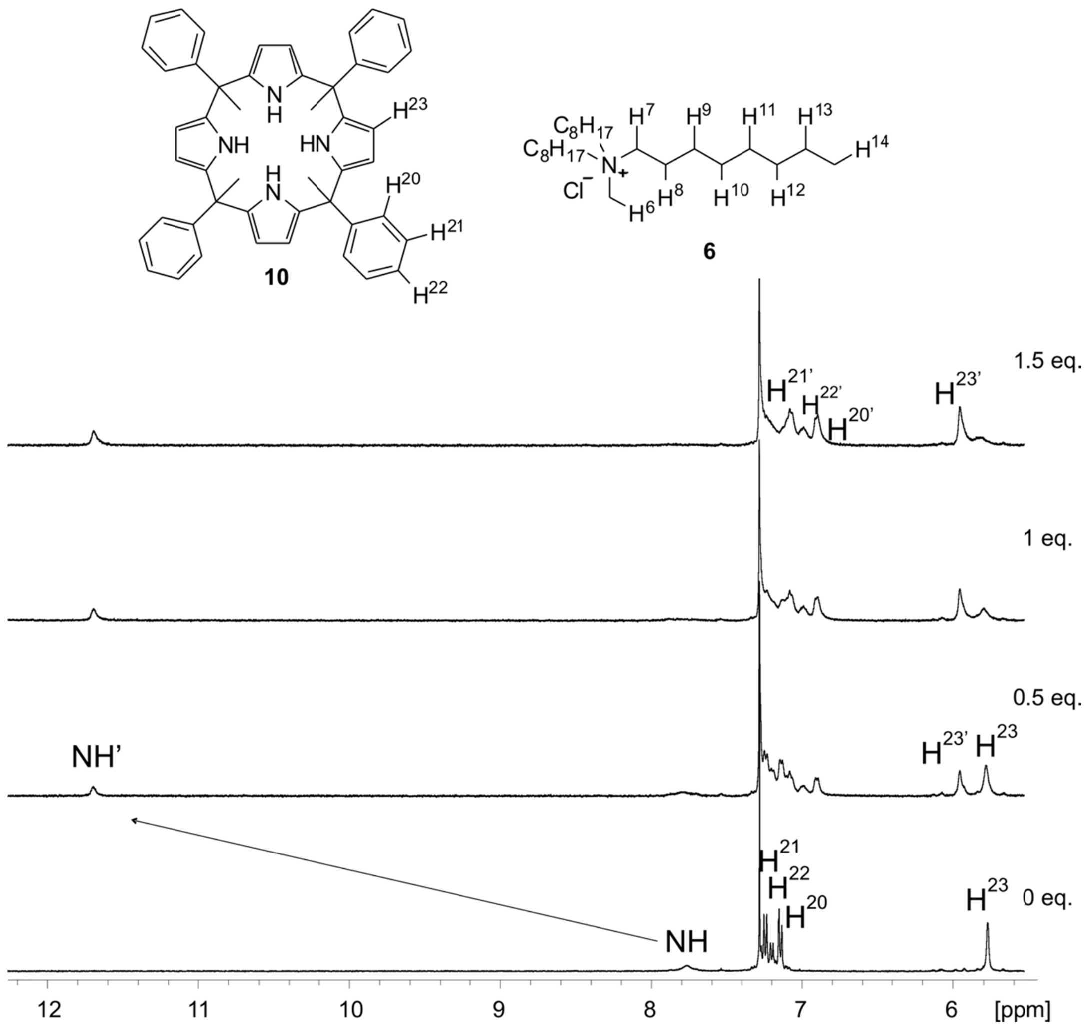

Figure S19. ^{1}H NMR titration of a 2 mM d-chloroform solution of tetraphenyl calixpyrrole model **10** with methyltrioctylammonium chloride (MTOACl). Primed numbers indicate signals of protons bound.

A $CDCl_3$ solution of calixpyrrole **10** was titrated with increasing amounts of MTOACl (Figure S19). We observe different set of proton signals for the free and the bound host evidencing a binding process that is slow on the NMR time scale. Addition of 1 equivalent of salt to the calixpyrrole solution does not result in full complexation of the host indicating an association constant lower than 10^4 M^{-1}. Direct integration of the NMR spectra to estimate the association constant proves to be difficult. Thus, we performed isothermal titration calorimetry (ITC) experiments (vide infra). The analogous titration of calixpyrrole **10** with TBACl (Figure S17) shows a fast exchange regime on the NMR time scale and an association constant of only ca. K = 20 M^{-1} as estimated by the fit of the NMR chemical shifts (Figure S18). This drop on the magnitude of the binding constant highlights the importance of the cation in the ion-pair recognition by the calixpyrrole core. Previous studies showed that unlike the TBA cation, MTOA cation nicely fits in the shallow cup of

the calixpyrrole.[5] This translates into higher association constants in the recognition of ion-pairs by calixpyrroles when MTOA is used as countercation instead of TBA.

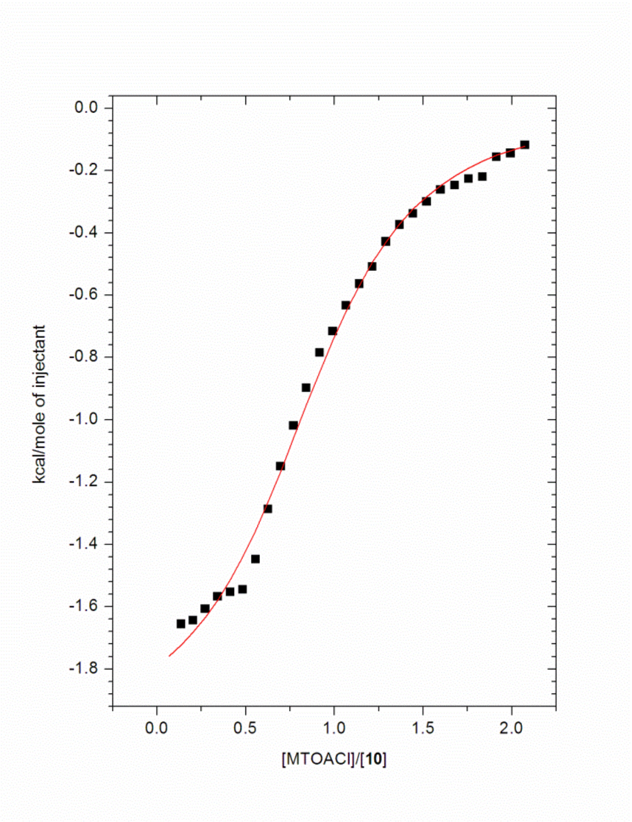

Figure S20. Normalized integration heat *vs* molar ratio obtained in the ITC experiment of a chloroform solution of tetraphenyl calixpyrrole **10** with MTOACl. The continuous red line represents the least-squares-fit of the data to a one set of sites binding model.

The ITC data fitted to a theoretical isotherm considering a 1:1 binding model by using Microcal ITC data analysis software. The calculated thermodynamic variables derived from the fit were: $K_{\mathbf{6\subset10}} = 2.8 \times 10^3 \pm 1.2 \times 10^3$ M^1, n = 0.93, ΔH = -2 kcal/mol.

[5] V. Valderrey, E. C. Escudero-Adan and P. Ballester, *Angew. Chem., Int. Ed.*, 2013, **52**, 6898-6902.

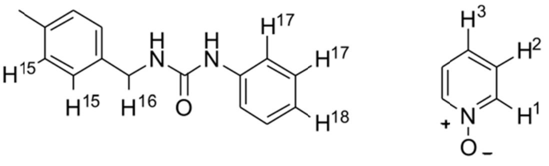

9 **5**

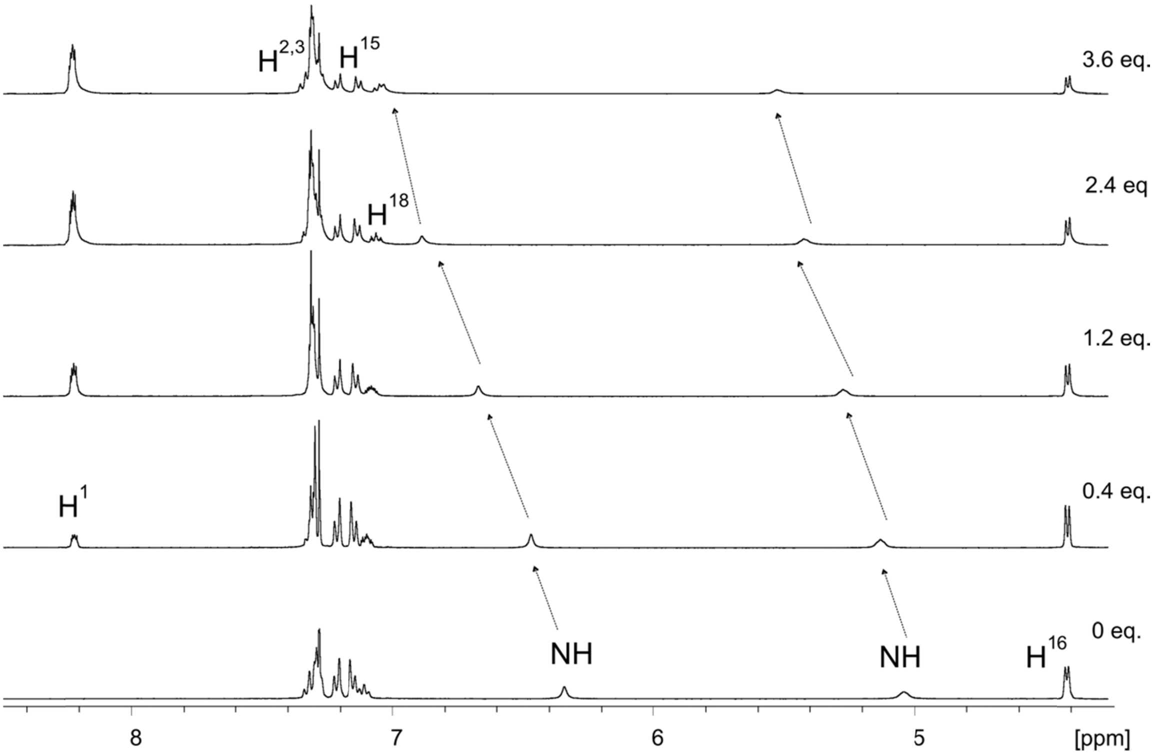

Figure S21. ^{1}H NMR titration of a 10 mM d-chloroform solution of model urea **9** with pyridine *N*-oxide.

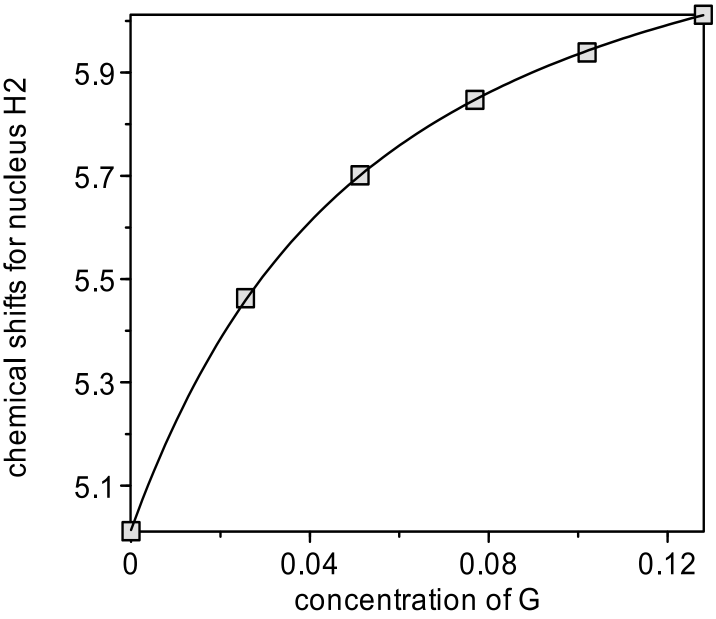

Figure S22. Fit of the chemical shift changes, experienced by the signals of NH protons of **9** during the titration with pyridine *N*-oxide, using a 1:1 binding model (line) implemented in the HypNMR2008 software.

The association constant obtained by the fit of the chemical shift changes was:

$$K_{9\bullet 5} = 28.6 \pm 9.0 \text{ M}^{-1}$$

A $CDCl_3$ solution of urea **9** was titrated with increasing amounts of a stock solution of pyridine *N*-oxide **3** (Figure S21). The urea NHs are gradually downfield shifted but large amounts of guest needs to be added to appreciate the shifting, revealing a low binding constant. The binding process shows fast kinetics in the NMR time scale and the data fitted to a 1:1 binding model with an association constant for the formation of the complex **9·5** of ca. 30 M^{-1}.

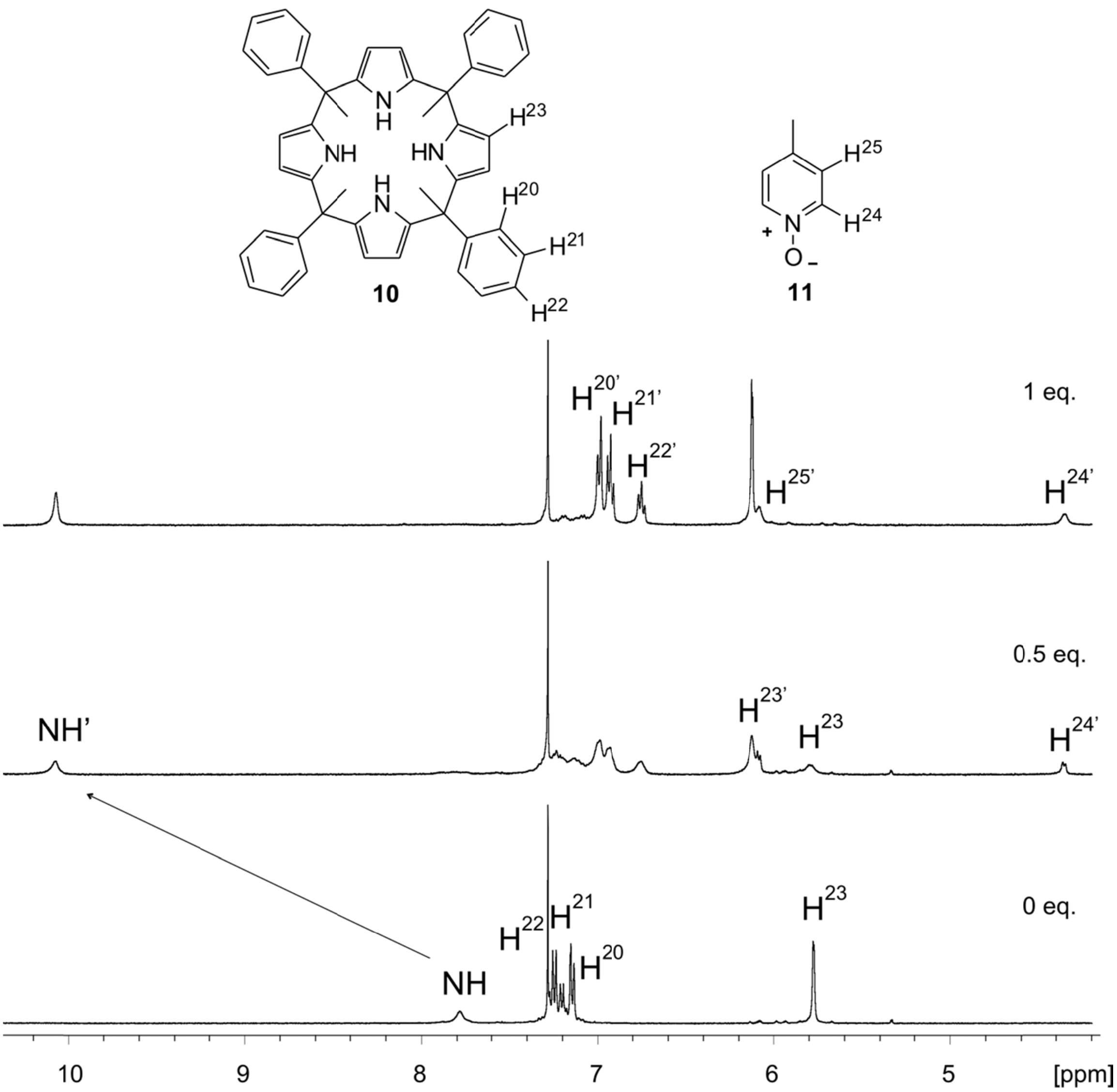

Figure S23. ^{1}H NMR titration of a 3 mM d-chloroform solution of model tetraphenylcalixpyrrole **10** with 4-methylpyridine *N*-oxide.

The binding constant for the calixpyrrole-pyridine *N*-oxide interaction could not be measured using **10** and **3** due to the poor solubility of the resulting complex in chloroform. Instead, we decided to use the 4-methyl substituted pyridine *N*-oxide **11** for this purpose (Figure S23). Addition of *N*-oxide **11** to a $CDCl_3$ solution of calixpyrrole **10** produces separated set of proton signals for the bound and free guest. In consequence, the exchange regime for this binding process is slow on the NMR time scale. Addition of 1 equivalent of **11** to a $CDCl_3$ solution of calixpyrrole **10** produces the full complexation of the host. Thus, the association constant for the binding process is higher than 10^4 M^{-1} and cannot be accurately measured by ^{1}H NMR.

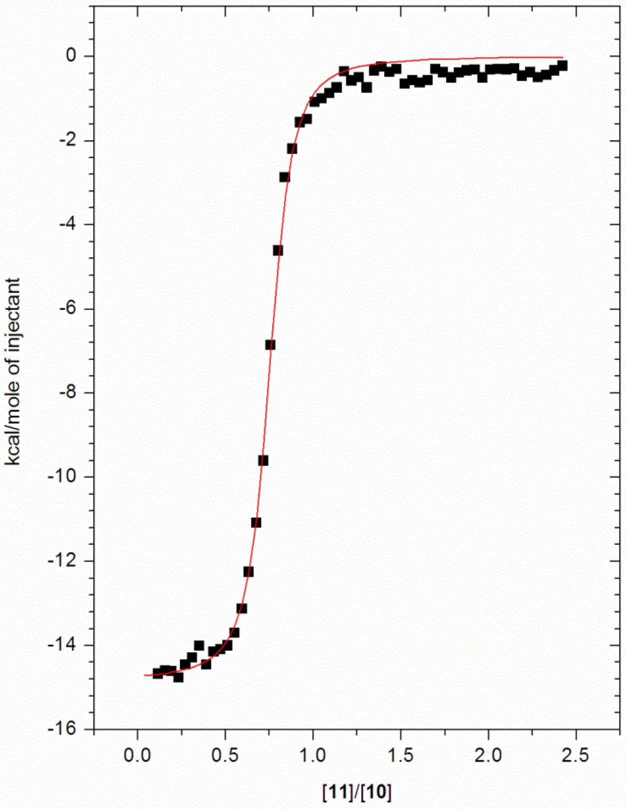

Figure S24. Normalized integration heat *vs* molar ratio obtained in the ITC experiment of a chloroform solution of tetraphenyl calixpyrrole **10** with 4-methylpyridine *N*-oxide. Fit to the theoretical binding isotherm (red line) using a 1:1 binding model. The mathematical analysis of the titration data was performed using the Origin software.

The ITC data fitted to a theoretical isotherm considering a 1:1 binding model by using Microcal ITC data analysis software. The calculated thermodynamic variables derived from the fit were: $K = 1.0 \times 10^6 \pm 0.2 \times 10^6\ M^1$, n = 0.80, ΔH = -14 kcal/mol.

NMR experiments of tetraurea 1, trimethylamine *N*-oxide 3 and ion-pairs.

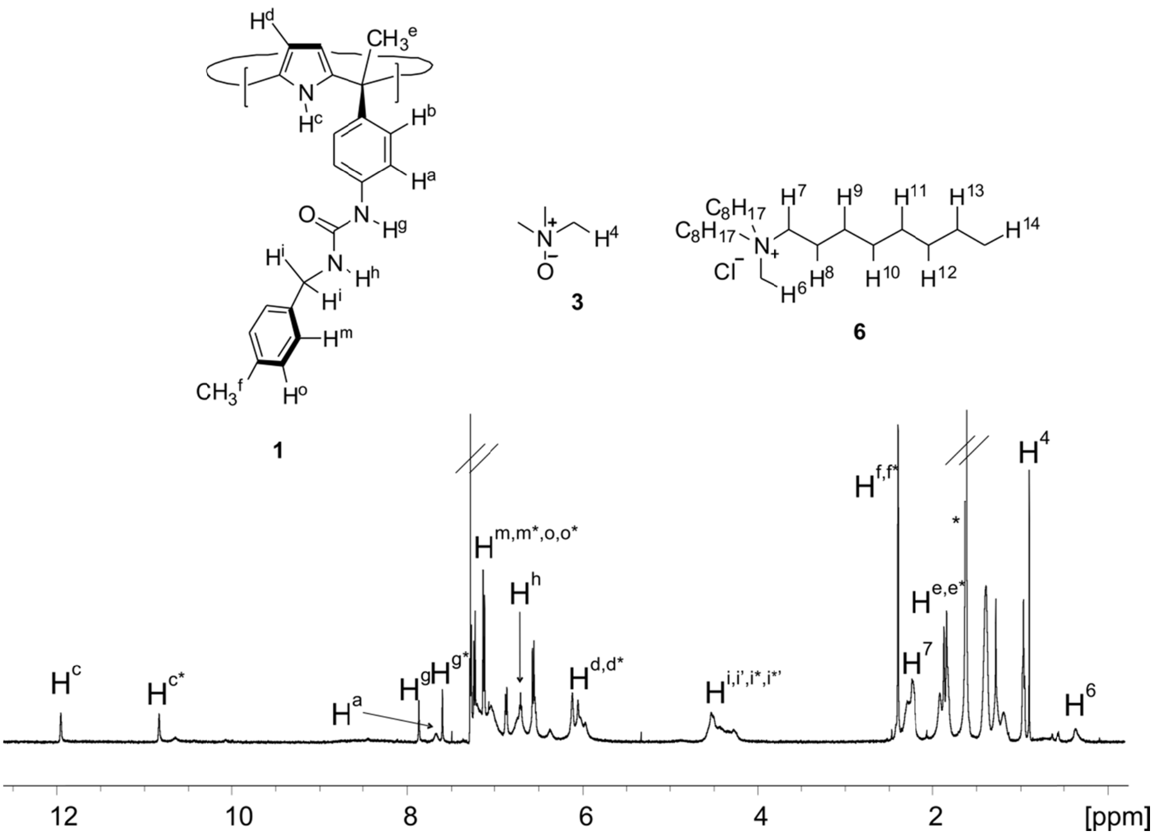

Figure S25. ^{1}H NMR spectrum of a d-chloroform solution of a mixture of tetraurea **1**, trimethylamine *N*-oxide and MTOACl in a 2:1:1 molar ratio. Primed letters indicate non equivalent protons. Letters marked with asterisk indicated protons from different hemispheres. *Residual water.

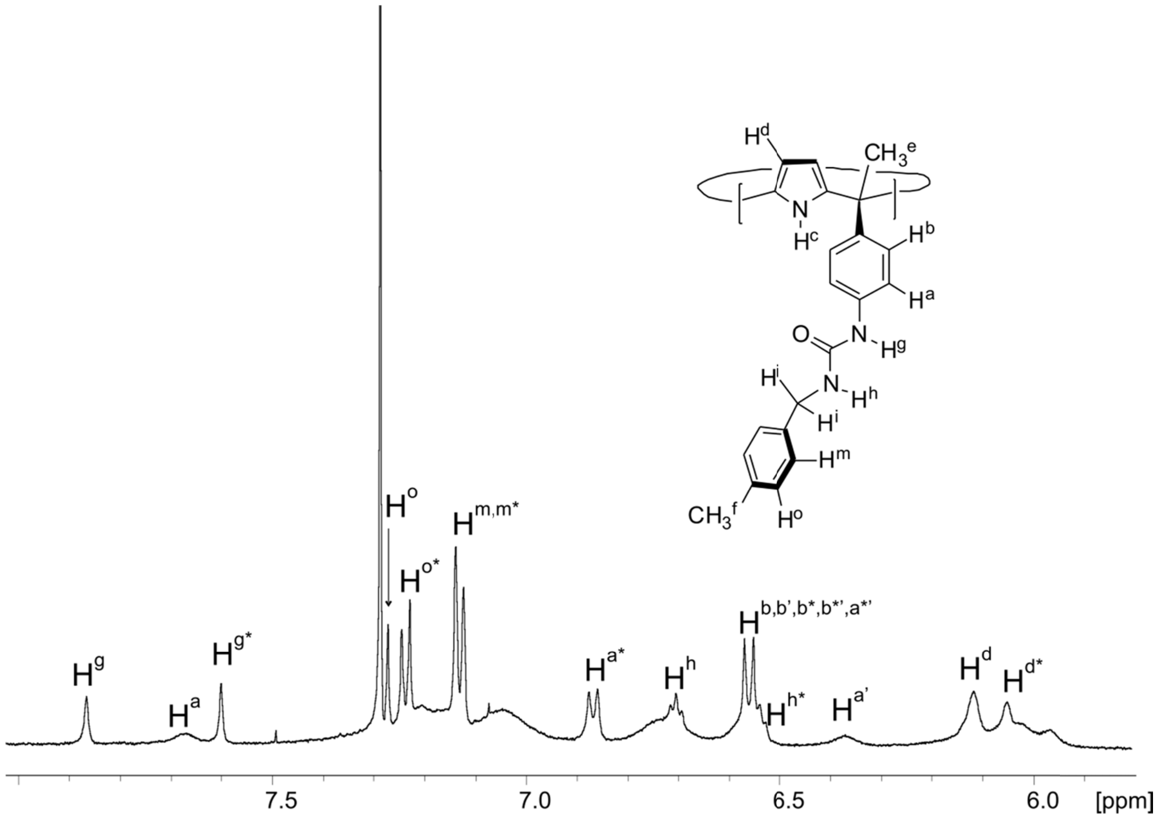

Figure S26. Selected region of ^{1}H NMR spectrum of a d-chloroform solution of a mixture of tetraurea **1**, trimethylamine *N*-oxide and MTOACl in a 2:1:1 molar ratio.

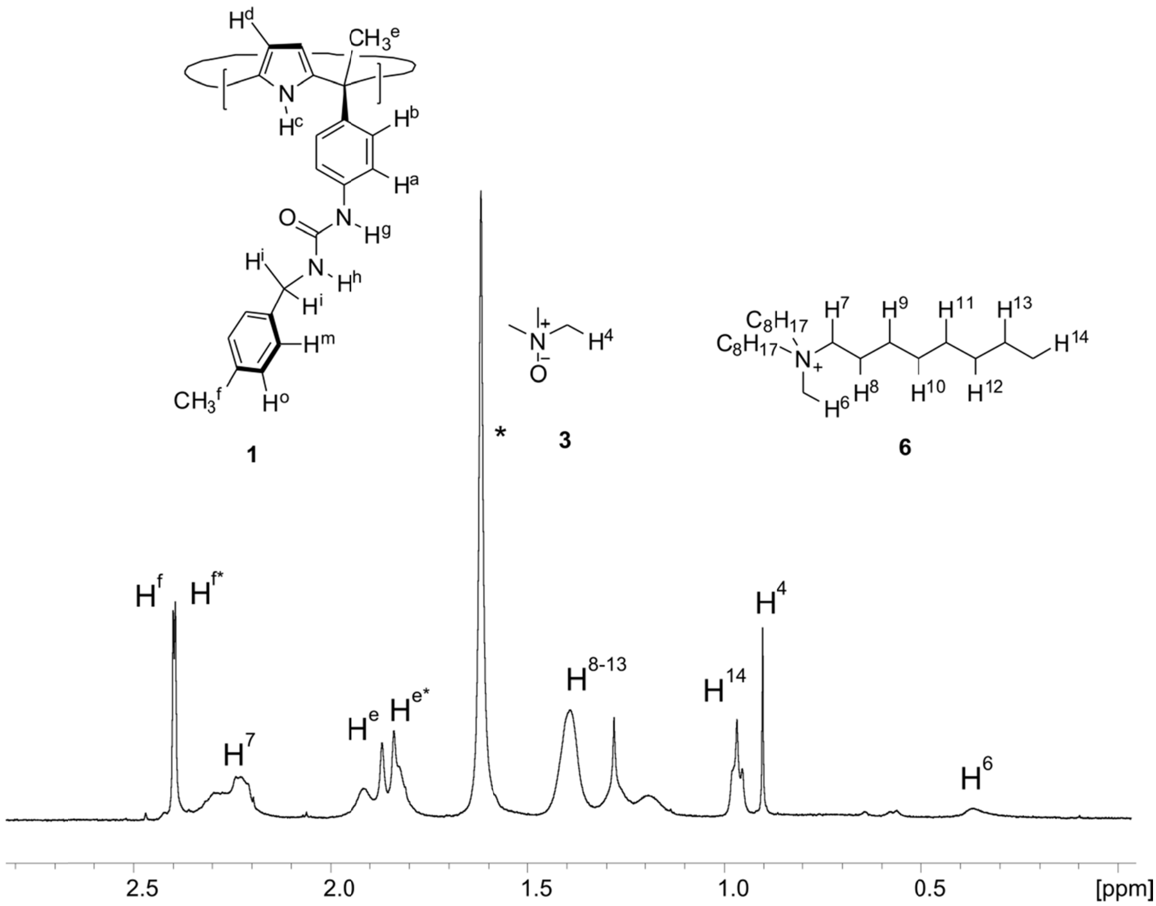

Figure S27. Selected region of ^{1}H NMR spectrum of a d-chloroform solution of a mixture of tetraurea **1**, trimethylamine *N*-oxide and MTOACl in a 2:1:1 molar ratio. *Residual water.

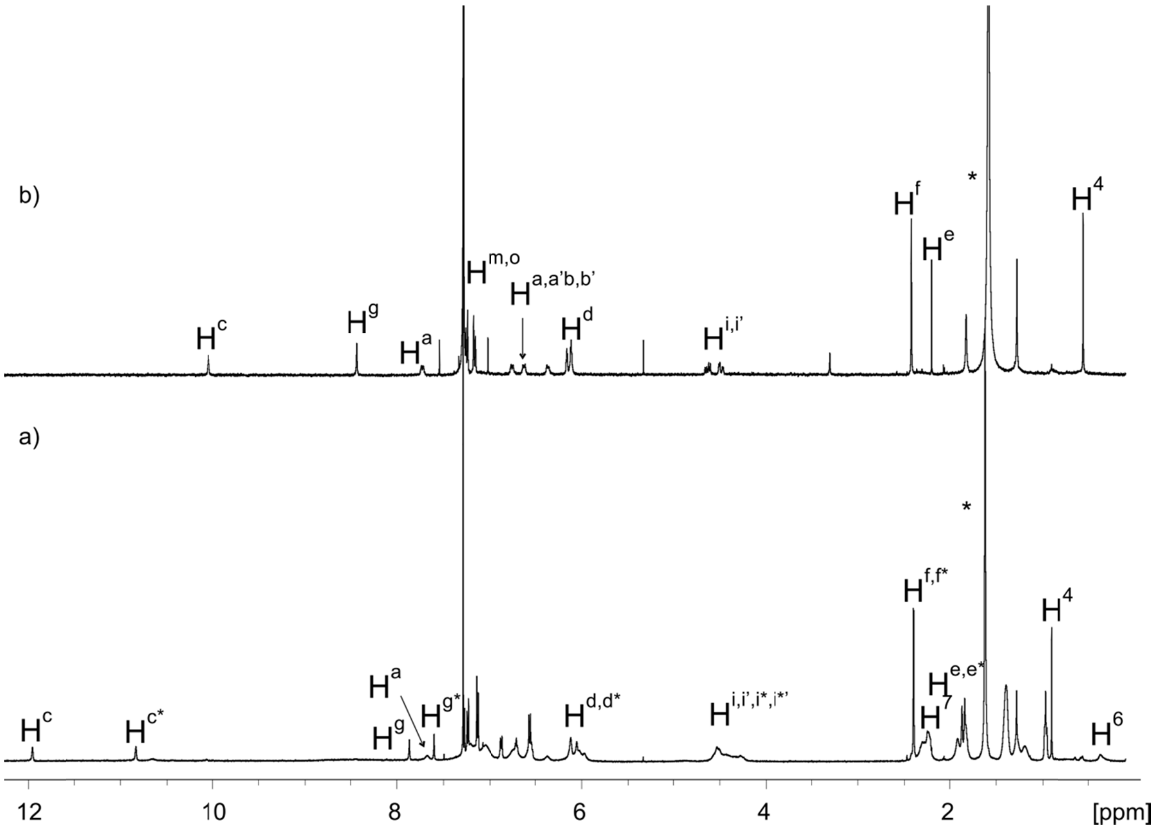

Figure S28. ^{1}H NMR spectra of a d-chloroform solution of tetraurea **1** and a) trimethylamine *N*-oxide and MTOACl in a 2:1:1 molar ratio and b) trimethylamine *N*-oxide in an equimolar ratio. See Figure S25 for proton assignment.

An equimolar amount of trimethylamine *N*-oxide templates the formation of a dimeric capsular assembly with the tetraurea calixpyrrole, $\mathbf{3_2{\subset}1_2}$ (Figure S28b). Equimolar additions of trimethylamine *N*-oxide and MTOACl to a tetraurea solution produces the hetero-pair capsular assembly (Figure S28a). Most notably, when two different guests are co-encapsulated, both hemispheres of the capsule are dissymmetric. The protons for the encapsulated trimethylamine *N*-oxide resonate at 0.54 ppm in the $\mathbf{3_2{\subset}1_2}$ capsule and at 0.90 when co-encapsulated with a chloride anion evidencing the different environment within the dimeric capsule.

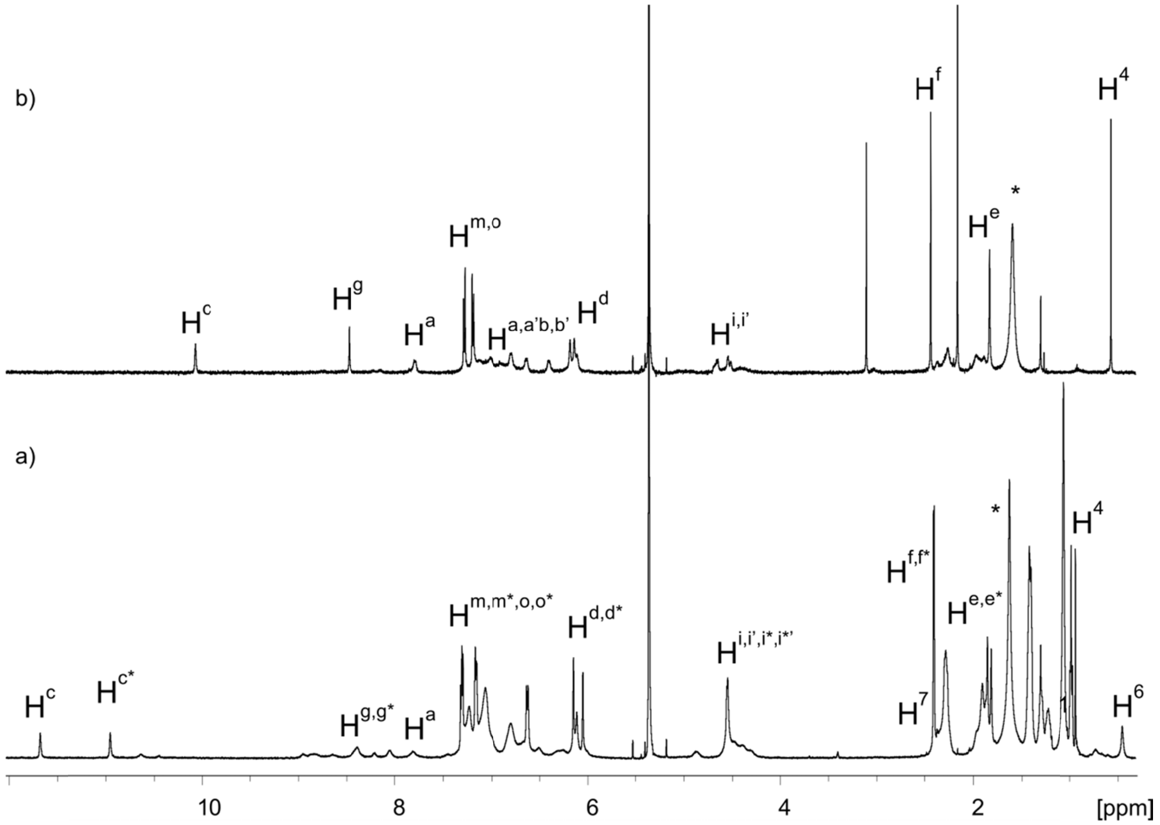

Figure S29. ^{1}H NMR spectra of a d$_2$-dichloromethane solution of tetraurea **1** and a) trimethylamine *N*-oxide and MTOACl in a 2:1:1 molar ratio and b) trimethylamine *N*-oxide in an equimolar ratio. See Figure S25 for proton assignment.

The $\mathbf{3_2 \subset 1_2}$ assembly was formed both in chloroform (Figure S28b) and dichloromethane (Figure S29b). Identically, the hetero-encapsulation complex capsular assembly was formed in both solvents (Figure S28a and Figure S29a) but the ureas NHs protons are better defined in d-chloroform solution. This is due to the co-encapsulation within the capsule of a molecule of solvent. Chloroform provides a tighter packing within the capsule so the urea belt is more stable than in the case of dichloromethane. Dichloromethane is a smaller molecule and the packing is not so tight so the urea belt has to distort a little bit to accommodate the solvent.

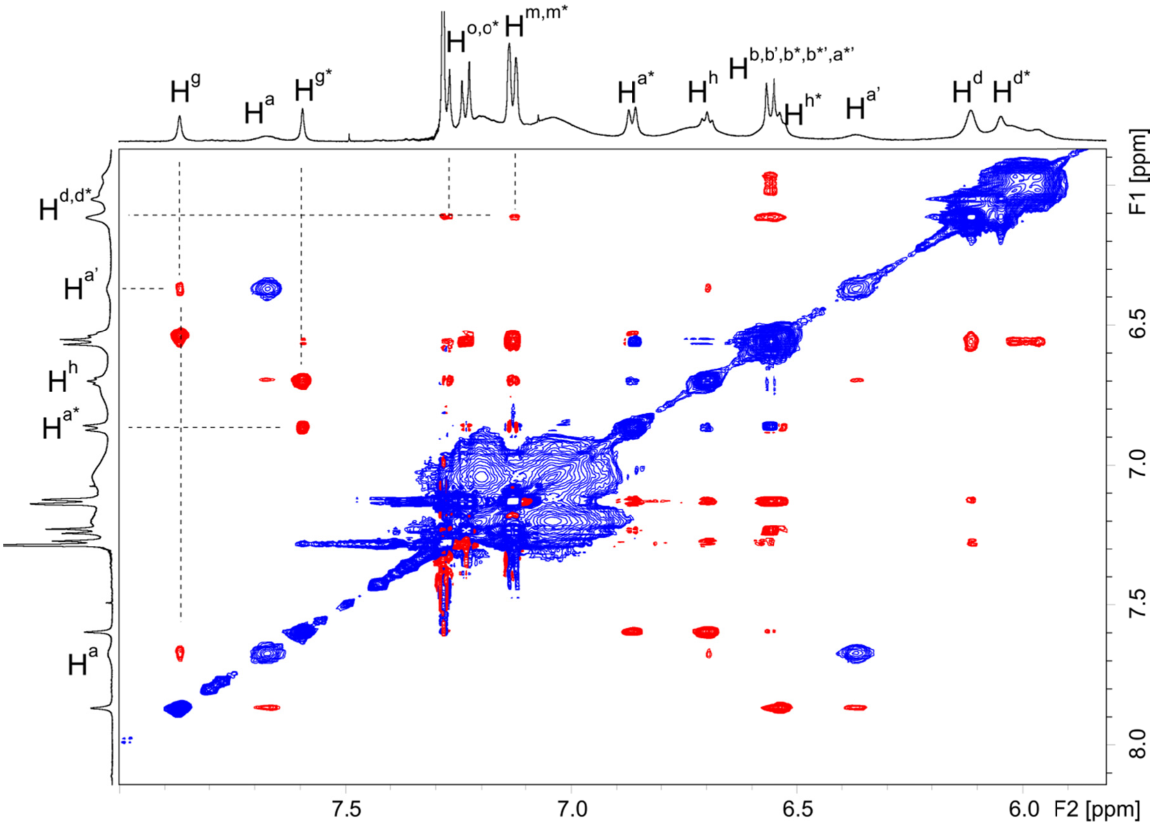

Figure S30. Selected region of a ROESY experiment of a d-chloroform solution of tetraurea **1**, trimethylamine *N*-oxide and MTOACl in 2:1:1 molar ratio. See Figure S25 for proton assignment. Mixing time (d8 = 300 ms).

Pyrrolic protons H^d show close-contact cross-peaks with the aromatic protons H^o and H^m hinting to the formation of a capsular assembly. Urea protons H^g show close-contact cross-peaks with aromatic protons H^a and urea protons H^h, helping in the proton assignment. Aromatic protons H^a appear as non equivalent due to the unidirectional sense of rotation of the urea belt in a capsular assembly. Chemical exchange between Ha protons allows the assignment of the non equivalent H^a protons.

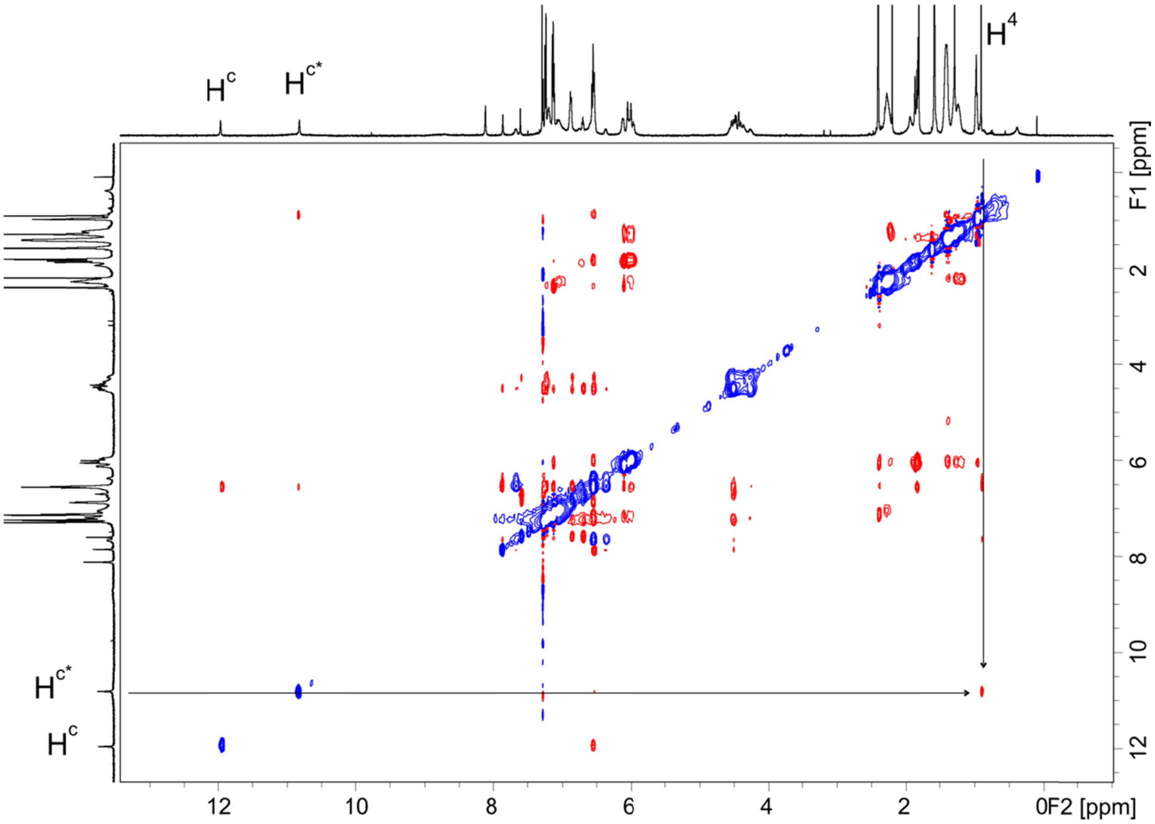

Figure S31. ROESY experiment of a d-chloroform solution of tetraurea **1**, trimethylamine *N*-oxide and MTOACl in 2:1:1 molar ratio. See Figure S25 for proton assignment. Mixing time (d8 = 300 ms).

The close-contact cross-peak between included protons of trimethylamine *N*-oxide and the pyrrolic NHs resonating at δ = 10.85 ppm allowed the assignment of the pyrrole NH protons appearing at δ = 11.96 ppm to the ones forming hydrogen bonds with the encapsulated chloride.

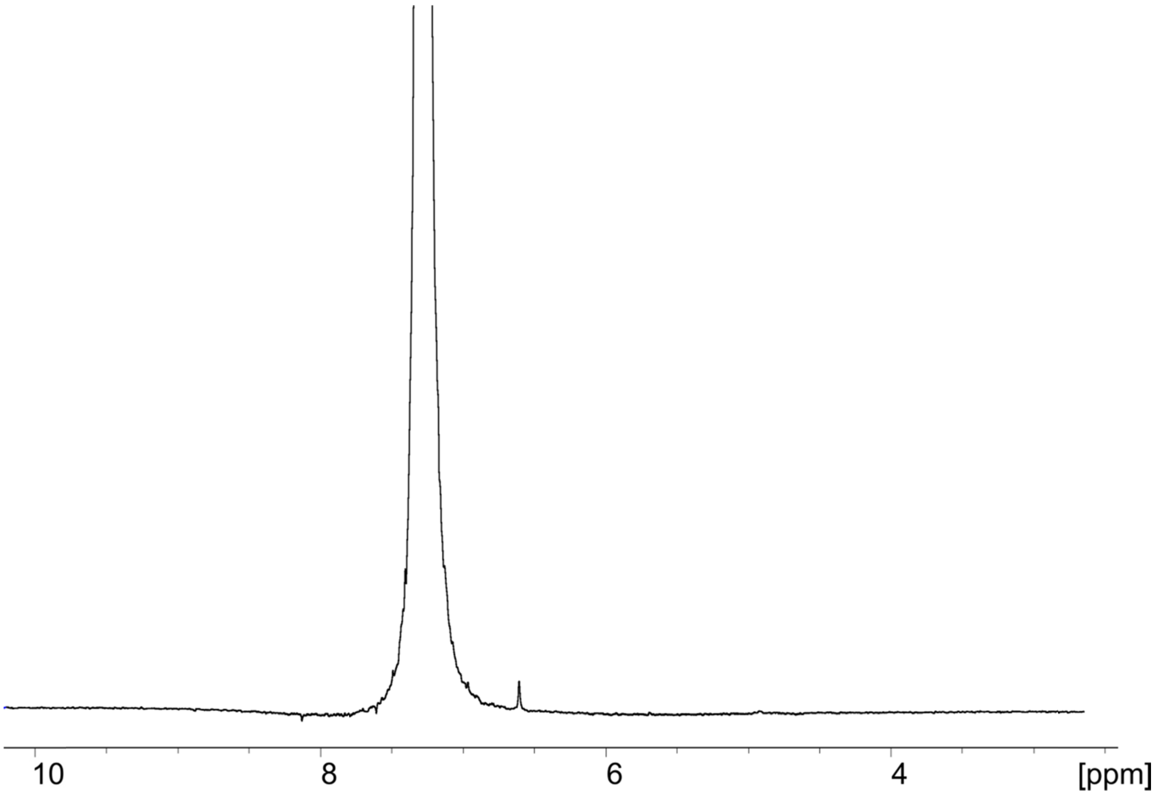

Figure S32. GOESY ^1H NMR spectrum performed at 253 K using a $CHCl_3$ solution of tetraurea **1**, trimethylamine *N*-oxide and MTOACl in 2:1:1 molar ratio: The irradiated signals was that of the bulk solvent (δ = 7.27 ppm). The co-included chloroform in the capsular assembly (**3**•$CHCl_3$•Cl^-)⊂$\mathbf{1}_2$•$MTOA^+$ is experiencing a slow chemical exchange process on the NMR timescale with the bulk solvent and it's observed as a separated singlet resonating at δ = 6.60 ppm.

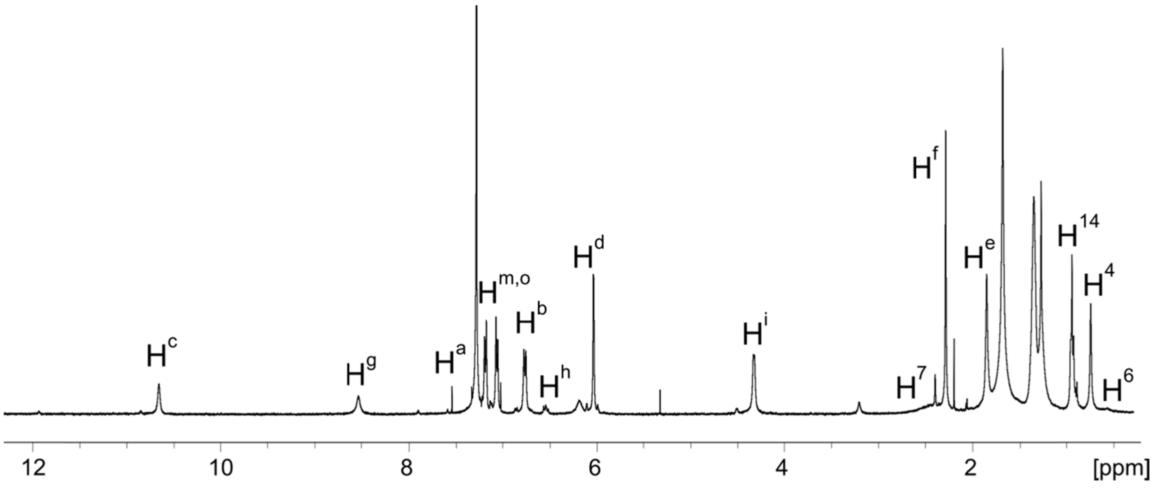

Figure S33. ^{1}H NMR spectrum of a d-chloroform solution of a mixture of tetraurea **1**, trimethylamine *N*-oxide and MTOACl in a 1:1:1 molar ratio. See Figure S25 for proton assignment.

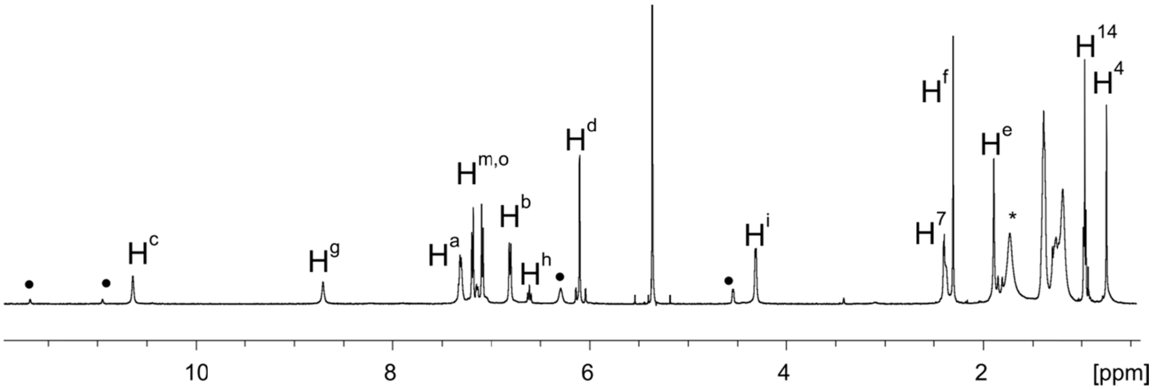

Figure S34. ^{1}H NMR spectrum of a d_2-dichloromethane solution of a mixture of tetraurea **1**, trimethylamine *N*-oxide and MTOACl in a 1:1:1 molar ratio. *Residual water. Protons from the hetero encapsulation complex dimeric capsule ($\mathbf{3}\cdot Cl^-$)$\subset\mathbf{1}_2\cdot$MTOA marked with a black circle. See Figure S25 for proton assignment.

In an equimolar ratio, the mixture of tetraurea, trimethylamine *N*-oxide and MTOACl produces the formation of a four particle complex $\mathbf{3}\subset\mathbf{1}\cdot$MTOA$\cdot Cl^-$ (1:1:1 complex) with C_{4v} symmetry both in dichloromethane (Figure S34) and chloroform (Figure S33). The protons for the included *N*-oxide resonate at 0.75 ppm. As a minor species, there is the dimeric capsule co-encapsulating a molecule of *N*-oxide and a chloride anion (Figure S29a).

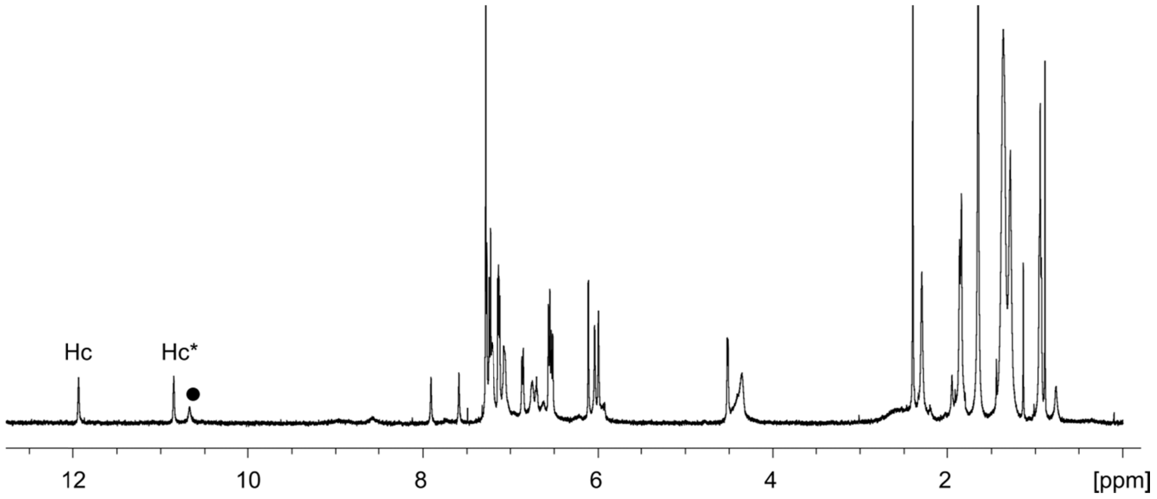

Figure S35. ^{1}H NMR spectrum of a d-chloroform solution of a mixture of tetraurea **1**, trimethylamine *N*-oxide and MTOACl in a 2:1:2 molar ratio. See Figure S25 for proton assignment.

The major species is the hetero-encapsulation complex dimeric capsule (**3** $\cdot$Cl^{-})$\subset$**1**$_2$$\cdot$MTOA (Figure S25) and the 1:1:1 complex **3**$\subset$**1**$\cdot$MTOA$\cdot$Cl^{-} (marked with a black spot, Figure S33).

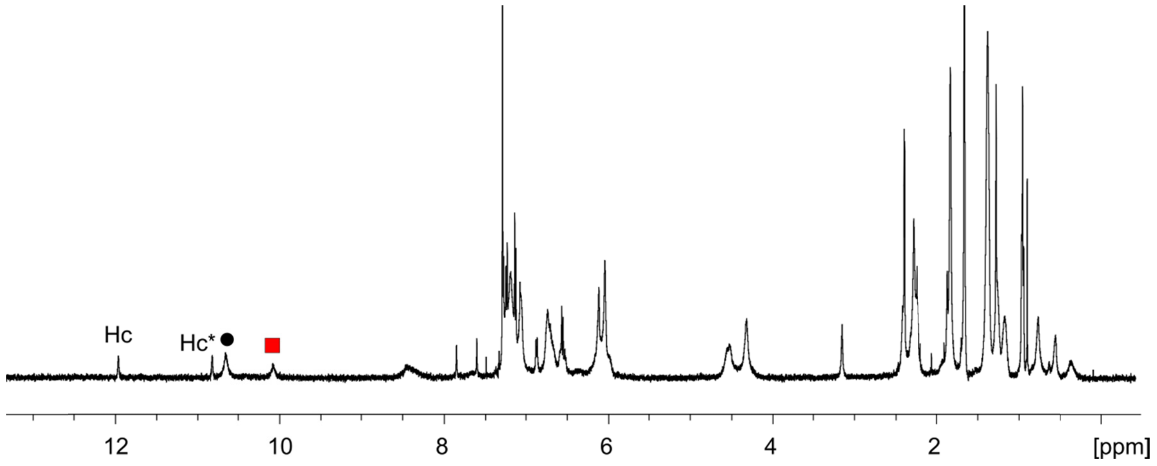

Figure S36. ^{1}H NMR spectrum of a d-chloroform solution of a mixture of tetraurea **1**, trimethylamine *N*-oxide and MTOACl in a 2:2:1 molar ratio. See Figure S25 for proton assignment.

The observed species are the hetero-encapsulation complex in the dimeric assembly (Figure S25), the 1:1:1 complex (marked with a black circle, Figure S33) and the **3**$_2$$\subset$**1**$_2$ capsule (marked with a red square, Figure S28b).

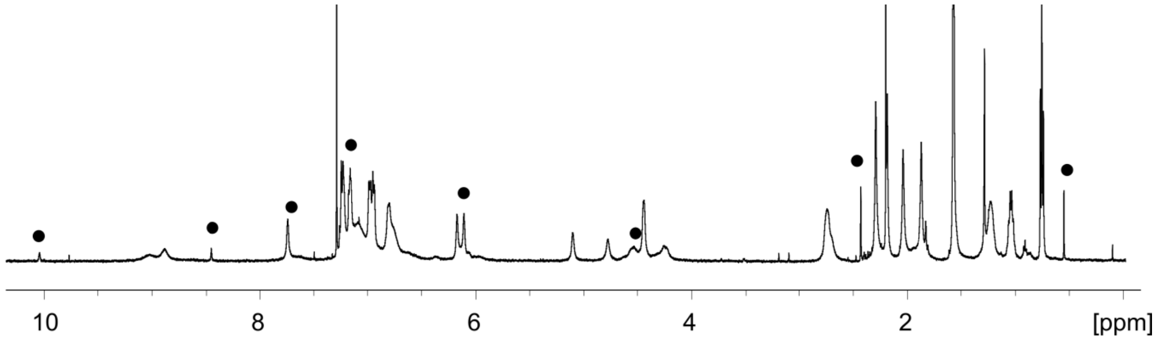

Figure S37. ^{1}H NMR spectrum of a d-chloroform solution of a mixture of tetraurea **1**, trimethylamine *N*-oxide and TBACl in a 2:1:1 molar ratio. Protons from the $\mathbf{3}_2\subset\mathbf{1}_2$ capsule marked with a black spot.

TBACl does not produce the hetero-encapsulation assembly but the pairwise capsular assembly $\mathbf{3}_2\subset\mathbf{1}_2$ (Figure S28b) and ill-defined aggregates.

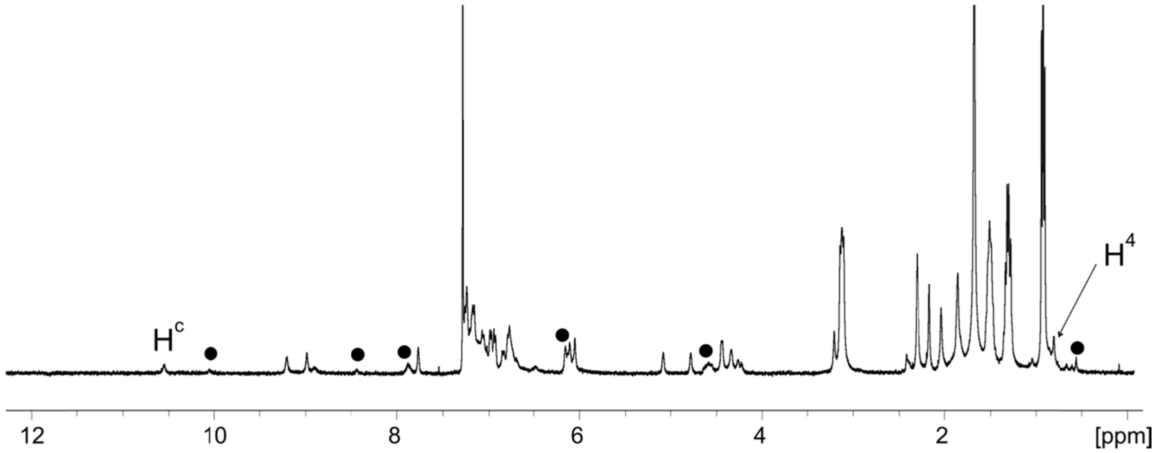

Figure S38. ^{1}H NMR spectrum of a d-chloroform solution of a mixture of tetraurea **1**, trimethylamine *N*-oxide and TBACl in an equimolar ratio. Protons from the $\mathbf{3}_2\subset\mathbf{1}_2$ capsule marked with a black spot. See Figure S25 for proton assignment.

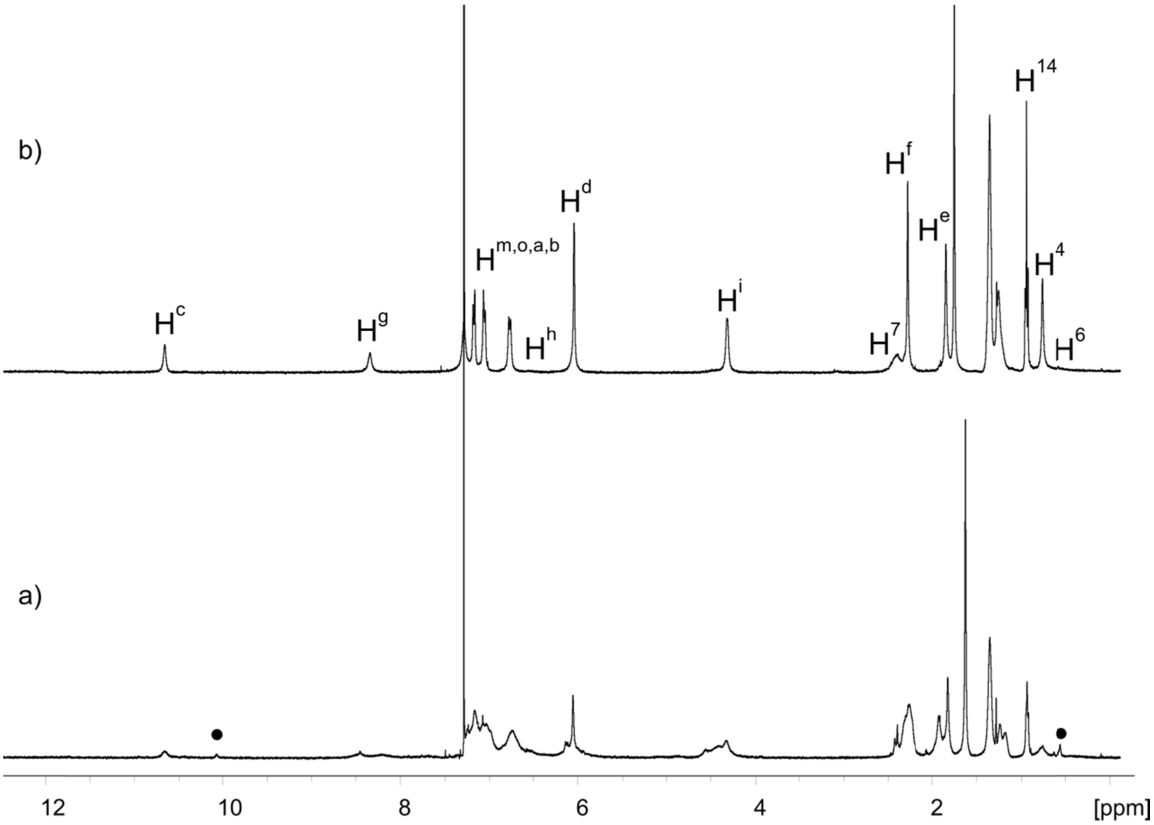

Figure S39. ^{1}H NMR spectrum of a d-chloroform solution of a mixture of tetraurea **1**, trimethylamine *N*-oxide and MTOABr in a a) 2:1:1 molar ratio b)1:1:1 molar ratio. Protons from the $\mathbf{3}_2\subset\mathbf{1}_2$ capsule marked with a black spot.

In an equimolar mixture of tetraurea, trimethylamine *N*-oxide and MTOABr, the only species in solution is the 1:1:1 complex, with the *N*-oxide included in the cavity of the calixpyrrole, the cation at the shallow cup of the calixpyrrole and the bromide interacting with the ureas. In a 2:1:1 molar ratio we observe both the 1:1:1 complex, the $\mathbf{3}_2\subset\mathbf{1}_2$ capsule and ill-defined aggregates.

NMR experiments of tetraurea 1, betaines and ion-pairs.

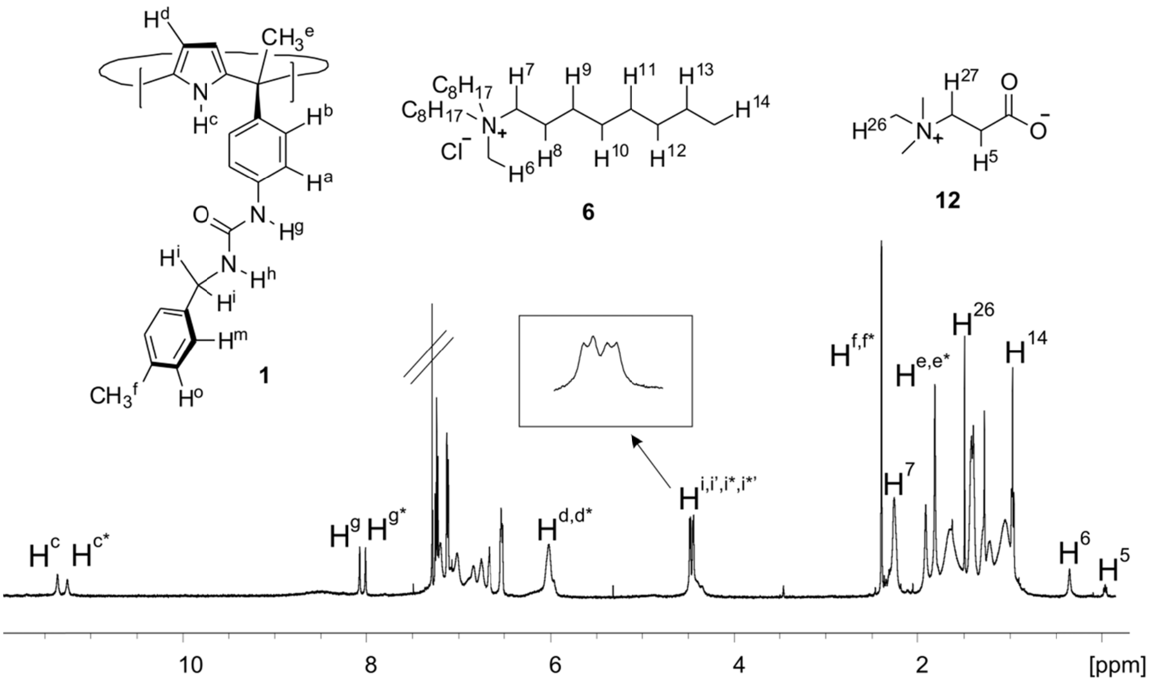

Figure S40. ^1H NMR spectrum of a d-chloroform solution of tetraurea **1**, betaine **12** and MTOACl in a 2:1:1 molar ratio. Letters marked with asterisk indicated protons from different hemispheres.

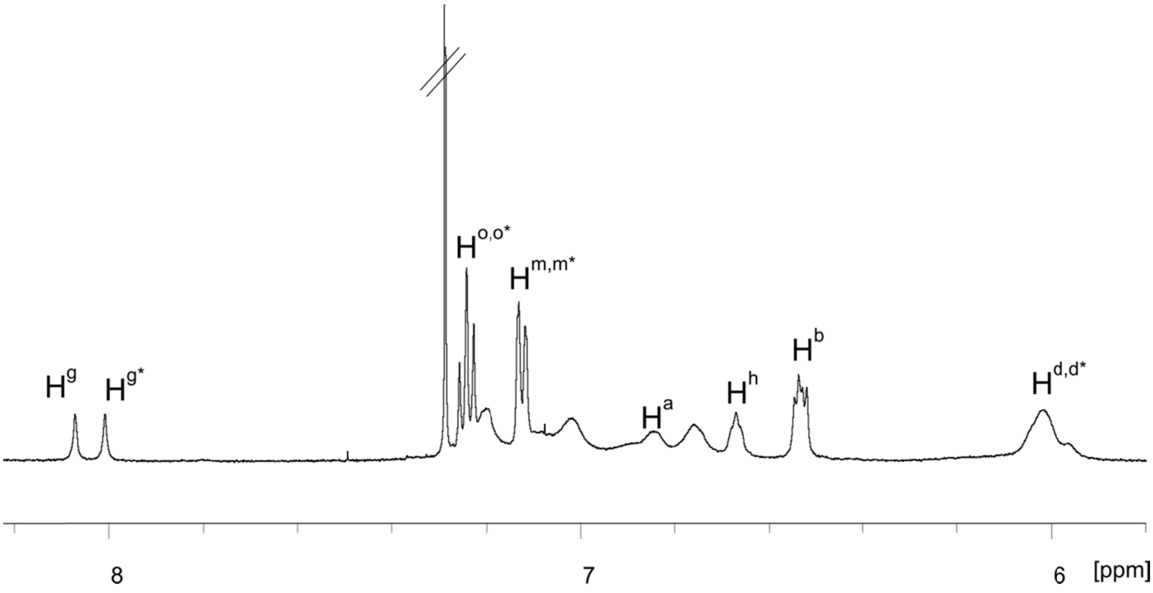

Figure S41. Selected region of ^1H NMR spectrum of a d-chloroform solution of tetraurea **1**, betaine **12** and MTOACl in a 2:1:1 molar ratio. See Figure S40 for proton assignment.

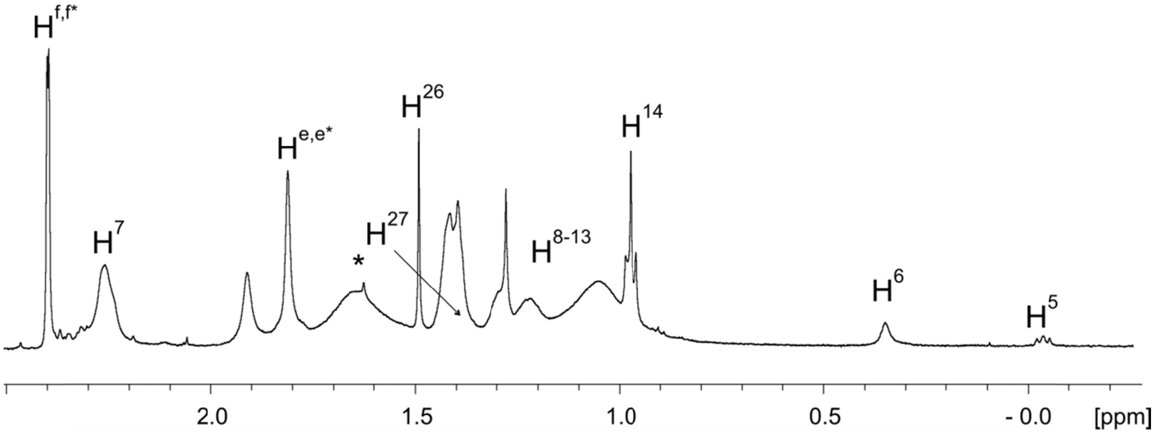

Figure S42. Selected region of ^{1}H NMR spectrum of a d-chloroform solution of tetraurea **1**, betaine **12** and MTOACl in a 2:1:1 molar ratio. See Figure S40 for proton assignment. *Residual water.

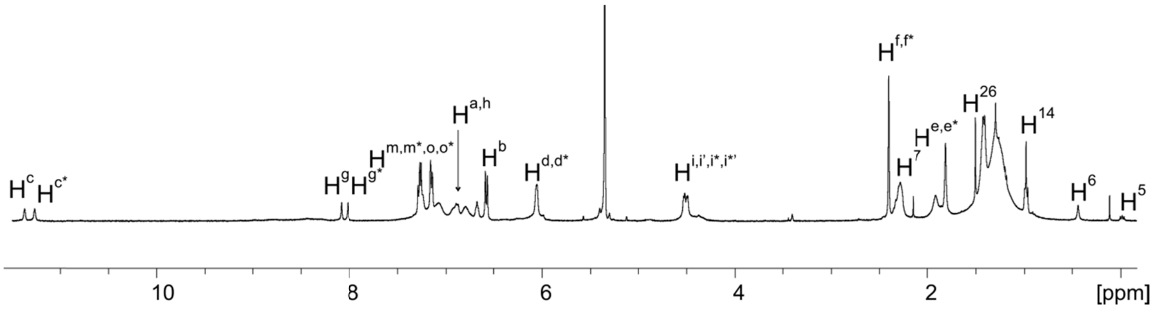

Figure S43. ^{1}H NMR spectrum of a d_2-dichloromethane solution of tetraurea **1**, betaine **12** and MTOACl in a 2:1:1 molar ratio. See Figure S40 for proton assignment.

The formation of the hetero-encapsulation complex (**12**·Cl¯)⊂**1**$_2$·MTOA takes place both in chloroform (Figure S40) and dichloromethane (Figure S43). Both NMR spectra are identical evidencing the no co-encapsulation of a molecule of solvent.

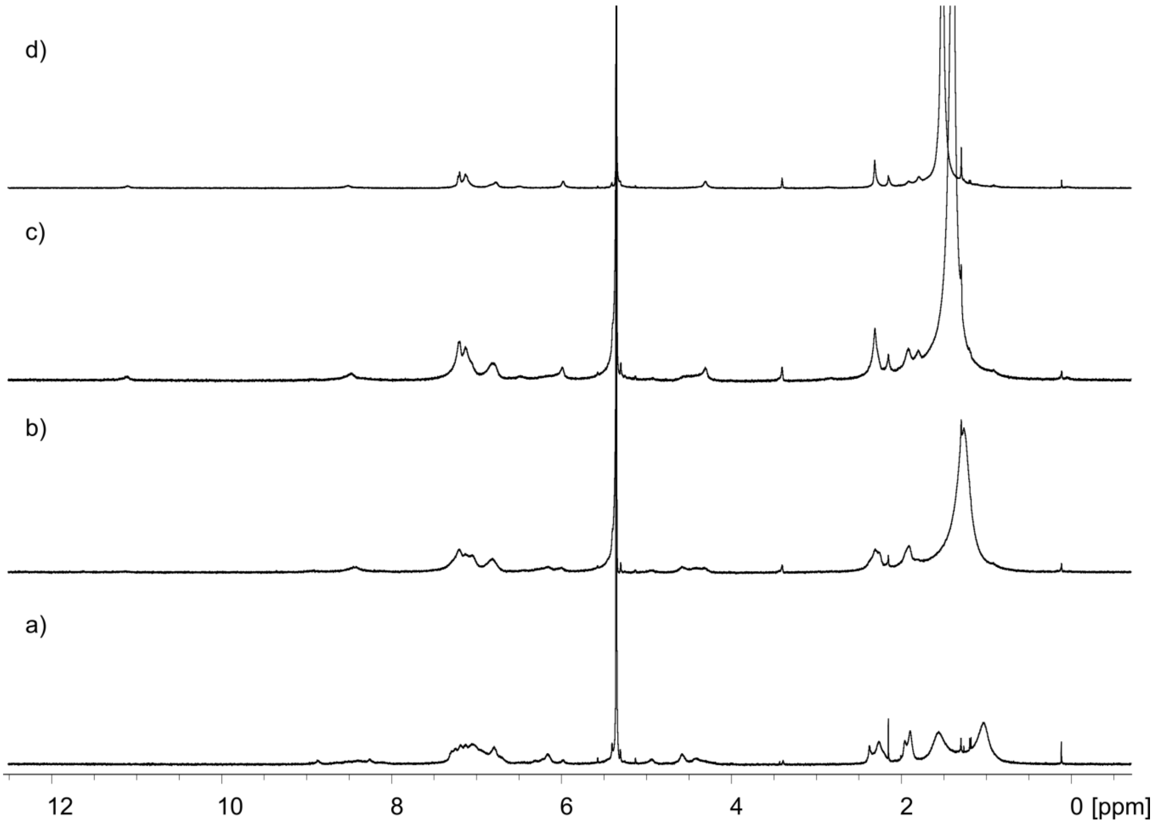

Figure S44. ^{1}H NMR titration of a d_2-dichloromethane suspension of tetraurea **1** with betaine **12** a) 0 eq. b) 0.5 eq. c) 1 eq. d) 1.5 eq.

The addition of betaine **12** to a suspension of tetraurea **1** did not break the aggregation between urea groups to form well-defined species.

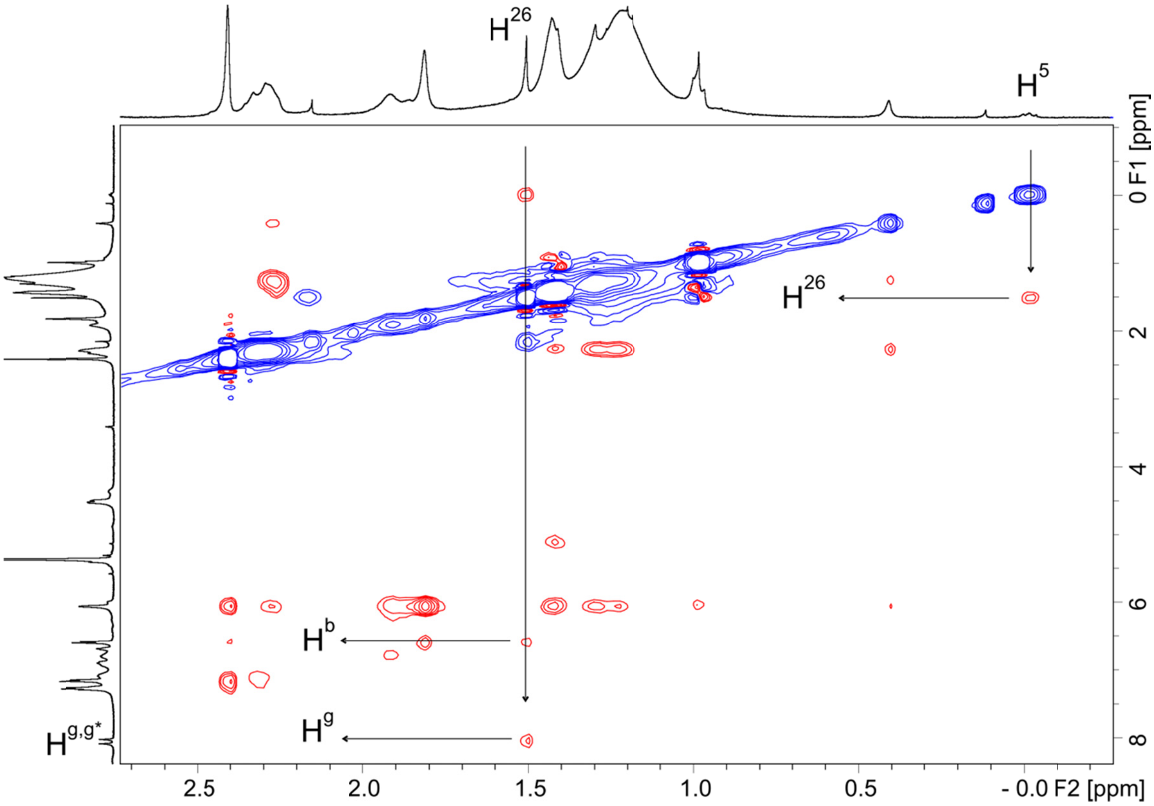

Figure S45. ROESY experiment of a d_2-dichloromethane solution of tetraurea **1**, betaine **12** and MTOACl in 2:1:1 molar ratio showing close-contact between the included betaine and the aromatic protons of the capsule. See Figure S40 for proton assignment. Mixing time (d8 = 300 ms).

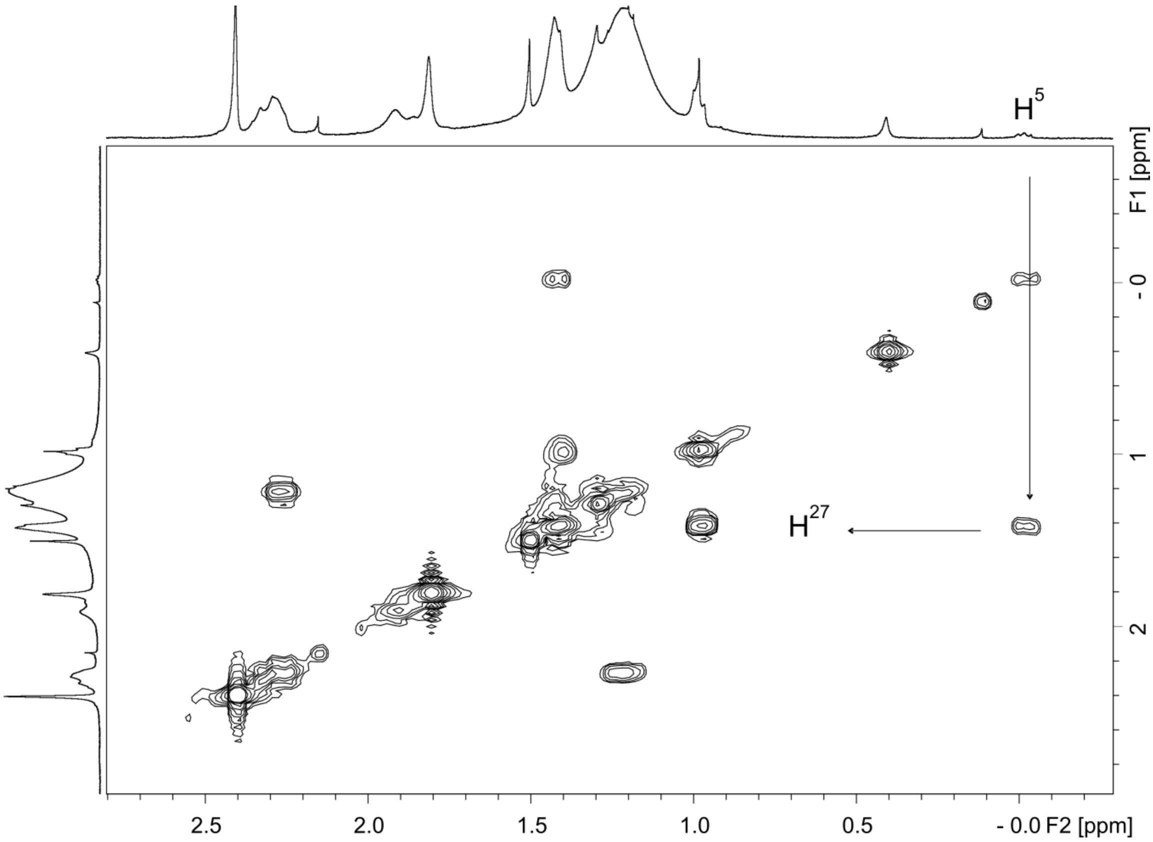

Figure S46. COSY experiment of a d_2-dichloromethane solution of tetraurea **1**, betaine **12** and MTOACl in 2:1:1 molar ratio. See Figure S40 for proton assignment.

COSY NMR reveals the location of the bound methylene protons H^{27} of betaine **12**, hidden under the cation protons.

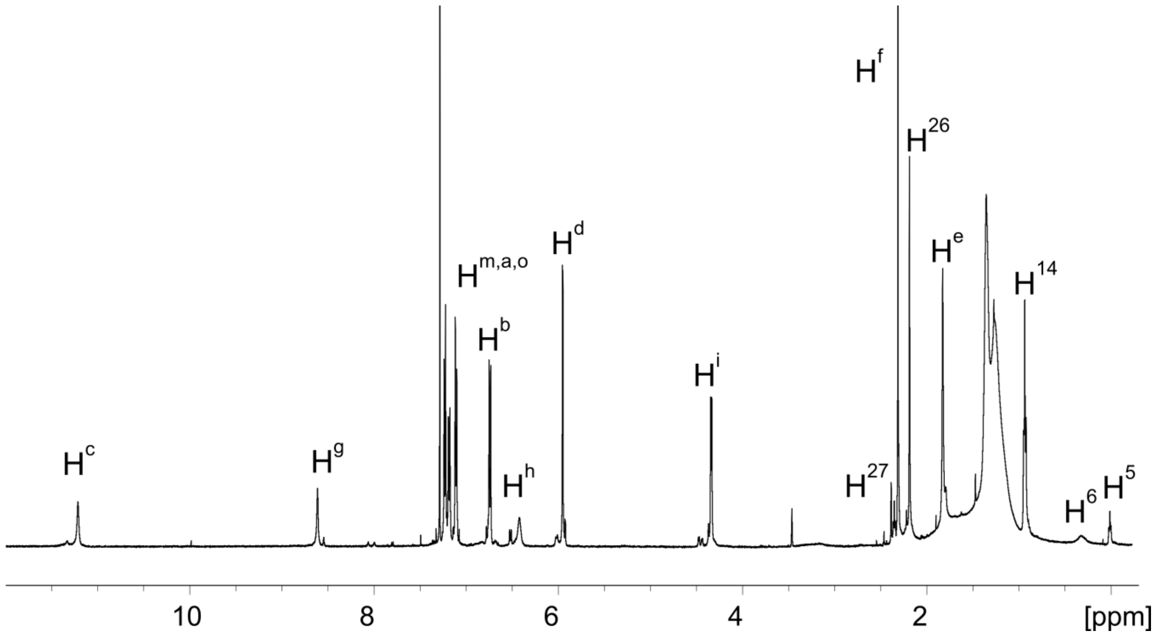

Figure S47. ^{1}H NMR spectrum of a d-chloroform solution of tetraurea **1**, betaine **12** and MTOACl in a 1:1:1 molar ratio. See Figure S40 for proton assignment.

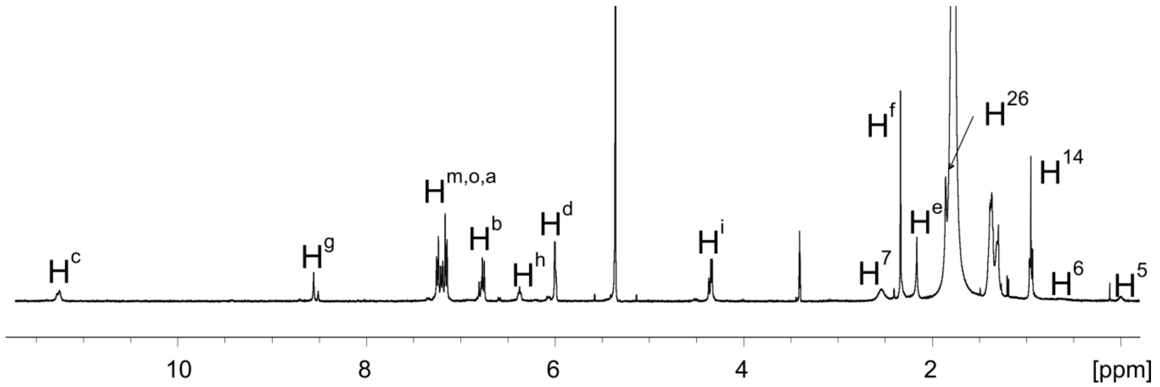

Figure S48. ^{1}H NMR spectrum of a d_2-dichloromethane solution of tetraurea **1**, betaine **12** and MTOACl in a 1:1:1 molar ratio. See Figure S40 for proton assignment.

In an equimolar stoichiometry of urea, betaine and salt, a 1:1:1 complex (four particle assembly, C_{4v} symmetry) is formed both in chloroform (Figure S47) and dichloromethane (Figure S48). The betaine is included in the calixpyrrole cavity, the cation is located at the shallow cup of the calixpyrrole and the chloride interacts with the urea groups at the upper rim.

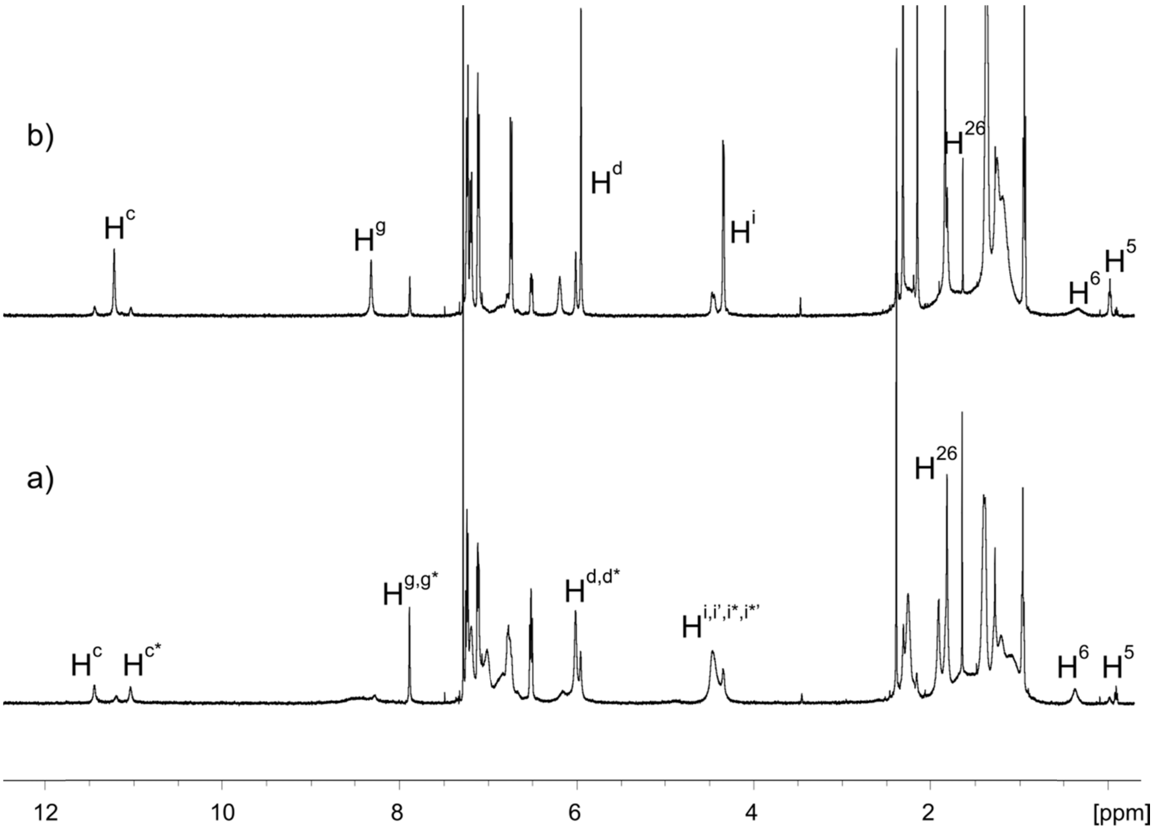

Figure S49. ^{1}H NMR spectra of a d-chloroform solution of tetraurea **1**, betaine **12** and BrMTOA in a a) 2:1:1 molar ratio b) 1:1:1 molar ratio.

In an equimolar mixture of tetraurea, betaine **12** and MTOABr (Figure S49b) the major species is the 1:1:1 complex, where a molecule of betaine is included in the cavity of the calixpyrrole, and the bromide is interacting with the urea groups. The minor species is the hetero-encapsulation complex where a betaine and a bromide anion are co-encapsulated within the dimeric tetraurea capsule. In a 2:1:1 molar ratio (Figure S49a), the opposite situation is observed. The major species is the hetero-encapsulation complex and the minor species the 1:1:1 complex.

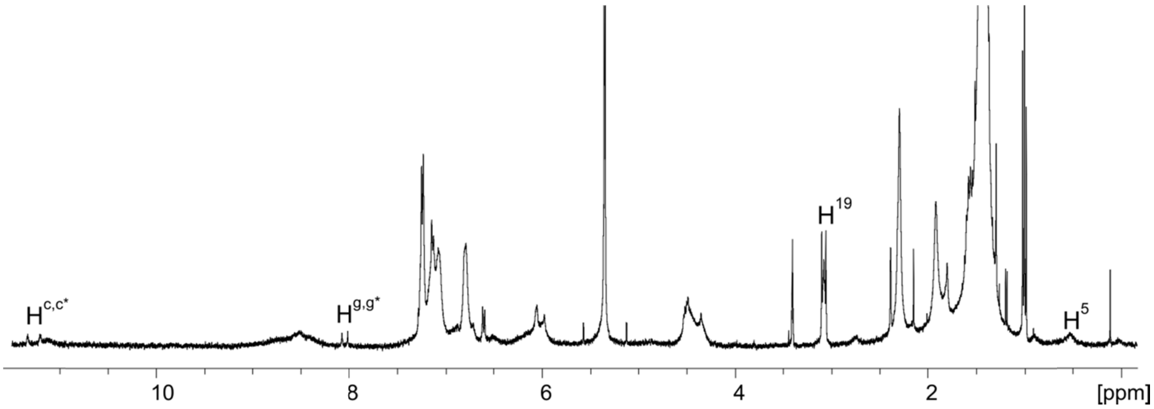

Figure S50. ^{1}H NMR spectrum of a d_2-dichloromethane solution of tetraurea **1**, betaine **12** and TBACl in a 2:1:1 molar ratio. See Figure S40 and Figure S13 for proton assignment.

The addition of betaine **12** and MTOACl to a tetraurea solution in a 1:1:2 molar ratio produces a hetero-encapsulation complex (Figure S43). However, TBA cation is not a well fit for the calixpyrrole shallow cavity and TBACl does not produce the hetero-pair assembly quantitatively (Figure S50).

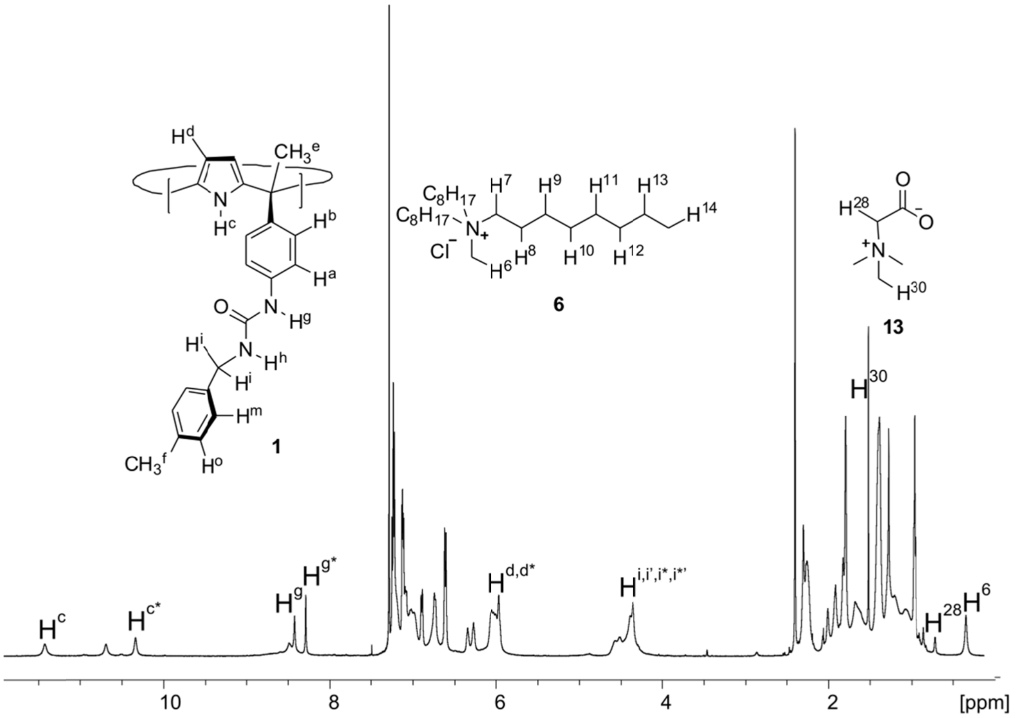

Figure S51. ^{1}H NMR spectra of a d-chloroform solution of a mixture of tetraurea **1**, betaine **13** and MTOACl in a 2:1:1 molar ratio

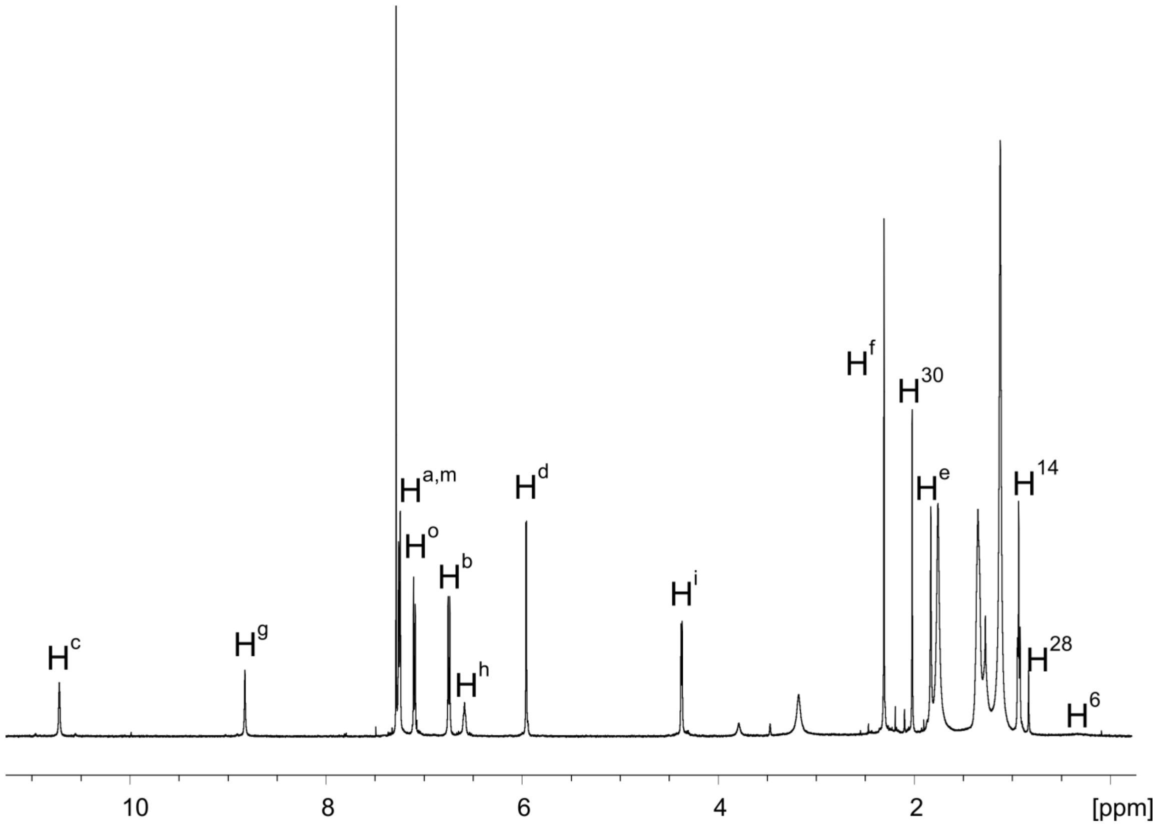

Figure S52. ^{1}H NMR spectra of a d-chloroform solution of a mixture of tetraurea **1**, betaine **13** and MTOACl in a 1:1:1 molar ratio

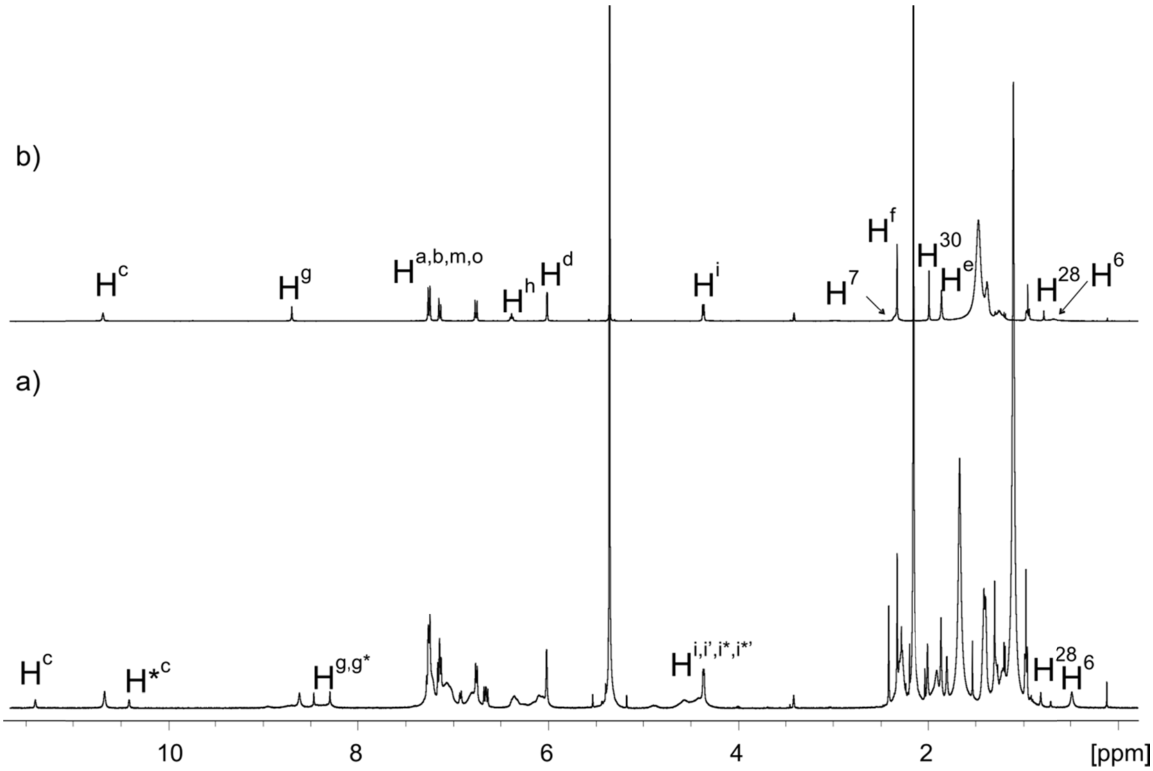

Figure S53. ^{1}H NMR spectra of a d_2-dichloromethane solution of a mixture of tetraurea **1**, betaine **13** and MTOACl in a) 2:1:1 molar ratio b) 1:1:1 molar ratio. See Figure S51 for proton assignment.

The equimolar mixture of tetraurea, betaine **13** and MTOACl produces exclusively the four particle assembly 1:1:1 complex **13**⊂**1**·MTOA·Cl^{-}, where the betaine is included in the calixpyrrole cavity, the cation is at the shallow cavity of the calixpyrrole and the chloride is interacting with the urea groups both in chloroform (Figure S52) and dichloromethane (Figure S53b). When the urea, betaine **13** and MTOACl are a 2:1:1 molar ratio, we observe in solution a mixture of the 1:1:1 complex and the hetero-encapsulation complex both in chloroform (Figure S51) and dichloromethane (Figure S53a) where the betaine and chloride are co-encapsulated within dimeric tetraurea capsule $\mathbf{1_2}$.

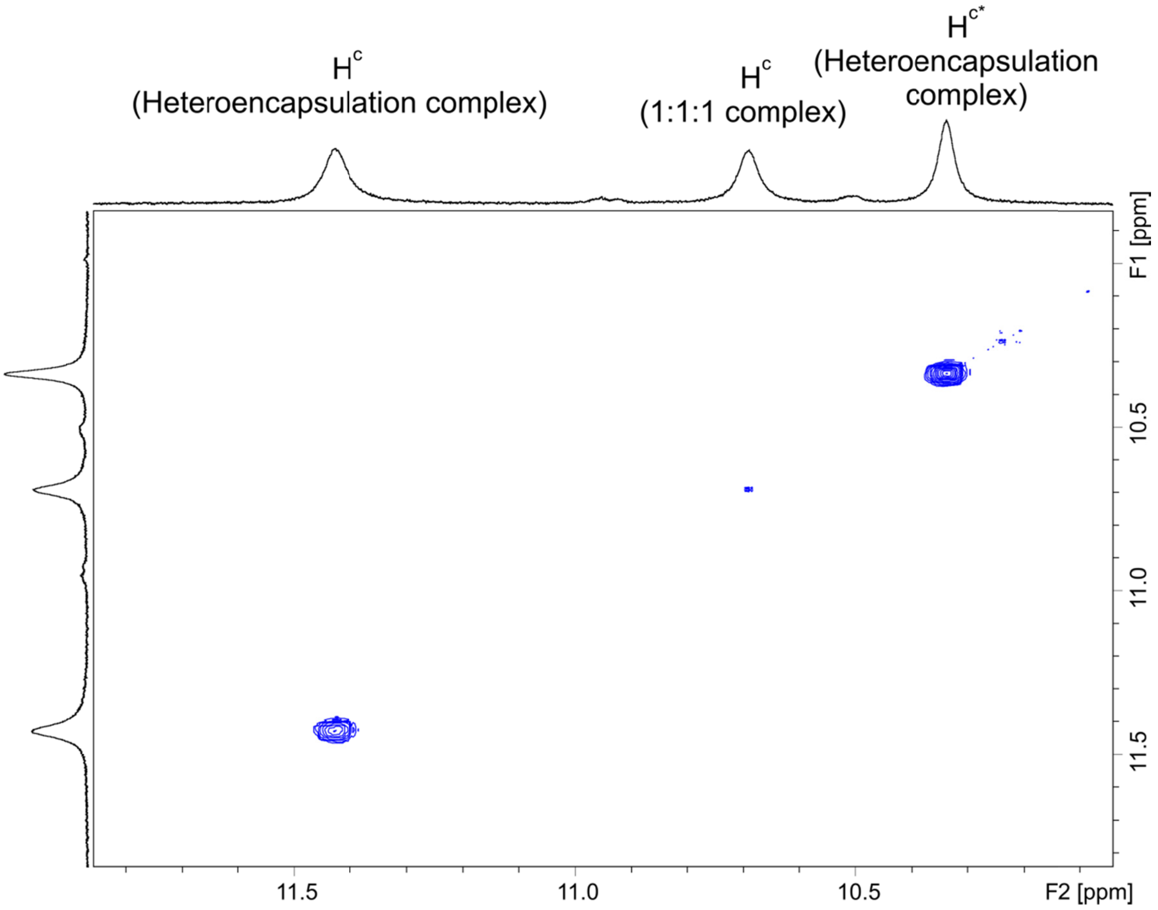

Figure S54. Selected region of a 2D EXSY NMR of a d-chloroform solution of tetraurea, betaine 13 and MTOACl in a 2:1:1 molar ratio showing no chemical exchange between the two species in solution: the four particle assembly (1:1:1 complex) and the dimeric capsule (heteroencapsulation complex) in the EXSY time scale. Mixing time (d8 = 300 ms).

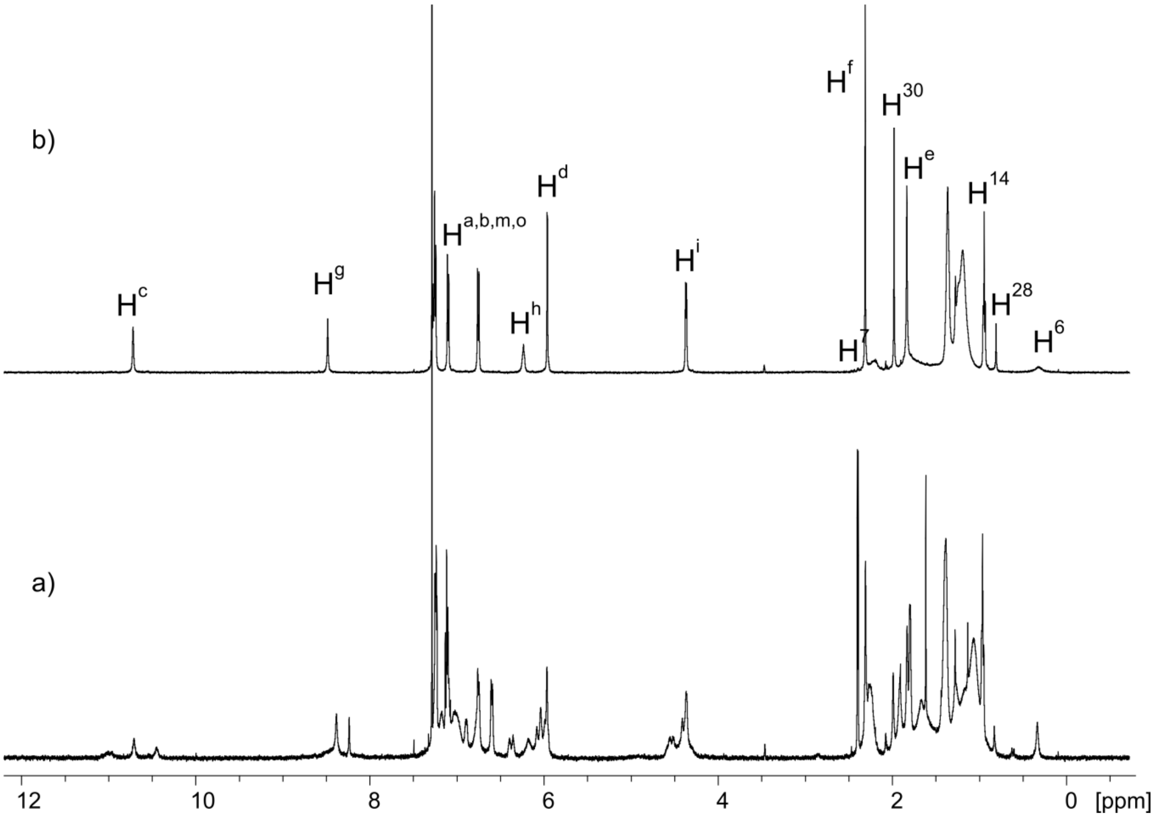

Figure S55. ^{1}H NMR spectra of a d-chloroform solution of tetraurea **1**, betaine **13** and BrMTOA in a a) 2:1:1 molar ratio b) 1:1:1 molar ratio

In an equimolar mixture of tetraurea, betaine **13** and MTOABr (Figure S55b) the observed species is the four particle 1:1:1 complex, where a molecule of betaine is included in the cavity of the calixpyrrole, and the bromide is interacting with the urea groups. In a 2:1:1 molar ratio (Figure S55a), we observe a mixture of hetero-encapsulation complex and 1:1:1 complex.

DOSY NMR experiments

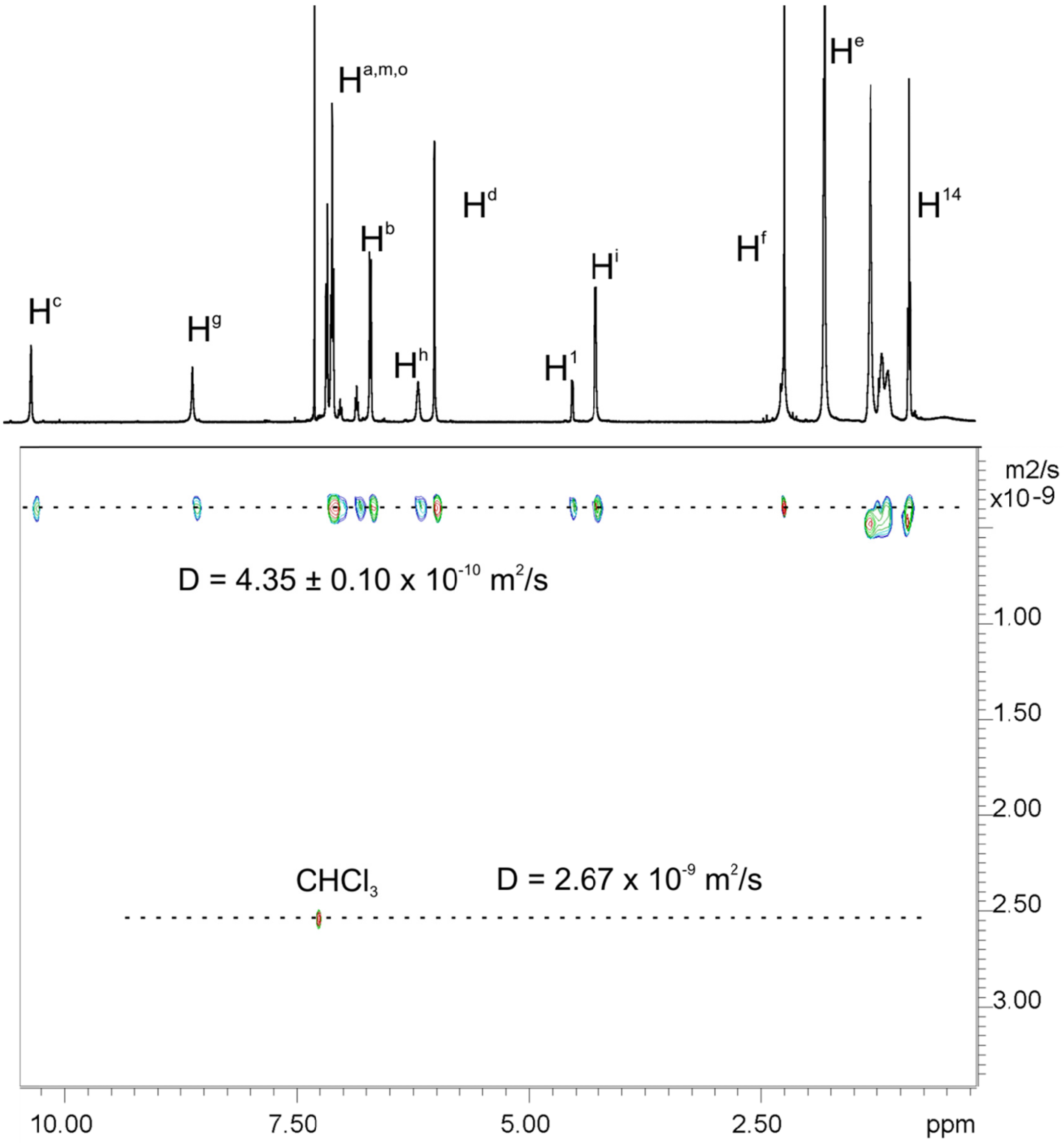

Figure S56. ^{1}H pseudo-2D DOSY plot of **5**⊂**1**·MTOA·Cl^{-}four-particle assembly. Data fitted to a monoexponential function. See Figure S2 for proton assignment.

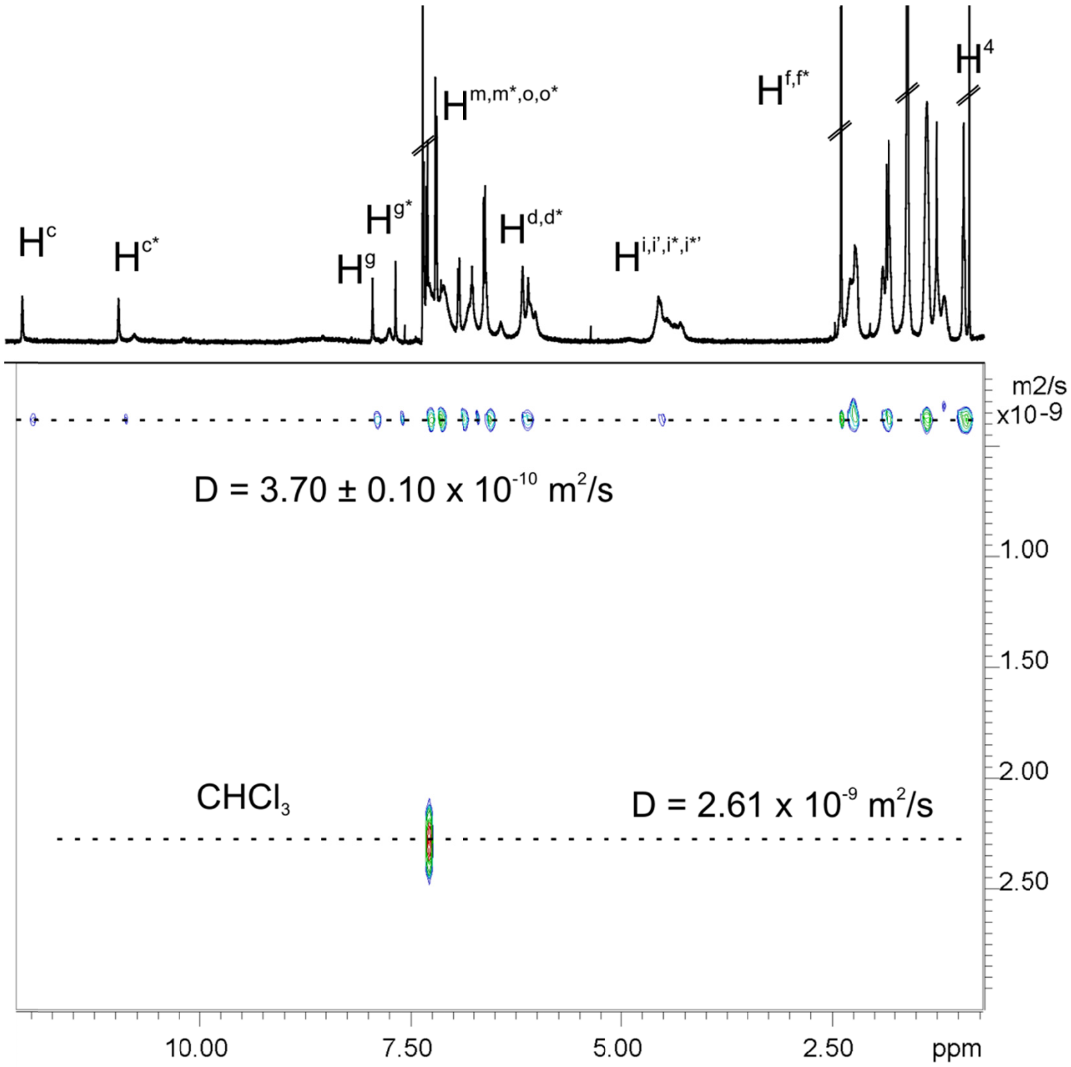

Figure S57. [1]H pseudo-2D DOSY plot of ($\mathbf{3}$·$CHCl_3$·Cl^-)⊂$\mathbf{1}_2$·MTOA capsular assembly. Data fitted to a monoexponential function. See Figure S25 for proton assignment.

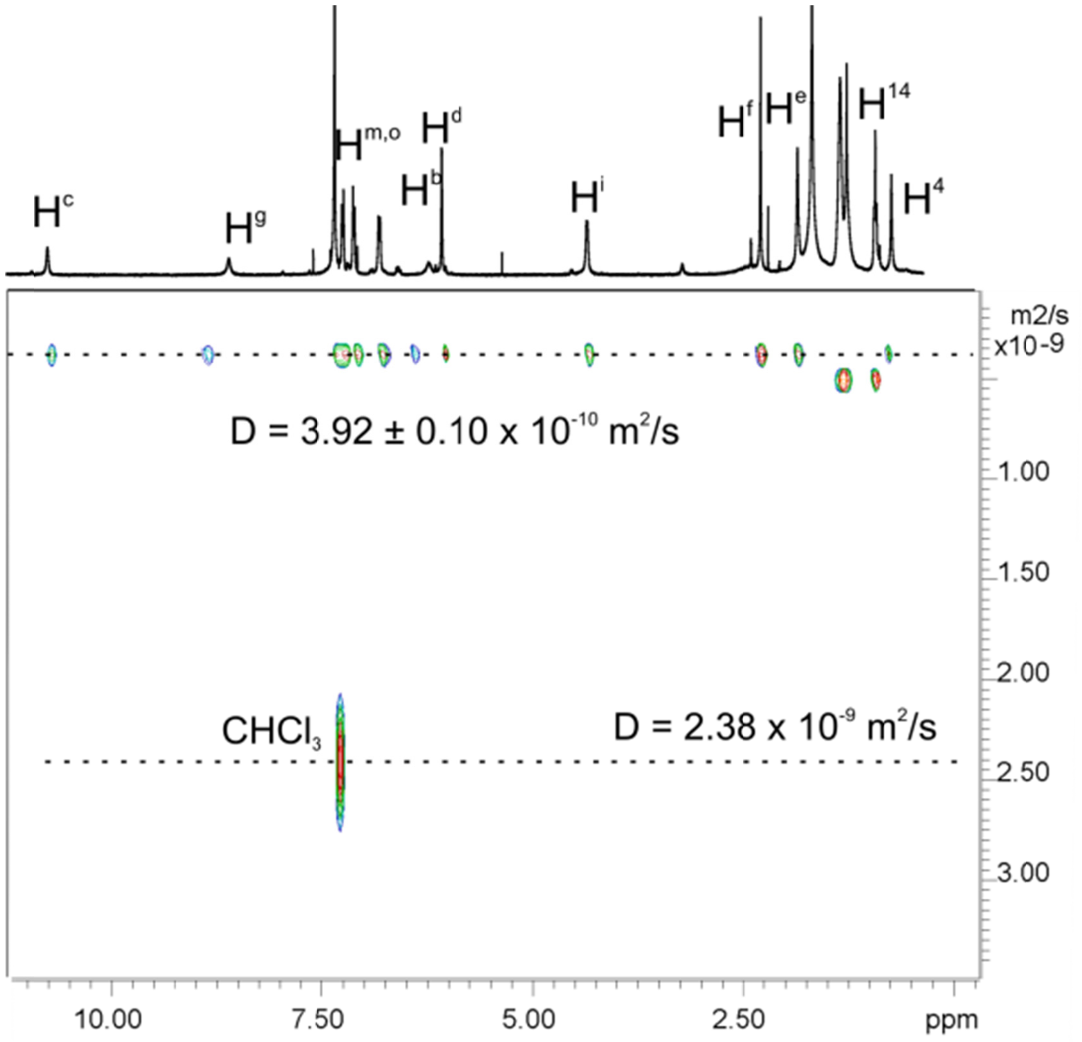

Figure S58. ^{1}H pseudo-2D DOSY plot of **3**⊂**1**·MTOA·Cl$^-$four-particle assembly. Data fitted to a monoexponential function. See Figure S25 for proton assignment.

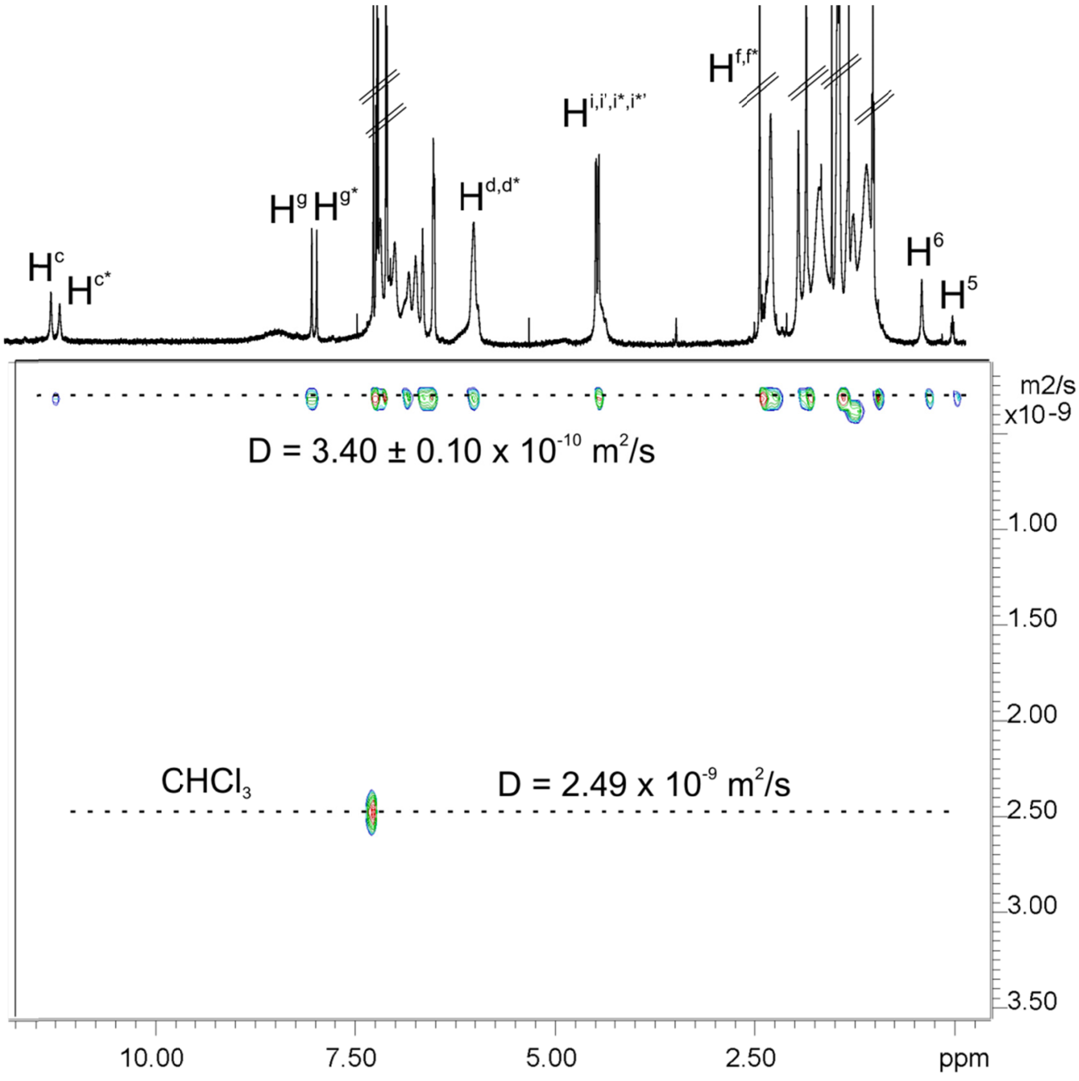

Figure S59. [1]H pseudo-2D DOSY plot of (**12**·Cl$^-$)⊂**1**$_2$·MTOA complex. Data fitted to a monoexponential function. See Figure S40 for proton assignment.

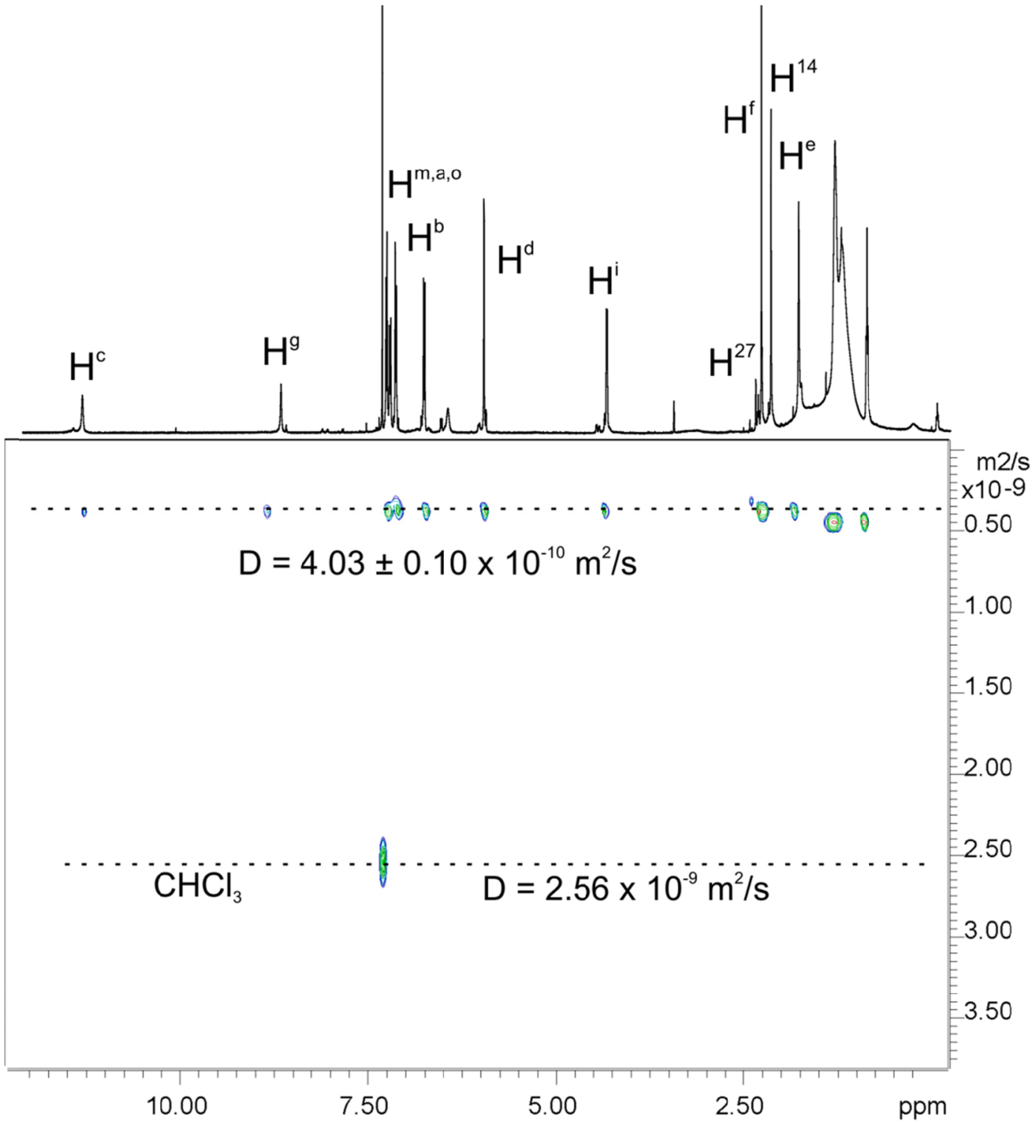

Figure S60. ^{1}H pseudo-2D DOSY plot of **12**$\subset$**1**·MTOA·Cl$^-$complex. Data fitted to a monoexponential function.

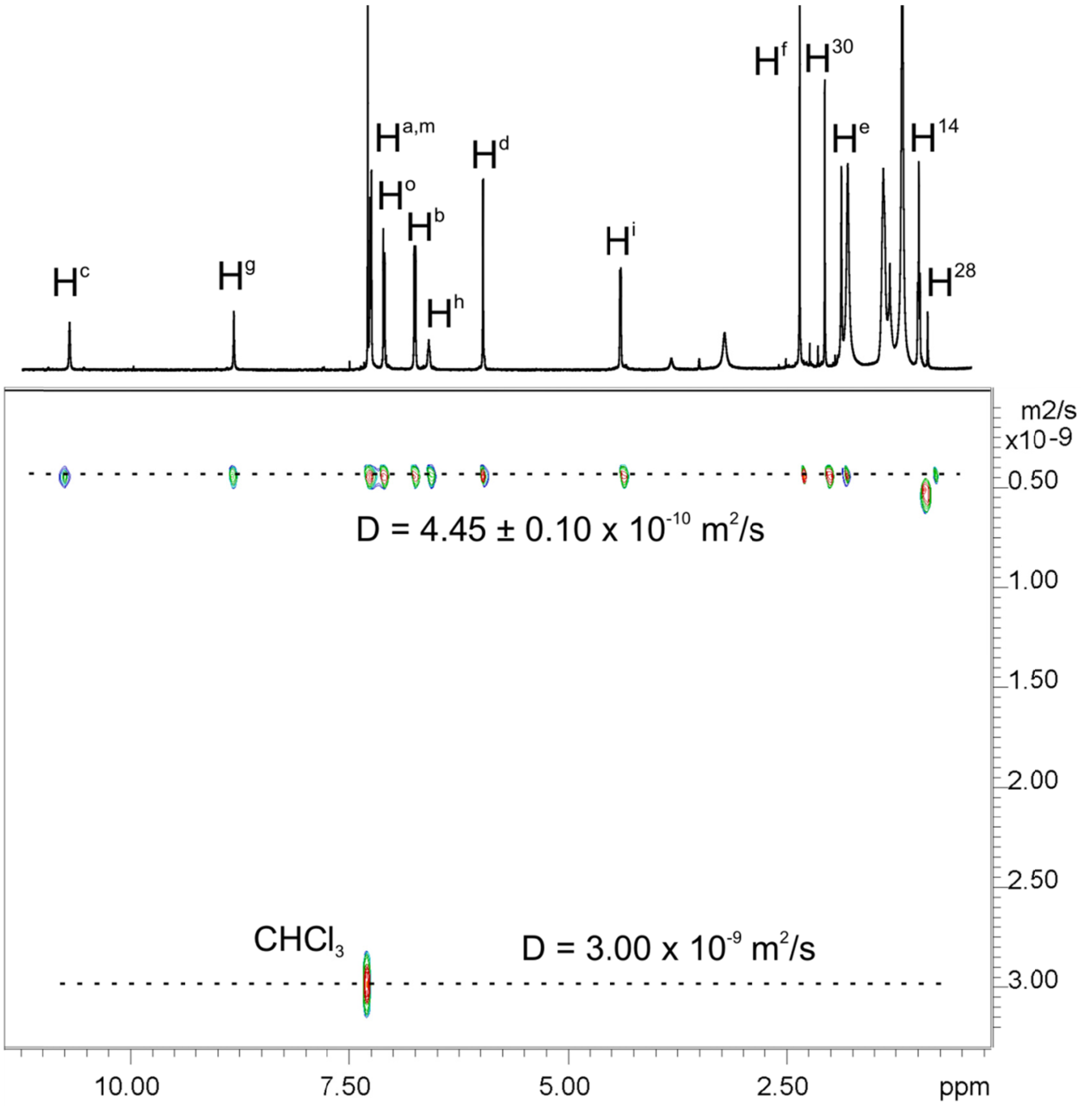

Figure S61. ^{1}H pseudo-2D DOSY plot of **13⊂1**·MTOA·Cl^{-} complex. Data fitted to a monoexponential function. See Figure S51 for proton assignment.

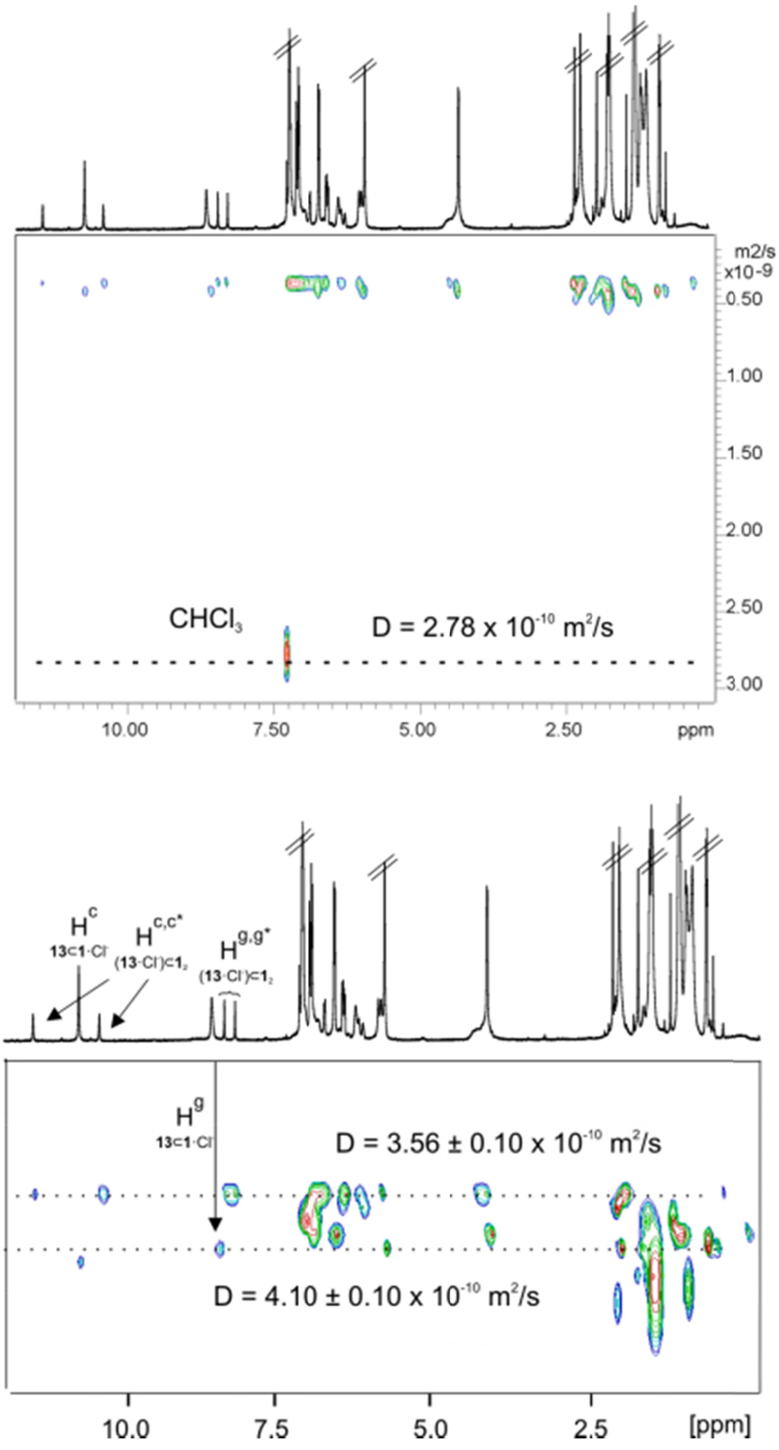

Figure S62. ^1H pseudo-2D DOSY plot of a d-chloroform mixture of tetraurea **1**, betaine **13** and MTOACl in a 2:1:1 ratio and expansion. Data fitted to monoexponential functions. See Figure S51 for proton assignment.

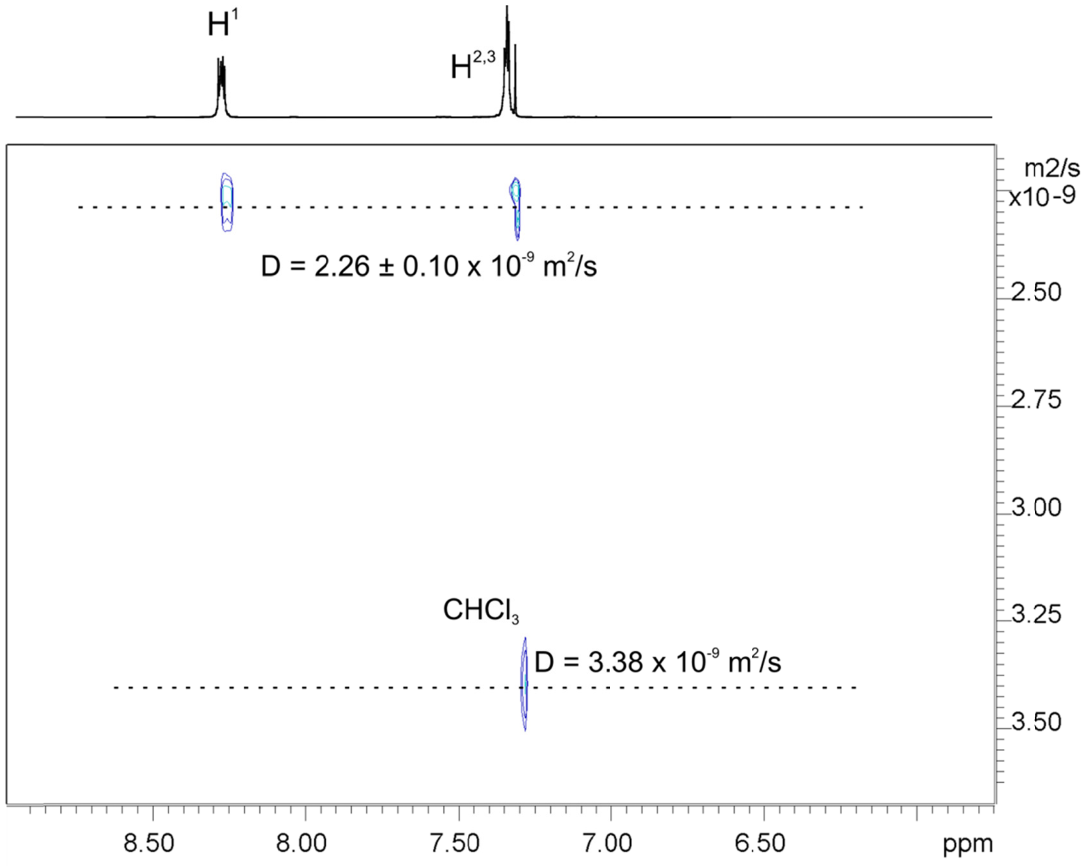

Figure S63. [1]H pseudo-2D DOSY plot of a $CDCl_3$ solution of pyridine *N*-oxide **5**. Data fitted to a monoexponential function. See Figure S2 for proton assignment.

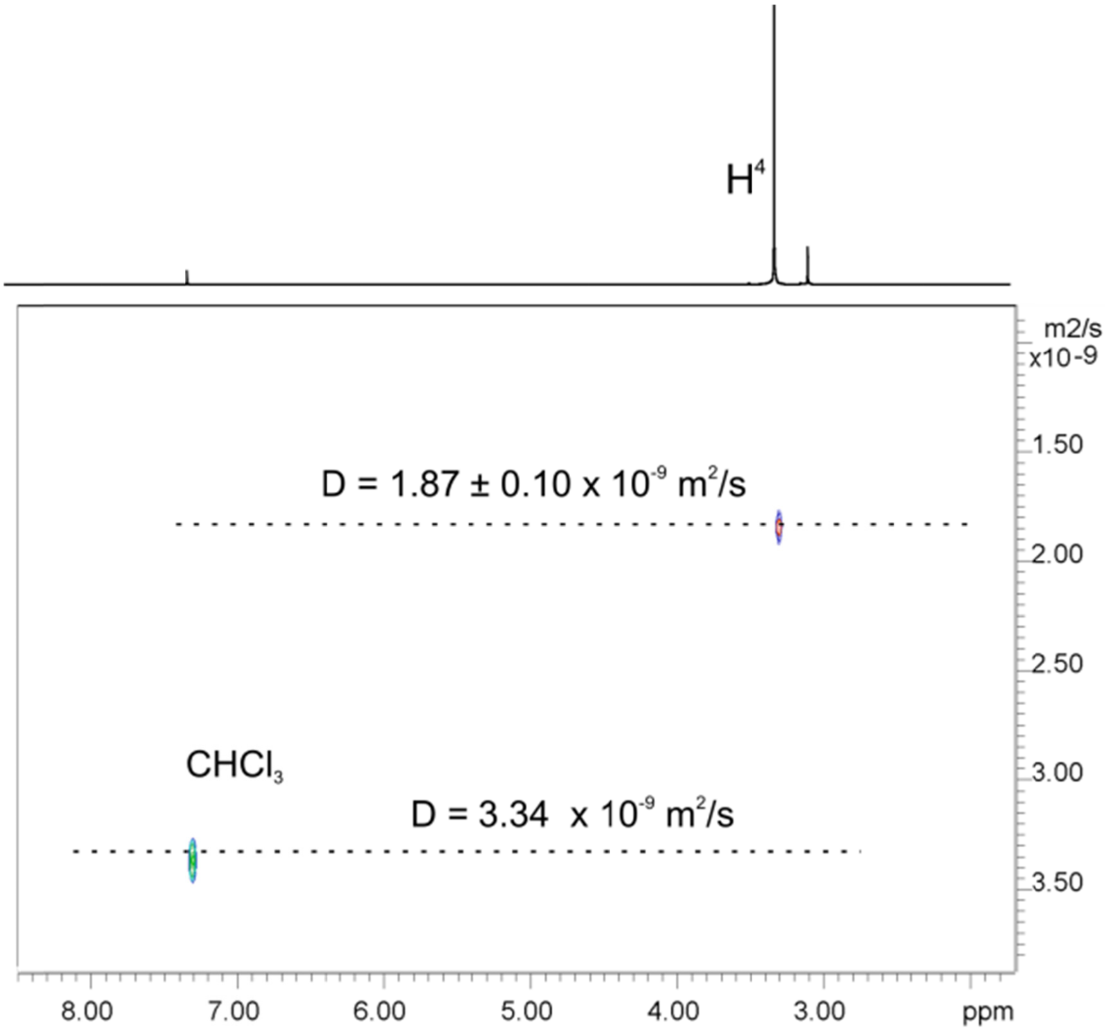

Figure S64. [1]H pseudo-2D DOSY plot of a $CDCl_3$ solution of trimethylamine *N*-oxide **3**. Data fitted to a monoexponential function. See Figure S25 for proton assignment.

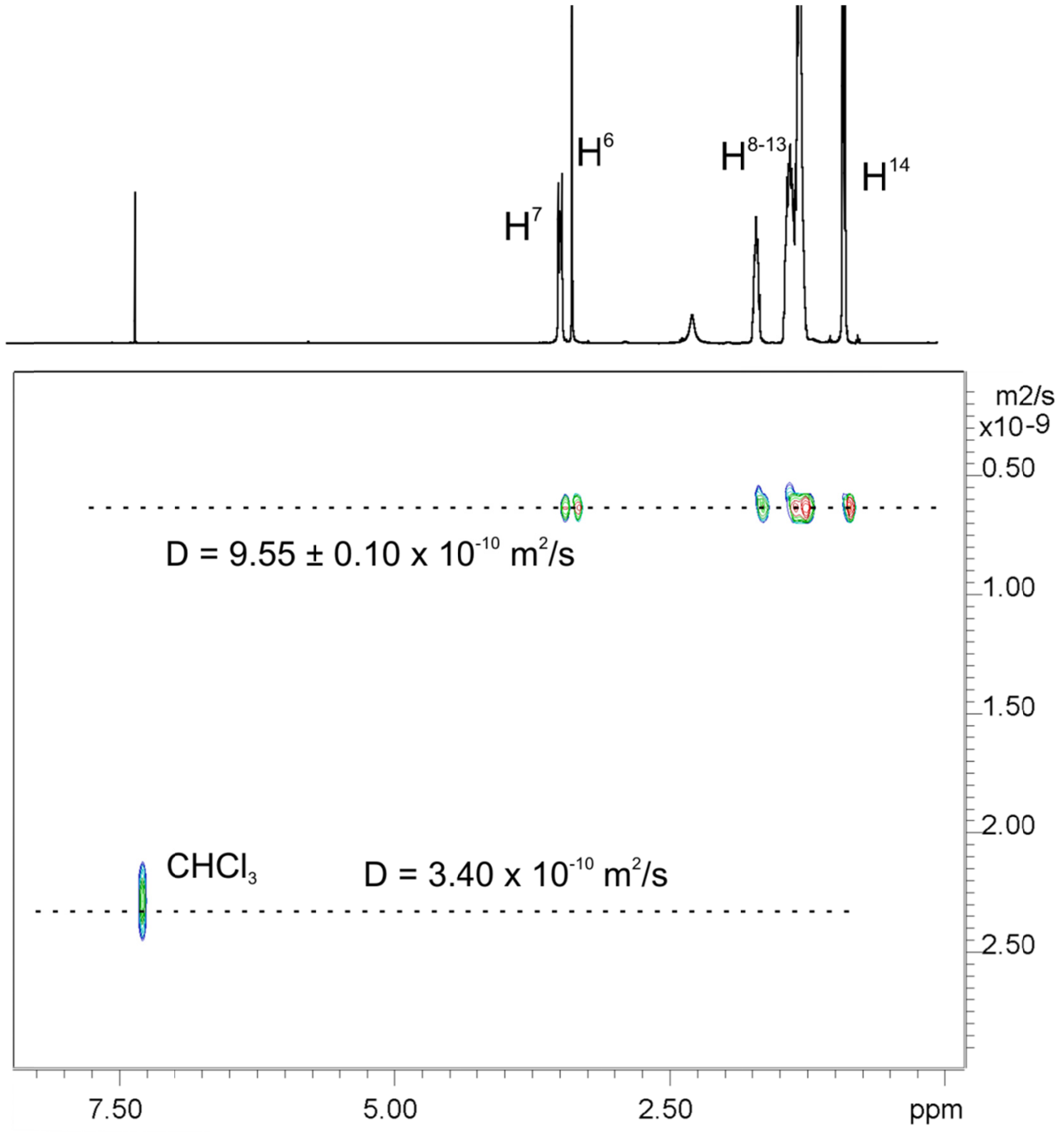

Figure S65. [1]H pseudo-2D DOSY plot of a $CDCl_3$ solution of MTOACl. Data fitted to a monoexponential function. See Figure S2 for proton assignment.

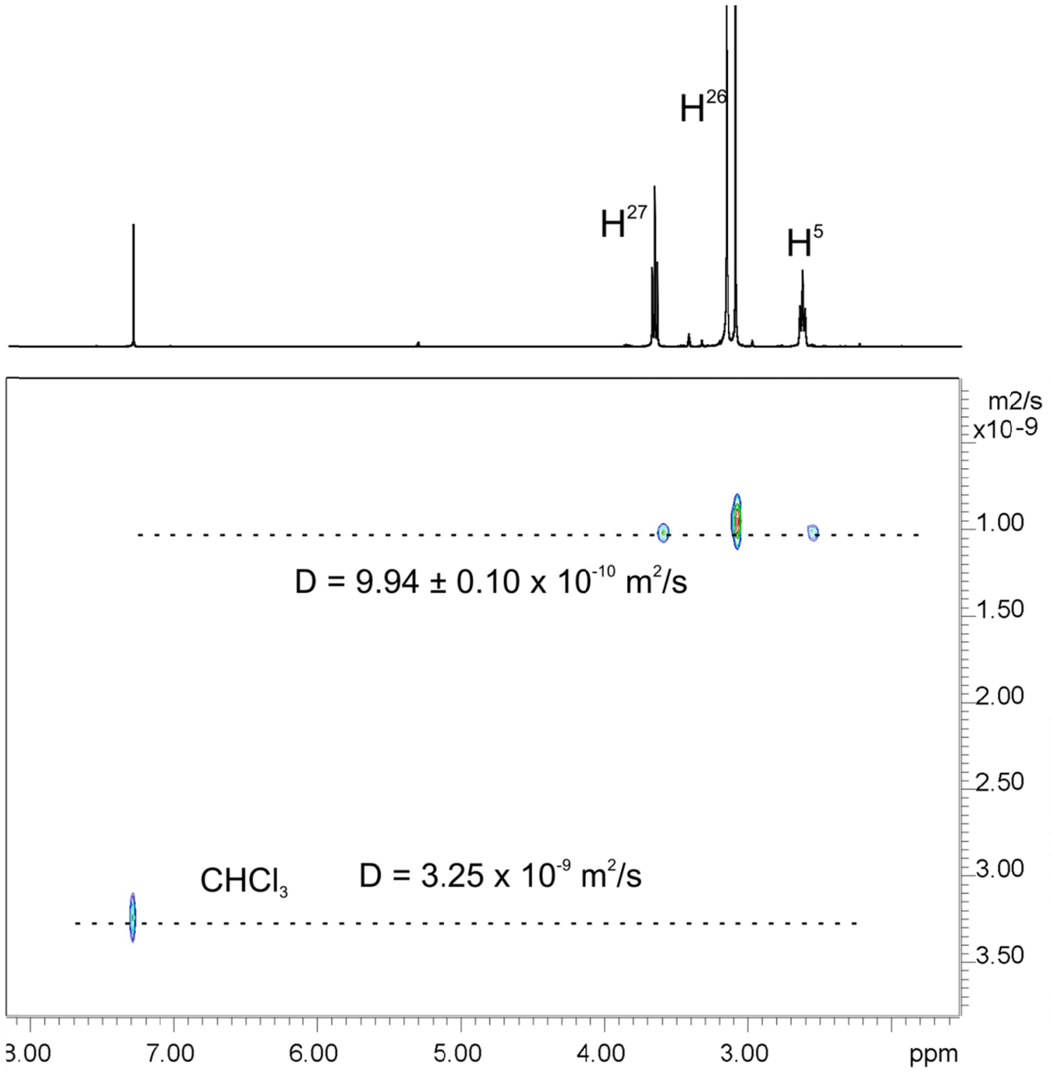

Figure S66. ^{1}H pseudo-2D DOSY plot of a $CDCl_3$ solution of betaine **12**. Data fitted to a monoexponential function. See Figure S40 for proton assignment.

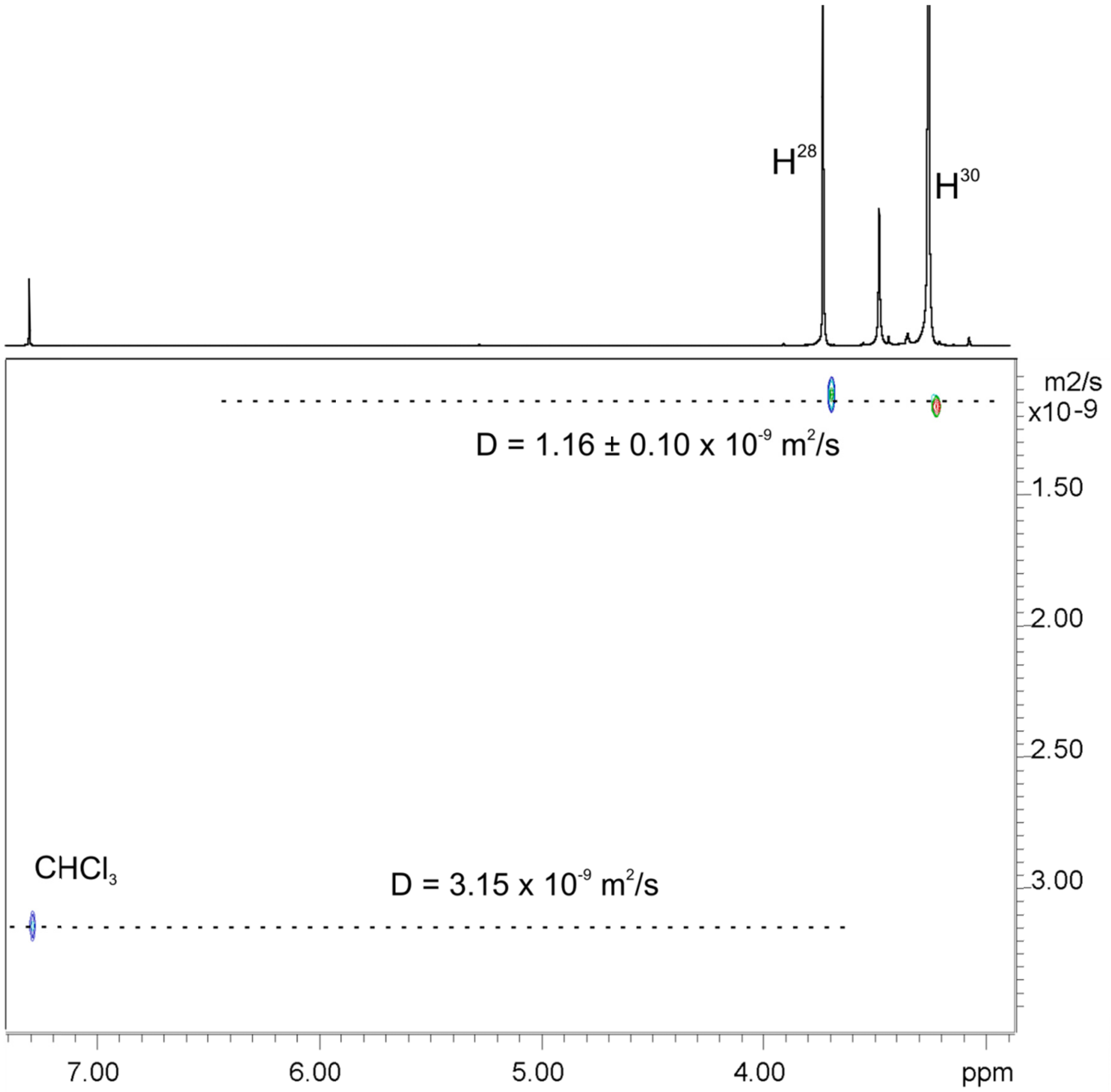

Figure S67. [1]H pseudo-2D DOSY plot of a $CDCl_3$ solution of betaine **13**. Data fitted to a monoexponential function. See Figure S51 for proton assignment.

Energy minimized structures and packing coefficients

Energy minimized (MM3) structures of dimeric capsular assemblies. Cavity volumes and guest volumes calculated with the software SwissPDB. A volume of 39.35 Å^3 was estimated for the chloride anion.[6]

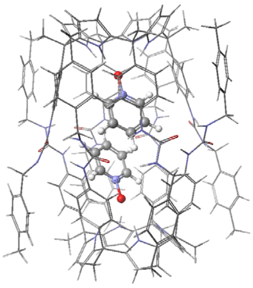

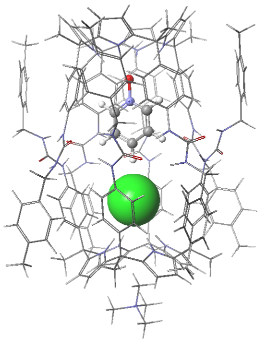

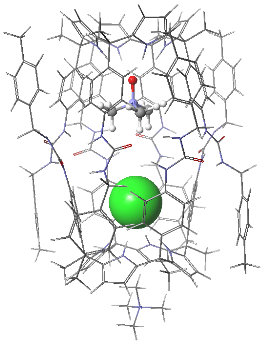

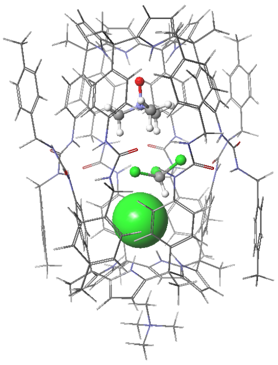

	$\mathbf{5}_2\subset\mathbf{1}_2$	$\mathbf{5}\cdot Cl^-\subset\mathbf{1}_2$	$\mathbf{3}\cdot Cl^-\subset\mathbf{1}_2$	$\mathbf{3}\cdot CHCl_3\cdot Cl^-\subset\mathbf{1}_2$
Cavity volume (Å^3)	338	289	314	327
Guest volume (Å^3)	170	124	122	191
PC	0.50	0.43	0.39	0.58

[6] C. Estarellas, A. Bauza, A. Frontera, D. Quinonero and P. M. Deya, *Phys. Chem. Chem. Phys.*, 2011, **13**, 5696-5702.

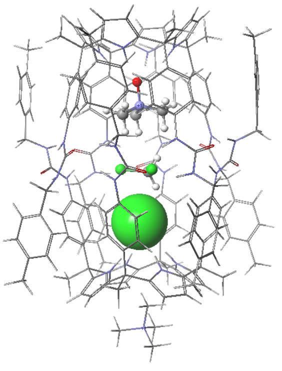

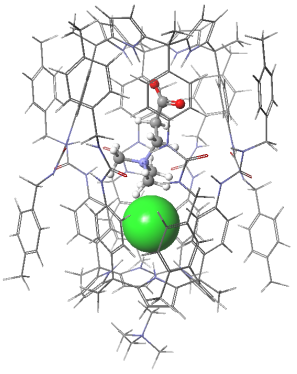

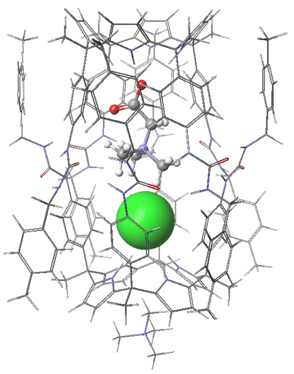

	**3**·CH_2Cl_2·Cl^-⊂$\mathbf{1}_2$	**12**·Cl^-⊂$\mathbf{1}_2$	**13**·Cl^-⊂$\mathbf{1}_2$
Cavity volume (Å^3)	321	309	296
Guest volume (Å^3)	179	172	158
PC	0.55	0.55	0.53

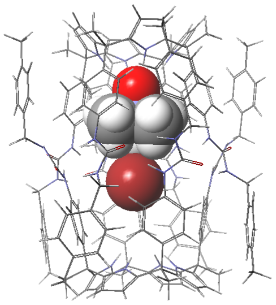

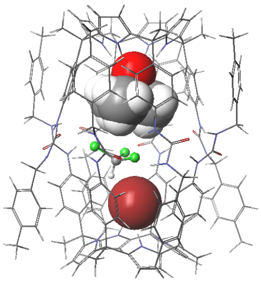

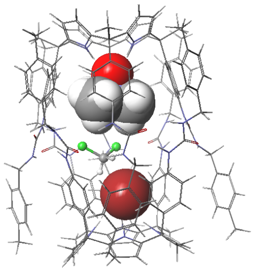

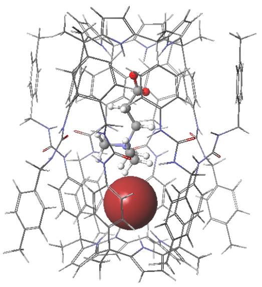

	**3**·Br^-⊂$\mathbf{1}_2$	**3**·$CHCl_3$·Br^-⊂$\mathbf{1}_2$	**3**·CH_2Cl_2·Br^-⊂$\mathbf{1}_2$	**12**·Br^-⊂$\mathbf{1}_2$
Cavity volume (Å^3)	299	327	328	318
Guest volume (Å^3)	137	206	191	187
PC	0.45	0.63	0.58	0.58
